# Sediment burial reduces coral larval settlement, recruit survivorship, and inhibits the recovery of coral populations

**DOI:** 10.1038/s42003-026-10370-1

**Published:** 2026-06-17

**Authors:** Victor Rodriguez-Ruano, Alain Duran, Allan J. Bright, Kathryn Grazioso, Sophia Ippolito, Colin Murphy, Dana E. Williams, Jolisa Velazquez, William Barriera, Silvana Guzman, Mark C. Ladd

**Affiliations:** 1 Cooperative Institute for Marine and Atmospheric Studies, Rosenstiel School of Marine, Atmospheric, and Earth Science, University of Miami, Miami, FL USA; 2 Population and Ecosystems Monitoring Division, NOAA Southeast Fisheries Science Center, Miami, FL USA; 3 Institute of the Environment, Department of Biological Sciences, Florida International University, Miami, FL USA

**Keywords:** Population dynamics, Ecosystem ecology

## Abstract

The recovery of coral populations depends on successful settlement and survivorship of recruits. Sedimentation is a major stressor for corals, yet information on its influence on Caribbean juvenile corals is limited. We evaluated larval settlement and recruit survival under sediment burial across six Caribbean species and multiple early life stages. Burial of suitable substrata under 2 mm of sediment reduced settlement probability to ≤25% and 4 mm suppressed settlement entirely. Similarly, the burial of coral recruits under 4 mm of sediment decreased survival probability to 0–31% within ten days and increased mortality risk 3- to 25-fold relative to unburied corals. Field surveys across the Florida Reef Tract corroborate that sediment trapped within long, sediment-laden algal turf (LSAT) is a significant, negative predictor of the presence of coral recruits. Our results reflect the urgent need for managers to limit future declines in reef-habitat quality to promote coral recovery.

## Abstract (web summary)

Sediment loads present on Florida’s reefs are impeding the recovery of coral populations by inhibiting larval settlement and reducing the survivorship of recruits for multiple reef-building coral species.

## Introduction

Coral populations are in decline worldwide as a result of global and local stressors that continue to increase the frequency and severity of lethal and sublethal disturbances for corals^1–5^. Mass mortality events from thermal stress are the primary drivers of global coral loss^6–8^, compounded by regional losses from stressors such as disease outbreaks^9,10^. Although large-scale global disturbances are responsible for the most widespread coral mortality, more localized stressors like nutrient pollution^11,12^, hypoxia^13^, and sedimentation^14–16^ can act independently and synergistically with global stressors to amplify the magnitude of coral mortality events^17,18^.

Larval settlement, survival, and growth into reproductive maturity (i.e., recruitment) are fundamental to the recovery of coral populations, especially after mass-mortality events^19–22^. Coral recruitment is a complex process that entails multiple steps, including gamete release, fertilization, larval development, transport, settlement, recruit survival, and growth of corals^23,24^. While settlement success depends on a multitude of factors^25^, the availability of suitable substrata is a consistent, significant predictor of settlement success across reef habitats^26,27^. For example, high recruitment resulting from sufficient larvae and available settlement substrata allowed coral populations on the forereefs around Moorea, French Polynesia, to recover rapidly after back-to-back disturbance events reduced coral cover to nearly 0% (refs. ^20,28^). Similarly, healthy assemblages of herbivorous fish on the Caribbean reefs of Bonaire correlated with a decrease in macroalgal populations after coral cover declined by 22% due to Hurricane Omar in 2008 and a bleaching event between 2007 and 2011. In turn, the abundance of suitable settlement substrata allowed for the recruitment of new individuals, growth of extant corals^21^, and recovery of corals to pre-disturbance levels (>45% coral cover; ref. ^21^) by 2017. While larval supply, herbivory, and suitable substrata can drive patterns of large-scale coral recruitment, other biotic and abiotic factors like water flow, microhabitat conditions, the position of substrata, roughness, color, and benthic composition^29–31^ can determine small-scale patterns in recruitment^32^.

Recent work suggests that survival of corals during the first 12 months post-settlement may be a more critical driver of coral recruitment and population recovery than absolute settlement rates^22^. Coral recruits are susceptible to mortality from a myriad of stressors, given their small size and limited resource provisioning^33–35^. For example, allelopathy or overgrowth by competitors^36,37^, incidental grazing by herbivores^38^, and predation^39^ can all contribute to recruit mortality. More recently, marine heatwaves have emerged as a major stressor limiting recruit survival and coral recovery globally^40–42^. Thus, corals must overcome several key bottlenecks within the first ca. 12 months of their lifespan to contribute to population recovery. Consequently, beyond influencing existing coral populations, disturbances can impact future generations via significant declines in settlement success or post-settlement survivorship of juveniles^43–45^.

Sedimentation and turbidity are ubiquitous stressors affecting tropical reefs worldwide and are byproducts of disturbances such as coastal runoff, port dredging, and increased bioerosion of the reef framework^14,46–49^. The effects of sedimentation—i.e., sediments deposited onto the reef substratum—on individual corals include altered growth rates^50–52^, reduced reproductive output^53–55^, and partial or whole colony mortality^51,56,57^. Sedimentation can manifest as an acute or chronic stressor, with impacts on corals dependent upon the duration, magnitude, and return time of sediment stress events^51,56,58^, as well as sediment characteristics such as grain size and source material^59,60^. Moreover, these impacts vary based on inter- and intraspecific differences in corals’ tolerance to sediment stress^58,61,62^. Nevertheless, persistent sedimentation has historically suppressed reef development and increased sediment fluxes in tandem with other disturbances that can further exacerbate reef degradation^46,63,64^.

Concurrent with decreasing coral populations on tropical reefs across the globe has been an increase in benthic space-holders unsuitable for the settlement and survival of coral recruits^65–67^. One such group is long sediment-laden algal turf (LSAT; refs. ^27,68,69^), which develops when there is an increase in the accumulation and retention of sediments on short productive algal turf (1–3 mm tall). Evidence suggests that LSAT is increasing in abundance on reefs worldwide^27,70^. Although the retention of sediments by LSAT can reduce sediment resuspension, these deposited sediments can also interfere with settlement cues for coral larvae^71^, make turf algae unpalatable to grazers^72^, and likely reduce the survivorship of coral recruits^73,74^.

Sedimentation impacts on Indo-Pacific coral recruits are well documented (e.g., ref. ^14^). Although studies documenting the impacts of sediments on corals have been conducted in the Caribbean^75–80^, the impact of sedimentation across multiple reef-building species and early life stages remains a significant knowledge gap in the region^81^. Given the potential effects of sedimentation on larval settlement and recruit survivorship^14,49^, sedimentation may constitute a significant but underappreciated impediment to the recovery of increasingly disturbed Caribbean coral reefs^69,74,79,82,83^. Yet studies that focus on the early life-history stages of Caribbean corals, the life stages at which they are likely the most vulnerable to the impacts of sedimentation^49,75–77^, are sparse^78,79^. Although field observations have documented the effects of sediment burial on coral populations, no experimental studies that we are aware of have isolated the effects of sediment depth or burial duration on coral settlement and recruit survivorship across Caribbean species. As sedimentation and sediment-dependent phenomena such as LSAT continue to proliferate globally^27,70^, understanding the effects of sedimentation on the early life stages of corals is essential to assess its influence on coral population recovery, especially within the context of Caribbean reefs. The last decade was particularly devastating for Caribbean reefs^84,85^, and the recovery of coral populations has been limited^18,32,86^. Therefore, understanding the mechanisms by which sediment impedes the recovery of coral populations is necessary to inform management strategies that mitigate future sediment stress and maximize coral recovery.

Here, we present results of experimental tests on the effects of sediment presence, depth, burial duration, and source on larval settlement and recruit survival of six key reef-building species of Caribbean corals: *Acropora cervicornis*, *Colpophyllia natans*, *Diploria labyrinthiformis*, *Orbicella faveolata*, *Pseudodiploria clivosa*, and *Pseudodiploria strigosa*. Our main objective was to understand the effects of sediment burial across multiple coral species and early life stages. To understand how sedimentation influences larval settlement, we conducted experiments testing how sediment load and burial depth influence settlement probability. We assessed how sediment burial influences the survival of recently settled coral recruits by performing a series of experiments testing burial depth and duration on the survivorship of coral recruits aged 1 to 18 months. Furthermore, we paired our aquaria-based experiments with field surveys on shallow reef habitats of southeast Florida and the upper Florida Keys, USA, to determine the relationship between LSAT and the abundance of coral recruits. We predicted that settlement success would be inversely related to sediment depth. Similarly, we expected that coral recruit survivorship would decrease with increasing sediment depth and duration, and that survivorship would be higher for older and larger corals.

We found that burial of the substratum and corals under small amounts of sediment (150–300 mg cm^−2^, equivalent to sediment layers 2–4 mm deep) caused drastic reductions in coral settlement and survivorship. These effects were consistent across species and life stages. The levels of sedimentation we used in our experiments reflect those we observed on Florida’s reefs: average LSAT sediment depth ranged between 1 and 3 mm across sites with sediment loads ranging from 50 to 150 mg cm^−2^. Contrary to our predictions, recruit survivorship did not increase with coral age, suggesting that corals do not escape the impacts of sedimentation via size in their first 18 months of life. Results from our field surveys supported our experimental findings and indicated that the probability of encountering a juvenile coral drops below 25% when the LSAT sediment layer exceeds 4 mm. These findings highlight the scale at which management agencies should consider sedimentation relevant for impacts to corals (i.e., mm-scale impacts), the need for avoiding and minimizing anthropogenic sediment sources (dredging, terrestrial run-off, etc.), and the major influence of sedimentation as a bottleneck to the recovery of coral populations^49,87^.

## Results

### Settlement assays

**Fig. 1:**
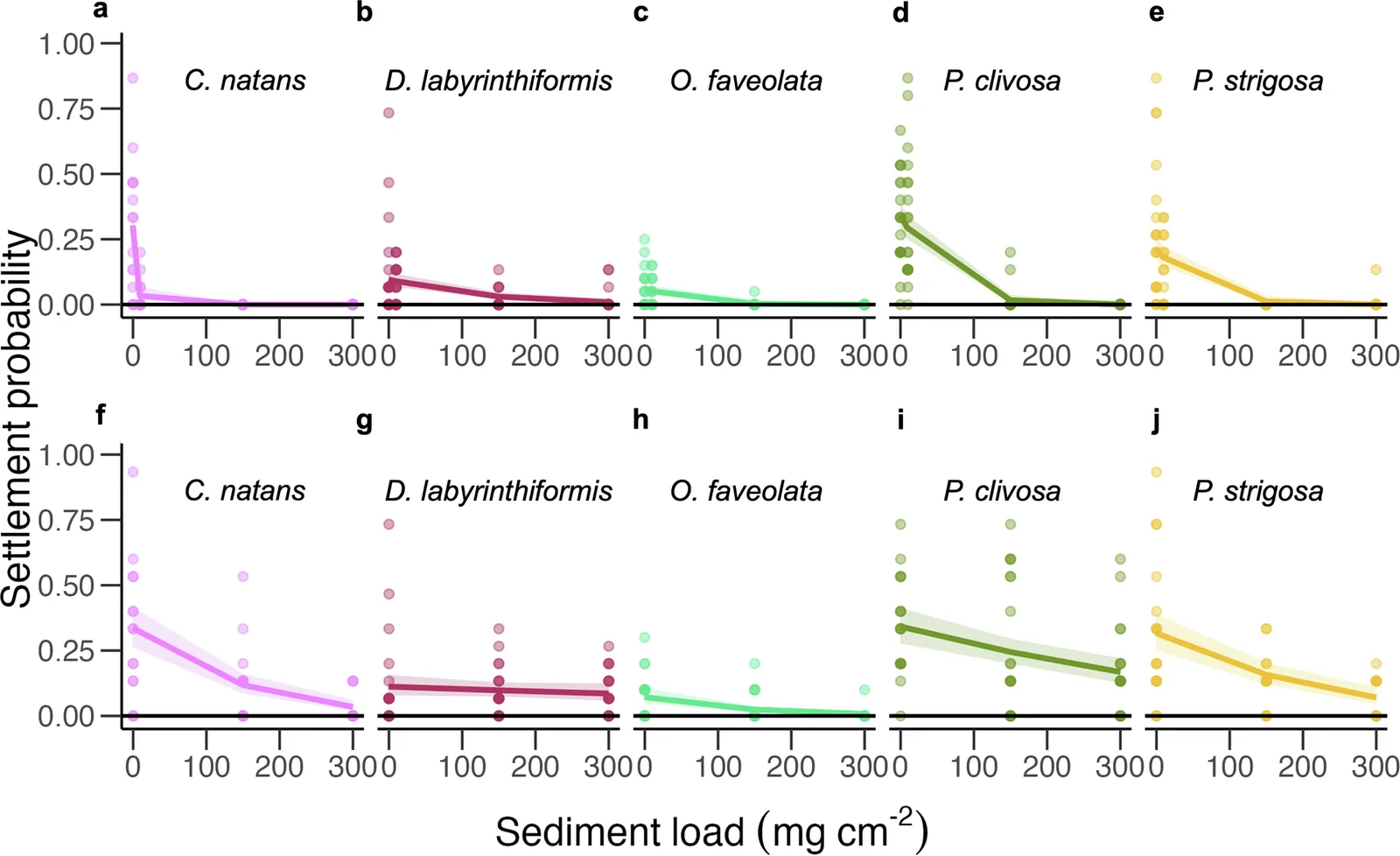
Declines in larval settlement probability of multiple Caribbean coral species on substrata buried or surrounded by sediment. Scatterplots depict **a**–**e** the binary response of the settlement of coral larvae to ceramic tiles buried under varying sediment loads (*n* = 60; mg cm^−2^) and **f**–**j** the binary response of the settlement of coral larvae to ceramic tiles surrounded but not buried by varying sediment loads (i.e., sediment presence; mg cm^−2^; *n* = 45). The color-coded curves depict the best-fit logistic generalized mixed-effects models that predict the probability of settlement for each species across the sedimentary gradient; the shaded areas represent the 95% confidence intervals of the models. Each data point represents a single replicate, which consists of the glass chamber with a ceramic tile, 10–15 coral larvae, and a designated sediment load between 0–300 mg cm^−2^.

The burial of potential settlement substratum by sediment significantly decreased the probability of larval settlement for all five coral species we tested (Fig. 1a–e and Table S1). The predicted probability of larval settlement for four of the five species—*Colpophylia natans, Orbicella faveolata, Pseudodiploria clivosa*, and *P. strigosa*—was reduced to zero when the settlement substratum was buried under 4 mm of sediment (equivalent to a sediment load of 300 mg cm^−2^). *Diploria labyrinthiformis* was the only species with a settlement probability >0 under the 4 mm burial treatment, maintaining a 1% probability of settlement under the 4 mm burial treatment (Fig. 1b). Although burial under 4 mm caused the largest reductions in settlement, the burial of settlement substrata under 2 mm (equivalent to a sediment load of 150 mg cm^−2^) also severely reduced the probability of larval settlement for most species, lowering settlement probability by 97–100%. Although the burial of substrate negatively affected all species, there were significant differences in settlement success among them (Table S1). *Colpophyllia natans* larvae were the most sensitive to sediment burial—10 mg cm^−2^ of sediment (<50% of the settlement substratum covered in <1 mm of sediment) reduced the probability of settlement to 3%, and 2 mm of sediment burial lowered settlement probability to zero (Fig. 1a).

When isolating the effect of the presence of sediment in our settlement chambers (i.e., sediment in the chamber but not covering the settlement substratum), we found that sediment presence alone decreased the probability of settlement for three out of the five species we tested: *C. natans, O. faveolata*, and *P. strigosa* (Fig. 1f, h, j and Table S2). Larvae of *C. natans* appeared particularly sensitive to the presence of sediment, with 300 mg cm^−2^ reducing the probability of settlement by 91% relative to the control trials. There was no effect of sediment presence on the probability of settlement for *D. labyrinthiformis* or *P. clivosa* larvae (Table S2).

We conducted assays to test the influence of sediments on water quality after 72 h. Water quality varied significantly among treatments (permuted *F*_5,24_ = 11.23, *R*^2^ = 0.70, *p* = 0.0001; Fig. S1); however, post hoc tests revealed that the only significant differences in water quality parameters were between the ammonia and nitrate concentrations for the Key Largo (KL) 2 mm treatment (ammonia after 72 h = 0.024 ± 0.015 mg L^−1^, nitrate after 72 h = 1.46 ± 0.02 mg L^−1^) compared to the KL sprinkle treatment (ammonia after 72 h = 0.000 ± 0.000 mg L^−1^, nitrate after 72 h = 1.44 ±0.04 mg L^−1^; Tables S3 and S4). The Port Everglades 4 mm treatment exhibited the highest levels of ammonia (0.192 ± 0.050 mg L^−1^) and nitrite (0.0132 ± 0.0030 mg L^−1^), but it was also the treatment with the highest variability (Table S3 and Fig. S1).

### Sediment burial assays

**Fig. 2:**
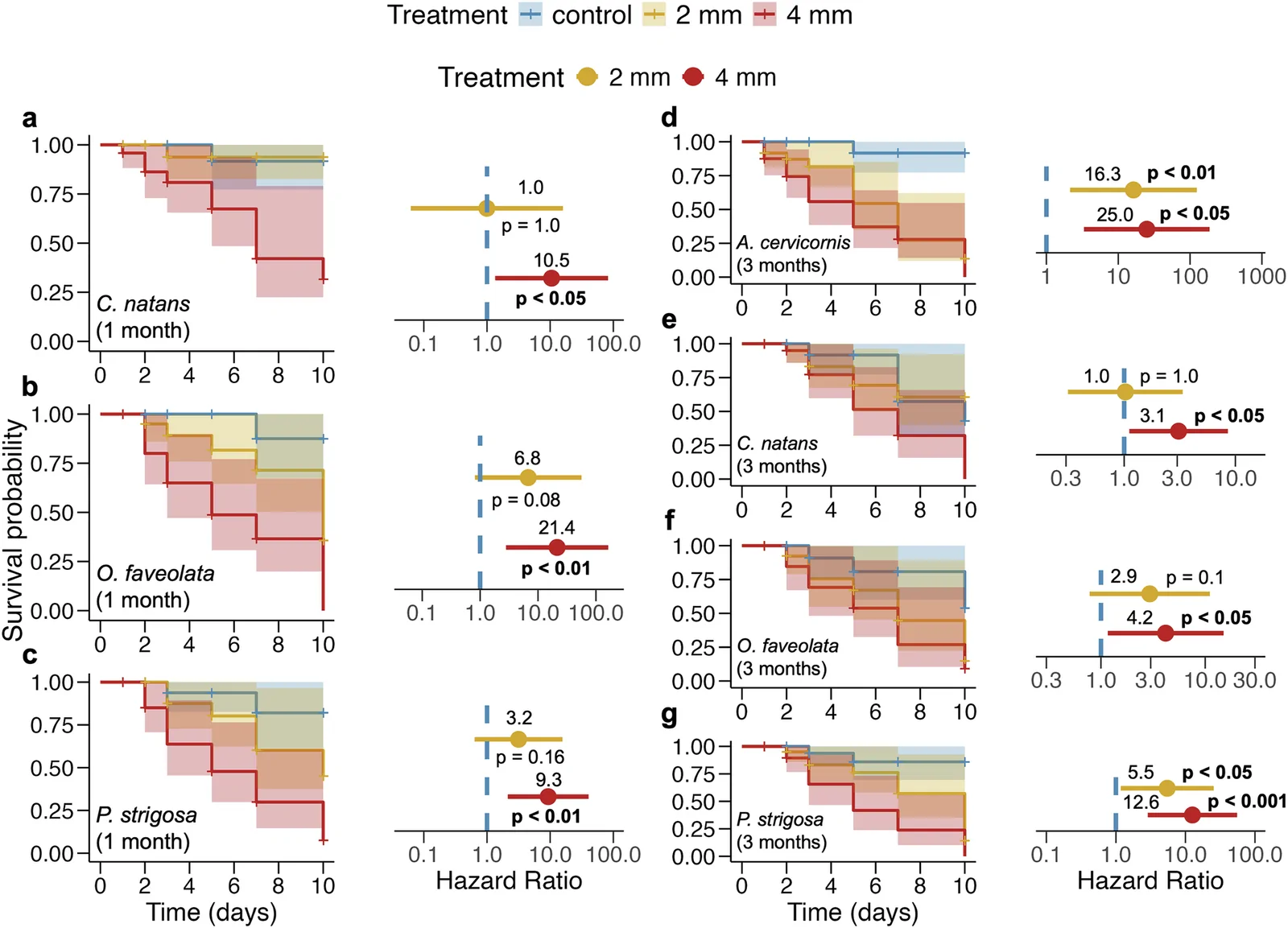
Survival trends of young recruits (1–3 months old) of multiple Caribbean coral species under sediment burial treatments across a timespan of 10 days. Kaplan–Meier curves and forest plots depict survival trends of 1-month- and 3-month-old recruits of four different coral species: **a** 1-month-old *C. natans*, **b** 1-month-old *O. faveolata*, **c** 1-month-old *P. strigosa*, **d** 3-month-old *A. cervicornis*, **e** 3-month-old *C. natans*, **f** 3-month-old *O. faveolata*, **g** 3-month-old *P. strigosa* (*n* = 24 per species and treatment). Kaplan–Meier curves depict recorded mortality events through time and forest plots depict the time-independent hazard ratios calculated by the Cox-proportional hazards models for each treatment. The dotted vertical lines represent a hazard ratio of 1. Any hazard ratio greater than 1 indicates increased mortality risk under the respective treatment. The error bars depict the 95% CIs of the hazard ratios, which, if greater than 1, indicate a significant (*p* < 0.05) hazard of that experimental treatment relative to the control treatment (no sediment burial); 2 mm = 150 mg cm^−2^, 4 mm = 300 mg cm^−2^.

The majority of species showed a marked decline in the survival of 1-month-old coral recruits through time when buried under 2 mm and 4 mm of sediment after 10 days. The species with the largest decline in survivorship was *O. faveolata*, whose survival probability decreased to 36% (95% CI; 0.13, 1.00) under 2 mm of sediment and 0% (95% confidence intervals are not estimable at and beyond this timepoint and are therefore reported as NA) under 4 mm of sediment (Fig. 2b). After 5 days of burial under 4 mm of sediment, the probability of survivorship dropped by 50% for recruits of *O. faveolata* (0.31, 0.77) and *P. strigosa* (0.30, 0.77), and to 0 and 7% (0.01, 0.47), respectively, after 10 days of burial under 4 mm of sediment. By contrast, the survival probability of 1-month-old *C. natans* was 94% (0.83, 1.00) after 10 days of burial under 2 mm of sediment, similar to the baseline survival probability from the control trials, but decreased to 32% (0.14, 0.74) after 10 days of burial under 4 mm of sediment (Fig. 2a). According to the Cox-PH models for all species tested, the risk of mortality for 1-month-old recruits significantly increased when buried under 4 mm of sediment for 10 days, increasing the probability of mortality compared to controls by 11- (1.35, 82.31), 21- (2.80, 162.99), and 9-fold (2.13, 40.52) for *C. natans*, *O. faveolata*, and *P. strigosa*, respectively (Fig. 2a–c).

There was a marked decline in survival over time for the 3-month-old recruits of most species when buried under 2 mm and 4 mm of sediment over a 10-day timespan. The survival probability for three species—*O. faveolata* (0.03, 0.85), *Acropora cervicornis* (0.04, 0.49), and *P. strigosa* (0.02, 0.83)—decreased to ~15% after being buried under 2 mm of sediment for 10 days. By contrast, the survival probability for 3-month old *C. natans* recruits decreased to 61% (0.40, 0.92) under the same treatment. However, all these species displayed severe declines in survivorship when buried under 4 mm of sediment, with all survival probabilities decreasing to 0% after 10 days, except for *O. faveolata* (9%; 0.014, 0.576; Fig. 2d–f). Accordingly, the burial of 3-month-old recruits under 4 mm of sediment for 10 days significantly increased the relative risk of mortality for all species, ranging from a 3-fold (1.11, 8.56; *C. natans*) to a 25-fold (3.34, 186.80; *A. cervicornis*) increase compared to the mortality observed in the control trials. Additionally, the burial of the 3-month-old recruits of *A. cervicornis* and *P. strigosa* under 2 mm for 10 days also resulted in significant increases in mortality risk of 16- (2.14, 123.8) and 6-fold (1.17, 25.49), respectively, relative to the controls (Fig. 2d–f).

**Fig. 3:**
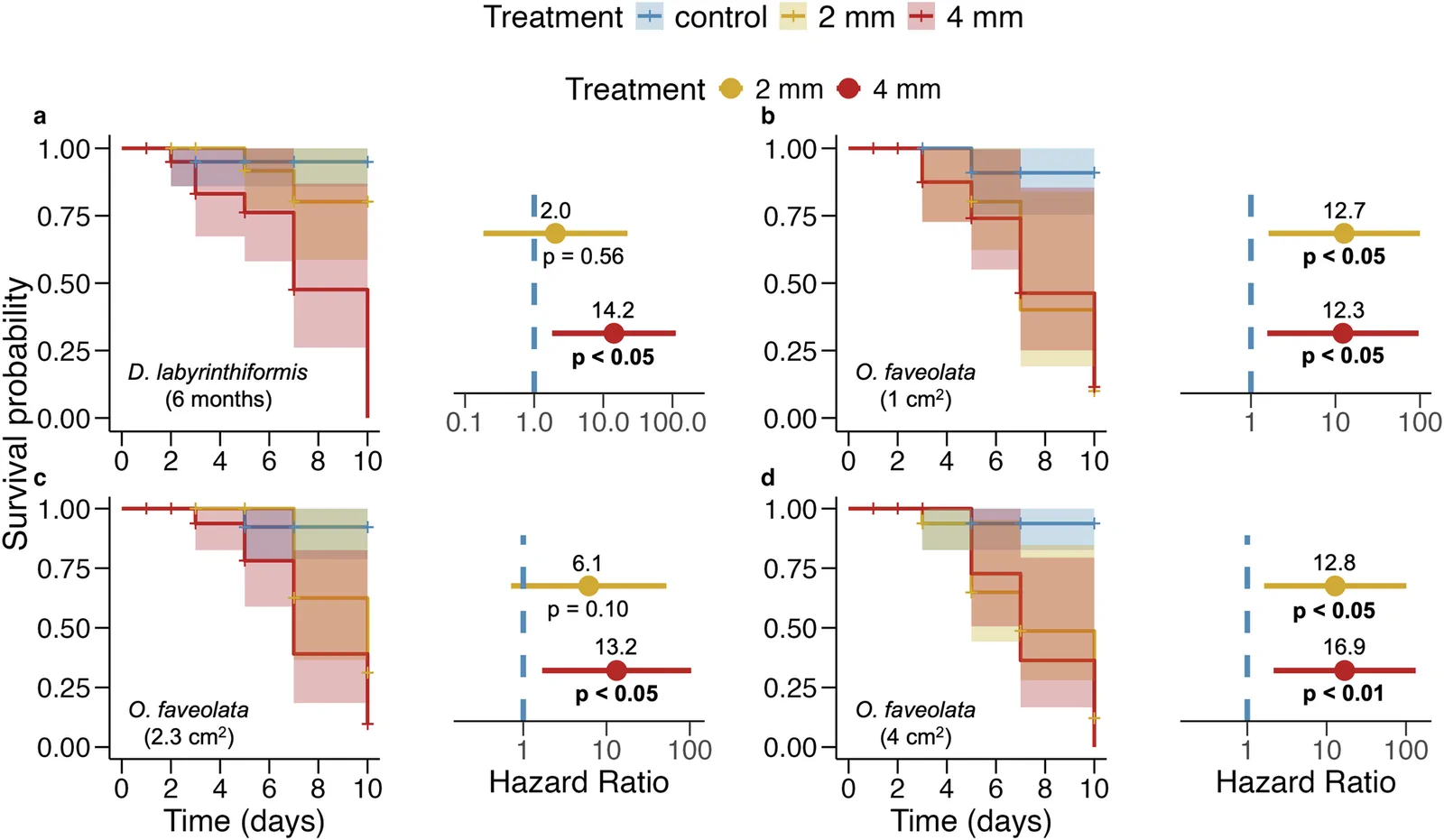
Survival trends of 6-month-old *D. labyrinthiformis* recruits and *O. faveolata* fragments representative of older recruits (1 cm^2^ = 6 months old, 2.3 cm^2^ = 12, 4 cm^2^ = 18 months old) of multiple Caribbean coral species under sediment burial treatments across a timespan of 10 days. Kaplan–Meier curves and forest plots depict survival trends of 6-month-old recruits of **a** *D. labyrinthiformis* and **b** 1 cm^2^ fragments, **c** 2.3 cm^2^ fragments, **d** and 4 cm^2^ fragments of *O. faveolata* under different sediment burial treatments across a timespan of 10 days (*n* = 24 per species and treatment). Kaplan–Meier curves depict recorded mortality events through time, and forest plots depict the time-independent hazard ratios calculated by the Cox-proportional hazards models for each treatment. The dotted vertical lines represent a hazard ratio of 1. Any hazard ratio greater than 1 indicates increased mortality risk under the respective treatment. The error bars depict the 95% CIs of the hazard ratios, which, if greater than 1, indicate a significant (*p* < 0.05) hazard of that experimental treatment relative to the control treatment (no sediment burial); 2 mm = 150 mg cm^−2^, 4 mm = 300 mg cm^−2^.

The 6-month-old recruits of *D. labyrinthiformis* showed a marked decline in survival when buried under 4 mm of sediment, with the survival probability decreasing to 0% after 10 days. Additionally, mortality risk was 14-fold (1.82, 111.86) higher relative to the controls when buried under 4 mm (Fig. 3a). The fragments of *O. faveolata* representative of 6-, 12- and 18-month-old recruits showed similar sensitivity to burial by sediment: their survival probability decreased to 10 (0.02, 0.64), 31 (0.10, 0.96), and 12% (0.02, 0.73), respectively, when buried under 2 mm of sediment for 10 days. The mortality risk of coral fragments 1 cm^2^ (representative of 6-month-old individuals) and 4 cm^2^ (representative of 18-month-old individuals) in size increased 13-fold (1 cm^2^ = 1.56, 96.25; 2 cm^2^ = 1.65, 101.8) relative to the controls when buried under 2 mm of sediment (Fig. 3b, c). When buried under 4 mm of sediment, survival probability after 10 days was between 12% (1 cm^2^ fragments) and 0% (4 cm^2^ fragments). All three size groups exhibited significant increases in mortality risk, ranging from a 12-fold (2.3 cm^2^ fragments) to a 17-fold (4 cm^2^ fragments) increase relative to the controls (Fig. 3b–d).

**Fig. 4:**
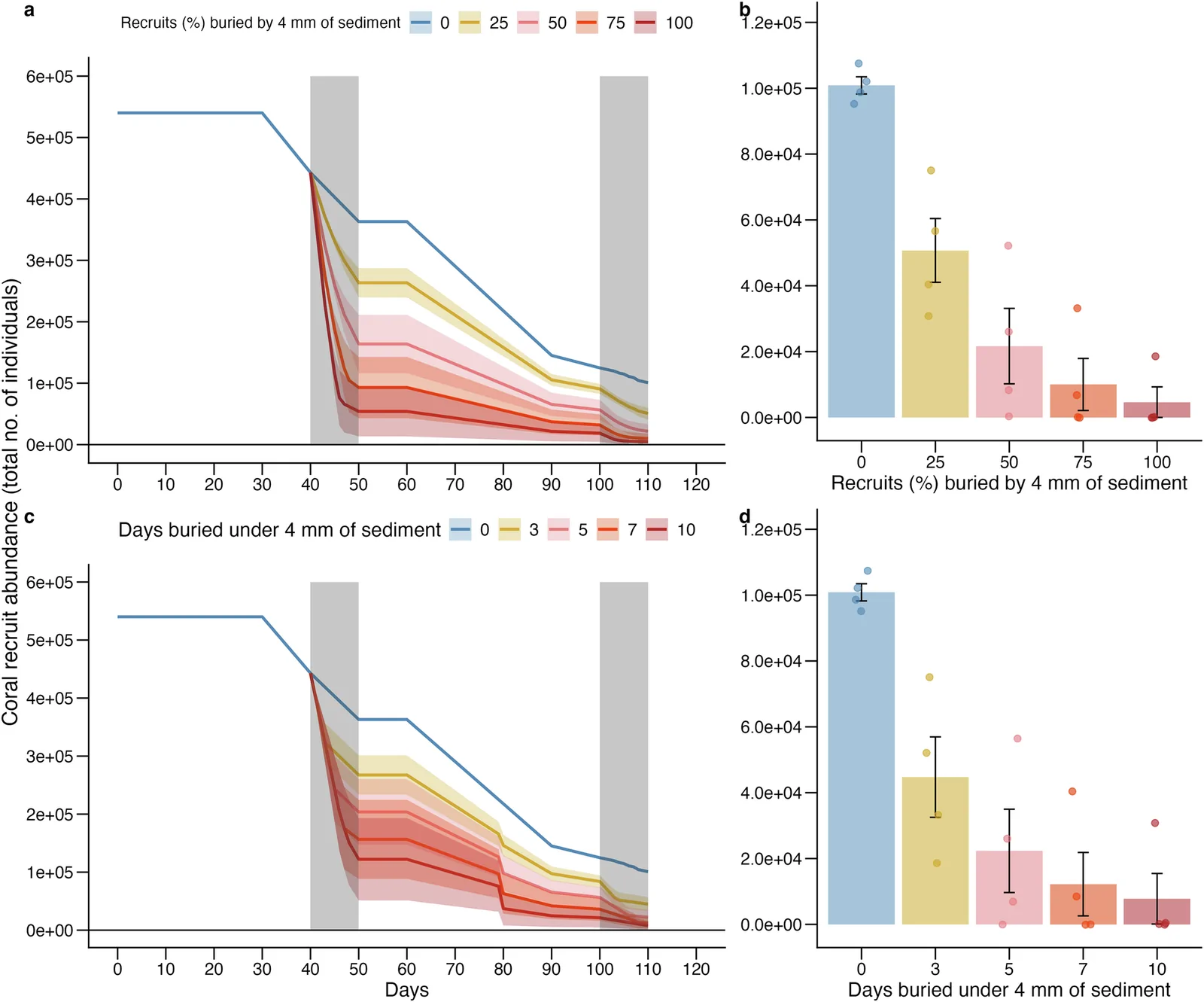
Predicting the impacts of the prevalence and duration of sedimentation events on simulated populations of coral recruits. **a** Model output for multiple scenarios simulating sedimentation events that consisted of varying proportions of the population being buried under 4 mm of sediment and **b** the predicted mean (± standard error) recruit abundance at day 110 post-settlement for each scenario (*n* = 4). **c** Model output for multiple scenarios simulating sedimentation events that resulted in varying number of days that recruits were buried under 4 mm of sediment and **d** the predicted mean (± standard error) recruit abundance at day 110 post-settlement for each scenario (*n* = 4). The shaded bars represent the time period during which sedimentation events took place. Initial coral recruit population size was estimated using the average coral recruit density from previous recruitment surveys across southeast Florida by Harper et al.^130^ on the area of hardbottom habitat around Port Everglades that has been impacted by sediment plumes during previous dredging projects (1.8 km^2^) .

Using the survival trends of *P. strigosa* recruits as a case study, we modeled multiple scenarios to predict the potential impact of two 10-day sediment burial events during the first 16 weeks post-settlement in a simulated reef space (see Supplementary Materials and Methods). Our models predict that the largest population decline would occur during the simulated sedimentation event when recruits were 1 month old. The magnitude of the decline in recruit abundance varied based on the proportion of the population buried under 4 mm of sediment and the duration of burial (0–10 days; Fig. 4a, b), with the proportion of buried recruits having a higher impact on population decline than the duration of the event (Fig. 4c, d). For example, when 25% of 1-month-old recruits were buried under 4 mm of sediment, the recruit population declined on average by 40% relative to the pre-disturbance abundance and by 88% when 100% of 1-month-old recruits were buried under 4 mm of sediment (Fig. 4a). The second 10-day sediment burial event was simulated when the assumed surviving cohort achieved 3 months of age. Here, burying 25% of recruits decreased recruit abundance to 9% of the initial recruit abundance. More strikingly, however, the burial of 100% of recruits would decrease recruit abundance to less than 1% of the initial recruit abundance (Fig. 4a).

### Field surveys

**Fig. 5:**
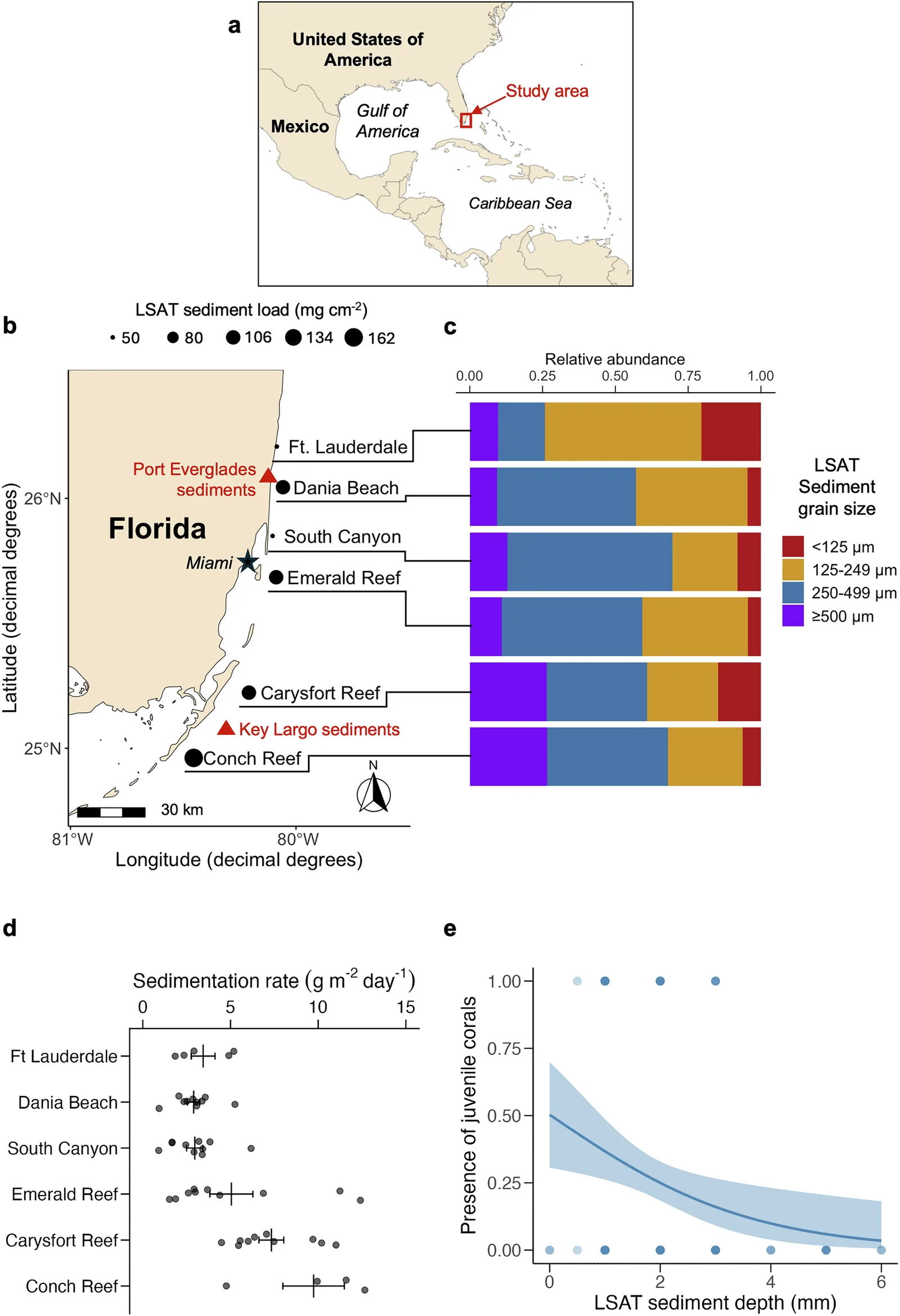
LSAT sediment load and sedimentation rates tend to increase southwards along the Florida reef tract and have a negative effect on the presence of juvenile corals. **a** Map of the greater Caribbean region in the Western Atlantic, highlighting our study area (red rectangle). **b** Map of the study area with the locations of our six study sites and two sediment collection sites. The size of the dots represents the median sediment depth (mm) of the LSAT mats at each site (*n* = 25 quadrats per site); the maroon triangles represent sediment sources for our settlement assays and sediment burial experiments. **c** Relative abundance for different grain size categories measured for sediments collected from LSAT mats. **d** crossbar plot depicting the mean (± standard error) and raw data points of the sedimentation rate (g m^−2^ day^−1^) measured at each site during the month of May 2023 (Fort Lauderdale *n* = 5, Dania Beach *n* = 10, South Canyon *n* = 10, Emerald Reef *n* = 10, Carysfort Reef *n* = 10, Conch Reef *n* = 4). **e** Logistic regression for the relationship between the probability of encountering a juvenile coral and LSAT sediment depth (mm; *n* = 150 quadrats). The solid line represents the best-fit generalized linear mixed-effects model with a logit link function, and the shaded area represents the 95% confidence interval of the curve.

During May 2023, LSAT sediment depth (K-W Chi-squared = 42.42, df = 5, *p* < 0.0001, Fig. S2), sediment grain size (permuted *F*_5,144_ = 88.53, *R*^2^ = 0.75, *p* = 0.0001; Figs. 5c and S3), sedimentation rates (*F*_5,43_ = 7.34, *p* < 0.0001; Fig. 5d), and LSAT benthic cover (*F*_5,144_ = 7.79, *p* < 0.0001; Fig. S4) exhibited significant variability among our six field sites located across southeast Florida and the upper Florida Keys (Fig. 5a, b and Tables S5 and S6). Median LSAT sediment depth was highest at Conch Reef, our southernmost site (median = 3.0, MAD = ±1.5 mm), and lowest at South Canyon and Fort Lauderdale, two of our northernmost sites (Fort Lauderdale = 1.0 ± 0.0 mm, South Canyon = 1.0 ± 1.5 mm; Tables S6 and S7). Overall, the sedimentation rates of our three southernmost sites in the upper Florida Keys tended to be greater than those of our three northernmost sites in southeast Florida (Fig. 5d and Tables S6 and S8). Conch Reef exhibited the greatest sedimentation rate (mean ± SE; 9.7 ± 1.8 g m^−2^ day^−1^), which was more than 3-fold the sedimentation rate measured at Dania Beach (2.9 ± 0.4 g m^−2^ day^−1^). LSAT was the predominant benthic space-holder at all of our field sites. The average percent cover of LSAT ranged from a high of 58.5 ± 4.1% recorded at Conch Reef to a low of 34.2 ± 3.6% recorded at South Canyon (Tables S6 and S9).

When considering the relationship between LSAT parameters (percent benthic cover, turf length, and sediment depth) and coral recruits, the most parsimonious model was a GLMM with LSAT sediment depth as the sole, significant, negative predictor of coral recruit presence (fixed effect of LSAT sediment depth: estimate = −0.56, SE = 0.21, *z* = −2.60, *p* < 0.01; Fig. 5e). We recorded the highest mean density of coral recruits at Emerald Reef (8.3 individuals m^−2^) and the lowest mean density of recruits at Fort Lauderdale (2.6 individuals m^−2^). Conch Reef, the site with the highest recorded median LSAT sediment depth, was also the site with the second lowest mean density of recruits (3.2 individuals m^−2^; Table S10). *Siderastrea* spp. was the most abundant coral taxon in our recruit surveys, comprising 66% of all recruits encountered across all sites (mean *Siderastrea* spp. recruit density across all sites: 3.7 individuals m^−2^). *Porites* spp. was the second most abundant taxon in our surveys, comprising 17% of all recruits encountered across all sites (mean *Porites* spp. recruit density across sites: 1.0 individuals m^−2^, Table S10).

## Discussion

This study thoroughly assessed the influence of sediment burial on larval settlement and recruit survivorship across multiple early life stages in a suite of Caribbean coral species. We demonstrate that sediment loads of 150 to 300 mg cm^−2^, values typically recorded on contemporary reefs across the globe^88^, can severely suppress larval settlement and rapidly kill coral recruits. Moreover, the strong adverse effects of sediment burial were consistent across all species and life histories tested. For coral larvae, the burial of substrata by sediment was the main driver of decreased settlement, evidenced by the strong response of larvae to the burial of settlement substrata across all species. For example, sediment loads of just 150 mg cm^−2^, equivalent to a 2 mm-deep layer in this study, decreased larval settlement probability to less than 5%, and a sediment load of 300 mg cm^−2^ inhibited larval settlement for all but one species tested: *D. labyrinthiformis*. Similarly, the burial of coral recruits spanning ages 1–18 months old under 300 mg cm^−2^, equivalent to a 4 mm-deep layer in this study, increased their mortality risk by 3- to 25-fold. Our experimental results were supported by our field observations, which highlight the dominance of LSAT on Florida’s reefs and their contribution to the increasing scarcity of juvenile corals^27,74^. Together, our findings highlight that the consequences of sedimentation are pervasive for at least the first 18 months of age for the species tested and have the potential to severely diminish the capacity for coral populations to recover.

Sedimentation is widely recognized as a ubiquitous threat to coral reefs globally^49,82,89^. The lethal and sublethal effects of sedimentation on corals are well documented and can vary widely among species, sediment load, composition, and burial duration (reviewed in refs. ^14,46^). However, beyond impacting existing coral populations, deposited sediments on coral reefs also have the potential to undermine the ability of coral populations to recover after a disturbance. One pathway by which sedimentation can reduce population recovery is by suppressing larval settlement^73,82^. Here, we found that a sediment load of 150 mg cm^−2^ (2 mm deep) was sufficient to decrease the probability of larval settlement by at least 75% for all five Caribbean species tested. Furthermore, a sediment load of 300 mg cm^−2^ (4 mm deep) suppressed larval settlement entirely for all species except *D. labyrinthiformis* (Fig. 1). The relatively low sediment load required to induce severe declines or inhibit settlement by key reef-building species supports the hypothesis that sedimentation can serve as a major roadblock to the recovery of coral populations in the Caribbean. These findings are particularly alarming given that the sediment loads used in our experiments (150–300 mg cm^−2^) reflect those reported on coral reefs across the globe^88^ and that we observed in our surveys of Florida’s LSAT assemblages (Figs. S5 and S6).

At our sites, LSAT assemblages with sediment layers 3 mm and 6 mm deep contained sediment loads of approximately 150 mg cm^−2^ and 320 mg cm^−2^, respectively (Fig. S5). Moreover, our surveys and others (e.g., refs. ^27^, ^90^) across South Florida report LSAT as the most dominant benthic space-holder, reducing suitable recruitment habitat on Florida’s reefs. It is evident that a positive feedback loop between sedimentary and algal turf dynamics contributes to the prevalence and stability of this ecological state (ref. ^91^; Fig. S6). The drastic loss of coral cover, coupled with high bioerosion rates^92^, local dredging activities^3^, and hurricanes^93^ could be increasing the sediment load on Florida’s reefs. However, the individual contributions of these stressors to the feedback loop are yet to be addressed. Furthermore, algal turf productivity and biomass vary seasonally, peaking in the summer and decreasing during the winter^94–96^. Therefore, LSAT characteristics at our sites likely exhibit some seasonal variability (we conducted our surveys during May 2023^90,97^) Future studies should address the spatiotemporal stability of LSAT and the interplay between algal turf productivity and sediment dynamics to better understand the mechanisms that underpin LSAT formation and persistence.

Our field surveys of coral recruit abundance further corroborate our lab-based observations of declining settlement with increasing sediment loads: the probability of encountering a juvenile coral (≤4 cm diameter) within a quadrat decreased to near zero where the LSAT sediment layer was 4–6 mm thick (Fig. 5e). Similar to our findings, work on Pacific coral species has found strong effects of sedimentation on coral settlement. For example, settlement of *Pocillopora damicornis* larvae was completely inhibited when more than 95% of the substratum was covered by fine sediment (sediment load 530 mg cm^−2^; ref. ^98^). Similarly, settlement by *Acropora millepora* larvae was reduced by 90% on substrata with sediment loads of 100 mg cm^−2^ (ref. ^73^), and settlement was inhibited on substrata buried under higher sediment loads (217–650 mg cm^−2^; ref. ^99^). Given the comparable sediment loads in places like the Great Barrier Reef^88,100^, there is mounting evidence that sediment-driven reductions in larval settlement may be a widespread phenomenon on coral reefs across the globe.

While the primary mechanisms by which sedimentation reduces settlement remain unresolved, masking of settlement cues, a negative tactile response, and attachment failure are among the most prominent hypotheses^82^. Previous studies assessing the influence of sedimentation on coral larval settlement have focused on Indo-Pacific species from the Acroporidae^73,75–77,99^ and Pocilloporidae families^98,101,102^. The majority of these experiments used fine sand (<125 µm) or silt (≤63 µm) in their settlement assays because allochthonous fine sediment and silt—typically imported onto reefs by terrestrial runoff and generated in large quantities during dredging operations—are generally considered to have more detrimental impacts on corals compared to autochthonous coarse sediment^46,78^. However, the coarse sand (predominant grain size ≥500 µm) used in our assays (Fig. S7) still strongly suppressed coral settlement and survival. Our field data support this trend: southern sites such as Conch Reef, with high coarse-grain proportions (60–67% ≥250 µm; Table S11), also had the highest LSAT sediment depths and lowest density of coral recruits. These findings are concerning for the recovery of coral reefs impacted by sedimentation, and may underestimate the additional impacts of finer sediment^3^. Furthermore, in situ reef sediment may harbor harmful pathogens that could reduce settlement or increase the likelihood of recruit mortality that would not be present in oven-dried, sterile sediment from a laboratory setting^103^. Consequently, our use of coarse sand grains devoid of biological communities suggests that our results may be conservative estimates of the potential impacts of sedimentation on coral settlement^79^. Future research quantifying the influence of different sediment grain sizes and sources on coral larval settlement is imperative to better predict the effects of sedimentation on coral populations.

Lab-based studies that accurately emulate natural coral-reef environments are difficult to execute but are important for understanding how abiotic factors, such as water flow dynamics and the complex biogeochemical processes that characterize sediment matrices, influence larval settlement. Indeed, microbial assemblages within the sedimentary matrix and the hydrodynamics of the benthos play important roles in the remineralization of organic nutrients, the decomposition of detritus, and larval settlement cues, which can influence the survival and distribution of benthic macroorganisms^104–106^. The chambers where we conducted our settlement assays intentionally lacked water flow to allow us to establish and maintain our sediment burial depth treatments, and the sediments were sterilized by heat (oven-dried) to prevent rapid declines in water quality. Additionally, sterilizing the sediments allowed us to assess the sole contribution of the physical presence of sediments, isolated from any potential biogeochemical co-factors. Consequently, our results provide a baseline for the response of larval settlement to the physical effects of sediment deposition; however, future studies that can incorporate water flow or use ‘live’ sediments are important next steps to understand how these factors influence settlement rates.

Beyond settlement, sedimentation could also impede coral recovery by impacting newly settled coral recruits, yet little information exists regarding the effects of sedimentation on this highly sensitive life history stage^77,82^. Here, we found that burial under 4 mm of sediment severely reduced the survival probability of all species and age groups tested except for 1-month-old *C. natans* recruits, which still experienced a notable reduction (68%) in probability of survival. The species that showed the highest reductions in survival when exposed to sediment burial were *Acropora cervicornis* and *Orbicella faveolata*, two historically dominant reef builders that are currently listed as “threatened” under the U.S. Endangered Species Act^107,108^. The burial of 3-month-old *A. cervicornis* recruits under 2 mm of sediment for 10 days decreased survival probability to 14%, and burial under 4 mm of sediment for 10 days further decreased it to zero. *O. faveolata* fragments representative of 6-, 12-, and 18-month-old recruits were 12x, 13x, and 16x, more likely to suffer mortality when buried under 4 mm for 10 days compared to the controls, respectively (Fig. 3). Moreover, these larger corals were also susceptible to burial under 2 mm of sediment, with mortality risk increasing 12x for 6-month (1 cm^2^) and 18-month (4 cm^2^) sized coral fragments.

Previous studies on Indo-Pacific corals have also reported that the first month of a coral’s life is the most sensitive life stage. Moeller et al.^77^ assessed the survival of recruits exposed to sedimentation and found that a sedimentation rate of 33 mg cm^−2^ d^−1^ caused up to 80% mortality of *Acropora hyacinthus* and *Lepastrea purpurea* recruits across 42 days (total sediment load = 1400 mg cm^−2^), but the majority of recruits were already dead within the first 15 days (15-day sediment load = 495 mg cm^−2^). Since little to no mortality was attributable to sedimentation in recruits older than 4 weeks, they concluded that the most sensitive age classes to sedimentation were recruits younger than 4 weeks old^77^. In stark contrast to these findings, we report that burial under 300 mg cm^−2^ of sediment is detrimental to the survival of recruits up to 18 months old. The contrast among these studies is likely a consequence of different sediment delivery rates. Moeller et al.^77^ gradually introduced sediment into the system every 3 days and replaced it with new sediment over these 3-day periods across 42 days, which is more representative of chronic sedimentation stress such as terrestrial runoff. By contrast, this study assessed the impact of large, acute sedimentation stress that rapidly buries corals for up to 10 days such as dredging events^3^. Therefore, our results indicate that the rapid, acute burial of coral recruits is more detrimental to recruit survival than the gradual deposition and resuspension of sediments from chronic sedimentation stressors.

The reduction in light levels and the high rates of microbial respiration within sedimentary matrices are some of the major biogeochemical mechanisms that contribute to coral stress and subsequent mortality^59,109^. Previous studies have shown that the mineralization of organic matter, driven by microbial respiration within reef sediments^13^, can induce hypoxic or anoxic conditions within the first 2–4 mm of the sedimentary matrix^109,110^—the depth range tested in this study—and induce coral mortality. Furthermore, nutrient-rich sediments, often introduced from terrestrial systems, tend to promote higher respiration rates and deplete oxygen faster and at shallower depths than nutrient-poor reef sediments^59^. Although these biogeochemical mechanisms are known to be major contributors to coral mortality, few studies have quantified their effects within this context. Our study, for instance, did not measure oxygen concentration within sediment pore water because it was out of our scope—we were primarily interested in assessing how different sediment loads impact larval settlement rates and overall coral survivorship for multiple coral species. Nevertheless, future studies should incorporate biogeochemical parameters to determine how interactions among physical, chemical, and biological factors impact coral survivorship.

The only other study we are aware of that has tested the effects of sedimentation on recruits of Caribbean coral species reported that the probability of survival for 5-month-old recruits of the stress-tolerant species *Porites astreoides* was reduced to zero when exposed to sediment loads as low as 60 mg cm^−2^ within just 4 days. Moreover, sediment loads of 30 mg cm^−2^ were enough to achieve this same effect under an elevated temperature regime of 30 °C (ref. ^78^). Similar work using the Pacific coral species *Acropora millepora* found that recruits exposed to elevated temperatures (1.2 °C above historic daily mean) were 2x more likely to succumb to sedimentation-induced mortality compared to recruits exposed to ambient temperatures^16^. Such findings underscore how extremely low sedimentation levels can exacerbate pervasive stressors like thermal stress that impact corals across the globe^6^.

Our study documents the severe effects that small to moderate sediment loads can have on the first 18 months of six different Caribbean coral species. Our experimental results indicate that sediment loads of 300 mg cm^−2^—equivalent to a sediment layer 4 mm deep—can inhibit larval settlement for all species tested and that burial under such sediment loads for 10 days can kill coral recruits up to 4 cm^2^ in size (approximately 18 months old). The sediment loads tested here represent common levels of sediment loading on reefs in Florida^3,111^ and places like the Great Barrier Reef^69^, suggesting that many reefs may have already exceeded thresholds at which sediments may compromise coral population recovery. Our field observations further corroborate our experimental observations, showing that recruit abundance on Florida’s reefs sharply declines in the presence of LSAT sediment layers ≥4 mm deep. This study highlights the serious consequences that continued sedimentation—from chronic disturbances such as reef erosion and runoff, and acute disturbances such as dredging—can have on the recovery of Caribbean coral populations by suppressing larval settlement and increasing the mortality risk of established recruits. Importantly, these findings suggest that local management actions to decrease sediment loads reaching coral reefs can minimize a major bottleneck to coral recruitment. As such, it is imperative to identify the major processes producing allochthonous sediment and importing autochthonous sediments on reefs to target them and effectively mitigate their future impacts. Minimizing the impact of future coastal development projects, such as port dredging and beach renourishment, and improving habitat quality on coral reefs, through thoughtful restoration efforts and the mitigation of local stressors, are key steps to reduce factors impacting the survival of early life history stages and promote the recovery of coral populations.

## Methods

To address our questions, we coupled aquaria-based experiments using larvae and coral recruits generated from 2023 coral spawning collections with field surveys conducted during May 2023 on coral reefs in Florida, USA. In this study, we define coral recruits as any settled and metamorphosed coral (from the planula larval stage to the polyp stage) of 1–18 months of age. When the age of the coral colony is unknown, we define recruits as coral colonies with a maximum diameter ≤4 cm. We conducted a series of aquaria-based experiments to address two main goals: (1) understand how burial of the substratum influences coral settlement, and (2) understand the effect of sediment burial depth and duration on recruit survivorship across different life history stages. Multiple species and life history stages were used to tease apart species-specific responses to sedimentation and determine how these responses may vary as a function of coral age. The main objectives of the field surveys were to assess the variation of benthic assemblages and sedimentation rates on coral reefs in southeast Florida across a north-to-south gradient and identify any potential relationships between ecological and sedimentological processes. All animals were collected, reared, and experimented on under the permission of appropriate federal and state authorities: the National Oceanic and Atmospheric Administration, the Florida Fish and Wildlife Commission, and the Florida Department of Environmental Protection. All work was conducted under permits FKNMS-2018-163-A4, FKNMS-2023-021, and SAL-23-2526-SRP. We have complied with all relevant ethical regulations for animal use.

### Coral settlement assays

We conducted settlement rate assays in August 2023, September 2023, and April 2024 using larvae produced from coral spawning during the summer (2023) and spring (2024) spawning windows^112^ (Table S12). Each replicate trial for the settlement assays was performed in an individual glass chamber (118 mL capacity, 6.5 × 8 cm, diam. × H) that was placed in a water bath (35 L capacity, 62 × 43 × 17 cm; L × W × H) in a block design. Experimental treatments were assigned haphazardly across each water bath, ensuring that each tank had its own, unique haphazard pattern. Water baths were maintained at 28.00 ± 0.03 °C (Fig. S8) using digital temperature controllers (Finnex HC-810M, ISK Merchandising Inc., USA) and 300 W titanium heaters (Finnex TH-303005, ISK Merchandising Inc., USA). Water was consistently circulated throughout each water bath via a powerhead (Eco Wave EW-10 Wave Pump, Sea Side Aquatics, LLC, Anaheim, CA, USA). Water temperature was recorded using temperature data loggers (HOBO Pendant MX Temperature/Light Data Logger, Onset Corporation, Bourne, MA, USA) that recorded every 5 min. Each water bath contained 15–30 individual chambers, such that a single run (*n* = 45 chambers) was spread across two water baths.

To create a gradient of sediment presence and depth for the burial of settlement substrata, we added enough sediment to 3.8 × 3.8 × 0.5 cm (L × W × H) ceramic tiles in seawater to create a ‘sprinkle’ on the tile (a thin veneer of sediment covering <50% of the tile surface), a layer of sediment 2 mm deep covering 100% of the tile surface, and a layer of sediment 4 mm deep covering 100% of the tile surface (*n* = 10 per treatment). Once enough sediment was added to achieve these treatments (verified by measuring the sediment layers with a ruler), the sediment was carefully removed, dried, and weighed to standardize the amount of sediment added to each replicate for each treatment. Sediment treatments consisted of 0.15 g sediment (equivalent to a sediment load of 10 mg cm^−2^), which provided a ‘sprinkle’ of sediment across the settlement substratum, 2.15 g of sediment (equivalent to a sediment load of 149 mg cm^−2^), which covered the settlement substratum with 2 mm of sediment, or 4.30 g of sediment (equivalent to a sediment load of 298 mg cm^−2^), which covered the substratum with 4 mm of sediment. To quantify sediment loads in mg cm^−2^, we calculated the surface area of the ceramic tiles that we used as substratum for larvae to settle on (3.8 × 3.8 cm = 14.4 cm^2^) and divided it by the weight of sediment for each treatment used to determine the sediment load in g cm^−2^ (example for 2 mm treatment = 2.15 g/14.4 cm^2^ = 0.1489 g cm^−2^). We then multiplied the sediment load in g cm^−2^ by 1000 to convert it to mg cm^−2^, which are the units commonly used in the literature^73,88,98^. These sediment depth treatments were selected to correlate with those measured on LSAT across our field sites (Fig. S5). Additionally, we wanted to test the impacts that minimal amounts of sediment could have on early life-history stages in the context of past and planned dredging activities in South Florida (sediment layers 1–5 mm deep ref. ^113^). Since we were working with coarse sediment (Fig. S7), the shallowest sediment layer that we could achieve while covering the entire substratum was 2 mm, and therefore, this depth was set as our lowest threshold.

We also tested the effect of sediment source by using sediments collected at approximately 5–8 m depth at two sites near Port Everglades (PEV) and a reef site offshore of Key Largo (KL, Fig. 5b). Each chamber was assigned one of eight experimental sediment treatments or a control, which received no sediment addition. Altogether, each settlement assay consisted of nine different treatments: (1) +2.15 g of PEV sediment present, (2) +4.30 g of PEV sediment present, (3) +0.15 g of PEV sediment burial, (4) +2.15 g of PEV sediment burial, (5) +4.30 g of PEV sediment burial, (6) +2.15 g of KL sediment present, (7) +0.15 g of KL sediment burial, (8) +2.15 g of KL sediment burial, as well as (9) controls where no sediment was present in the chamber (Fig. S9). These treatments were designed to explicitly test the effect of the presence of sediments in the chamber but not physically covering the settlement substratum (“Sediment Present”) and the effect of sediments physically covering the settlement substratum (“Sediment Burial”; Fig. S9).

All sediments were collected via SCUBA in July of 2023. Sediments were collected using a glass scoop and placed into 2-gallon Teflon bags that were sealed underwater. Upon surfacing, bags were drained of as much seawater as possible, sealed, and placed on ice for transport to the lab, where they were frozen at −20 °C until they were dried. All sediments were dried at 60 °C until they reached a constant weight, at which point they were sieved to remove any material >5 mm diameter and were placed into teflon bags and sealed until use in experiments. The sediment used in this study was predominantly of a grain size ≥500 µm (KL = 82%, PEV = 63%; Fig. S7), which is classified as medium to coarse sand by Wentworth^114^. We ran a binomial generalized linear mixed-effects model (GLMM) to determine whether there were differences in larval settlement success among sediment sources (KL vs. PEV), and there was no observed difference in settlement success between the source treatments (coefficient for “source” = −0.163, SE = 0.208, *p* = 0.43; Fig. S10). Therefore, we combined these trials in our final analyses to assess the general response of larval settlement to sediments.

Each chamber received a single 3.8 × 3.8 × 0.5 cm (L × W × H) ceramic tile (Boston Aquafarms, Boston, MA, USA). All tiles were seasoned in the field for 2 months prior to the start of the experiment at the same site where we collected Key Largo sediment (Fig. 5b). Once a chamber received a tile, it was filled with 100 ml of filtered (20 µm) and UV-sterilized seawater originating from Bear Cut, Miami, FL, USA. This water source and filtration level are routinely used for larval settlement and recruit rearing without issue^112,115,116^. Prior to adding sediments to the chambers, the top surface of each tile received a standardized amount of pulverized crustose-coralline algae (CCA) to encourage settlement. CCA was harvested from a single aquarium using a razor blade and thus was of consistent origin and identity across all treatments and replicates. Dried sediments were pre-weighed to standardize the amount of sediment added to each settlement chamber for each treatment. For the “Sediment Present” treatments, sediments were added to the bottom of the chamber surrounding the tile (i.e., no sediments present on top of the settlement substratum), while for the “Sediment Burial” treatments, the sediments were added directly on top of the settlement substratum (Fig. S9).

After establishing sediment treatments and waiting ~1 h for the sediment to settle, coral larvae were placed into each settlement chamber (*n* = 20 larvae/chamber for *Orbicella faveolata* and *Diploria labyrinthiformis*, totaling 2100 larvae per species; *n* = 15 larvae/chamber for *Colpophyllia natans*, *Psuedodiploria clivosa*, and *P. strigosa*, totaling 1575 larvae per species). Five replicate chambers were established per treatment, run, and species. We conducted at least 3 runs (9 treatments × 5 replicates per treatment = 45 chambers per run) for each species. Sample size was limited by larvae available for experimentation purposes, personnel availability and processing time. Larvae were deemed ready for use in experiments when we observed the onset of settlement within the main larvae holding tanks. Larvae were removed from their main holding tank via pipette and placed into individual 0.2 ml wells for transfer to experimental settlement chambers. Once larvae were introduced, chambers were sealed to prevent evaporation and changes in salinity. We quantified larval settlement rates in each treatment after 72 h by closely inspecting each tile using a dissecting microscope and fluorescent lights (NIGHTSEA Full System with Royal Blue Illumination, Electron Microscopy Sciences, PA, USA). For sediment burial treatments, we first inspected the sediment surface for any coral settlers. Then, while looking under the microscope, we gently pipetted the sediment off the tile to reveal the settlement substratum below to look for any coral settlers. Settlers were recorded as either “settled”, meaning that they had metamorphosed into a flat, disk-like shape to adhere to the substratum, or “attached”, whereby they had firmly attached themselves to the substratum but had not yet metamorphosed (Fig. S11). The number of larvae settled and attached was recorded for the upward-facing surface of each settlement substratum, the tile sides, and the bottom. Only settlers recorded as settled or attached on the upward-facing surface of settlement substrata were included in our analyses.

Sediment is known to decrease water quality, especially in closed systems like our settlement chambers^73,79^. To assess the influence of our sediment treatments on water quality, we conducted another run of settlement assays as reported above, but without adding coral larvae. Since no larvae were included, the run included all treatments except the ones that had sediment burying the settlement tiles; therefore, our water quality assay contained five replicates with sediment added around the tiles for the following treatments: control (no sediment added), ‘sprinkle’ of PEV sediment, ‘sprinkle’ of KL sediment, 2 mm of PEV sediment, 2 mm of KL sediment, and 4 mm of PEV sediment. Water quality parameters were assessed 72 h after the addition of sediments using a Hach DR1900 portable spectrophotometer (Hach Company, Loveland, CO, USA). The parameters tested were salinity (ppt), pH, ammonia (NH_3_), nitrite (NO_2_^−^), nitrate (NO_3_^−^), and sulfide (S_2_^−^). Salinity and pH were measured using the Hach HQ2200 portable multi-meter (salinity detection limit = 0–42 ppt; pH detection limit = 0–14), ammonia was measured with the Ammonia Salicylate Method 8155 using Ammonia Salicylate and Ammonia Cyanurate powders (detection limit = 0.01–0.50 mg/L NH₃^-^N), nitrite was measured with the USEPA Diazotization Method 8507 using NitriVer 3 Reagent powder (detection limit = 0.002–0.300 mg/L NO_2_^−^ -N), nitrate was measured with the Cadmium Reduction Method 8039 using NitraVer 5 Nitrate Reagent powder (detection limit = 0.3–30.0 mg/L NO_3_^−^ N), and sulfide was measured with the Sulfide Methylene Blue Method 8131 (detection limit = 5–800 µg/L S²⁻).

### Coral recruit sediment burial assays

We tested the influence of sediment burial on the survival of coral recruits across five Caribbean coral species: *Acropora cervicornis*, *C. natans*, *D. labyrinthiformis, O. faveolata*, and *P. strigosa*. To test how the effects of sediment burial may change with coral age, we used recruits of different ages, including 1-month-old recruits (*C. natans*, *O. faveolata*, and *P. strigosa*), 3-month-old recruits (*A. cervicornis*, *C. natans*, *O. faveolata*, and *P. strigosa*), and 6-month-old recruits (*D. labyrinthiformis* only). In addition, we tested the effect of sediment burial on fragments of *O. faveolata* approximating the size of 6-month, 12-month, and 18-month-old recruits. This was accomplished by fragmenting established *O. faveolata* colonies into size classes representative of each age group: 1 × 1 cm (1 cm^2^) for 6-month-old recruits, 1.5 × 1.5 cm (2.3 cm^2^) for 12-month-old recruits, and 2 × 2 cm (4 cm^2^) for 18-month-old recruits. The species included at each life history stage were based on recruit availability. For these experiments, we exposed coral recruits to three treatments: a control (no sediment present), burial under 2 mm of sediment, or burial under 4 mm of sediment (Figs. 2 and 3). To test how burial duration influences survival, recruits were exposed to sediment burial treatments for 1, 2, 3, 5, 7, or 10 days.

We used a block design to conduct the experiments in 20-gallon tanks where we deployed 6 × 3 grids of PVC chambers (5 × 5 cm each), whereby each chamber was designed to house a single tile, with each row containing the three treatments (control, 2 mm, 4 mm) per day (6 days for 3 treatments). We had four tanks per species per age group (24 tanks total, with 432 recruits across all tanks), each consisting of one replicate per species per age group. The number of replicates was determined by the availability of coral recruits for each species and size class to ensure equal sample sizes across life stages and species. On day 0, we counted, photographed, and mapped the location of all recruits within each tile using a dissecting microscope. The tiles were then placed into their respective chambers, and sediment was added to each one based on their assigned experimental treatment (2 mm and 4 mm) using a pipette. Experimental treatments were assigned haphazardly across each grid, ensuring that each tank had its own, unique haphazard pattern. Burial depth was based on the distance from the substrata upon which a coral was growing to the surface of the sediments. To achieve consistent sediment burial depths across replicates, the same volume of sediment was added to each chamber, depending on the treatment, and was applied uniformly across the chamber. At each timepoint, sediment was removed from the respective tiles using a siphon, and the number of living and dead coral recruits was counted and photographed (Fig. S12).

Coral recruits used for the sediment burial experiments were reared from larvae settled from the summer of 2023 coral spawning windows (August and September) and spring of 2023 (April and May; *D. labyrinthiformis* only); coral larvae source details are provided in Table S12. Using a diamond-coated bandsaw (C-40 Diamond Band Saw; Gryphon Corporation, Sylmar, CA, USA), we fragmented an *O. faveolata* colony that was born during the 2019 spawning period (approx. 5 years old). The recruits reared and used for the burial experiments were independent from those that survived the settlement assay experiments. Larvae were settled onto 3.8 × 3.8 × 0.5 cm ceramic tiles, and the resulting recruits were reared in aquaria at NOAA’s Coral Reef Assessment and Research Lab (CoRAL) facility in Miami, FL. Recruit sediment burial experiments were also conducted in aquaria at NOAA’s CoRAL facility. These aquaria consist of 20-gallon aquaria with 20-gallon sumps fed filtered (20-micron) and UV-sterilized seawater with a turnover rate of ~6x per day. Lighting was consistent for each cohort via Radion XR30 G6 Pro LED aquarium lights (EcoTech Marine, PA, USA), and the temperature was kept consistent at ~28 °C using 300 W titanium heaters (Finnex TH-303005 titanium heater, ISK Merchandising Inc. USA) controlled by an Apex Neptune controller system (Figs. S13–S15). In the trials for the 3-month-old *C. natans* and *O. faveolata*, there was a decrease in the average seawater temperature of ~1.5 °C within days 2 and 3 of the experiment. However, this temperature change is within typical observed temperature fluctuations that corals experience on Florida reefs and was not associated with an increase in mortality during our experiment.

### Study species

Our species selection was based on the availability of larvae spawned in the summer of 2023 and spring of 2024, coral spawning activities, species’ conservation status, ecological importance, and susceptibility to current stressors. For instance, *A. cervicornis* and *O. faveolata* are two species listed as “threatened” under the U.S. Endangered Species Act^107,108^ and are key framework builders on Caribbean reefs. Their populations have steeply declined across the Caribbean over the last few decades^9,117–119^. Similarly, *C. natans, D. labyrinthiformis*, and *P. strigosa* are massive, framework-building coral species highly susceptible to stony coral tissue loss disease (SCTLD), which has decimated their populations across the Caribbean over the last decade^85,120–122^.

### Field surveys

During May, 2023, we sampled six shallow (6–8 m deep) reef sites along the Florida reef tract, from Fort Lauderdale in Broward County, USA, to Key Largo in the upper Florida Keys, USA (Fig. 5b). Our sites include Fort Lauderdale (Broward County; surveyed on May 10, 2023), Dania Beach (Broward County; surveyed on May 9, 2023), South Canyon (Miami Beach; surveyed on May 11, 2023), Emerald Reef (Key Biscayne; surveyed on May 16, 2023), Carysfort Reef (Key Largo; surveyed on May 24, 2023), and Conch Reef (Key Largo; surveyed on May 25, 2023; Table S5). These reefs are characterized as offshore shelf-edge reefs^123^, and we focused on sampling the shallow fore-reef habitat at each site (5–7 m).

At each site, we haphazardly outlined a permanent 25 m transect oriented from north to south using the surveyor’s tape. We hammered metal nails at every 1-meter interval to mark all 25 plots in a site. We used a 25 × 25 cm quadrat to characterize the in-situ benthic community composition at the lowest taxonomic resolution possible. Whenever we found LSAT within the 25 × 25 cm plot, we recorded the sediment depth at five randomly selected locations. Each plot was photographed for quality control (taxa identification and percent cover estimation). Within each 25 × 25 cm quadrat, we also identified every juvenile coral (≤4 cm diameter) to the genus level and measured their maximum diameter (cm). The number of coral juveniles was divided by the area of the plot (0.0625 m^2^) to estimate the density of juvenile corals (individuals m^−2^).

We characterized LSAT within a 5 × 5 cm mini-plot (*n* = 25 per site) located near the corresponding 25 × 25 fixed plot. We measured sediment depth (mm) within each mini-plot by placing a calibrated ruler into the LSAT. Sediment depth was the average of three measurements taken around the center of the mini-plot. Then, we extracted all sediments from the LSATs within each mini-plot using 60 mL syringes. Extracted sediments were carefully placed into a bag underwater for future processing. Once the sediment was extracted from each subplot and the turf algae were fully exposed, we measured turf height within the mini-plot. Turf height was calculated as the average of three measurements taken around the center of the mini-plot.

To estimate sedimentation rates, we deployed ten sediment traps at each site. Each sediment trap consisted of a PVC pipe (internal diameter: 5 cm, height: 43 cm) with a set of two zip-lock bags cable tied over the bottom end, serving as the reservoir. We secured each trap to the substratum via cable ties attached to two nails hammered into the reef framework. The traps were deployed on the day of in situ surveys and were retrieved from the field after approximately 14 days.

Upon collection from the field, sediment samples were placed in a large mesh bag at ambient temperature and transported to the Coastal Conservation and Restoration Laboratory (CCRL) at Florida International University, where they were immediately placed in a freezer at −15 °C. The sediment samples from the LSAT quadrats and the sediment traps were then rinsed in freshwater and oven-dried to a constant weight at 70 °C for 12 h. Once all samples had been rinsed and dried, they were separated by grain size using sieves with mesh sizes of 500 µm, 250 µm, 125 µm, and <125 µm, and the yield of each size class was weighed (g).

### Statistics and reproducibility

All statistical analyses were run in R version 4.5.1 (ref. ^124^). We conducted one-way analyses of variance (ANOVAs) to test the variation of mean sedimentation rates (g day^−1^ m^−2^; Fort Lauderdale *n* = 5, Dania Beach *n* = 10, South Canyon *n* = 10, Emerald Reef *n* = 10, Carysfort Reef *n* = 10, Conch Reef *n* = 4) and LSAT benthic cover (%) across sites (*n* = 25 quadrats per site), followed by Tukey HSD multiple comparison tests. The sedimentation data were transformed using natural log to conform to the assumptions of normality and homoscedasticity. The LSAT sediment depth (mm) data did not meet the assumptions for ANOVA, and data transformations were unsuccessful. Therefore, we conducted a Kruskal-Wallis test followed by a Dunn’s post hoc test to examine the variation in medians across sites. Using the “lme4” package^125^, we conducted a generalized linear mixed-effects model (GLMM) with a logit link function to test the relationship between LSAT depth and LSAT cover with the presence of coral recruits. Since the abundance of coral recruits was 0 in many of the surveyed quadrats (average recruit abundance across sites = 0.5 individuals per 625 m^2^; *n* = 150 quadrats), we transformed the abundance data into binary form to account for the zero-inflated dataset. A value of 0 indicated no recruits were recorded, and a value of 1 indicated that at least one recruit was recorded per quadrat. LSAT depth and cover were specified as the predictor variables, the presence of coral recruits was specified as a fixed response variable, and the site was specified as a random effect.

For the larval settlement experiments, we conducted two binomial GLMMs with logit link functions: one to determine the influence of sediment presence around the substratum on the settlement success of coral larvae among multiple species, and a second one to determine the influence of burial of the substratum on the settlement success of coral larvae among multiple species. For each model, coral species and sediment load (mg cm^−2^) were specified as fixed predictor variables and the replicates nested within treatments were specified as random effects. For the response variable, we used the proportion of larvae (0.0–1.0) that settled onto each replicate (*n* = 60 per species for burial assays, *n* = 45 per species for sediment presence assays).

For the water quality assays, using the “vegan” R package^126^, we conducted a permutational analysis of variance (PERMANOVA) with the Euclidean similarity index and 9999 permutations to determine the difference in water quality parameters among treatments after 72 h. To account for the different magnitudes and ranges of the units of all parameters, the data were transformed by taking the natural log of the standardized *z*-scores of each variable. We then used a similarity percentage (SIMPER) test to identify the main variables driving the differences among treatments; to account for multiple comparisons, we ran 9999 permutations to generate a null distribution to compare with the test statistics of our data. Using the “BiodiversityR” package^116^, we also used a non-metric multidimensional scaling (nMDS) plot to visualize the degree of separation among groups and determine the main sedimentary components driving the separation among treatments.

Using the “survival” package in R^127^, we plotted Kaplan-Meier curves to assess changes in survival probability for each sediment treatment (*n* = 24 per species, life stage, and treatment). The Log-Rank test was used to test whether the survival curves among treatments differed from those of the controls. Additionally, a Cox Proportional-Hazards model was run for each species and life stage combination to determine the hazard ratio of each treatment relative to the control trials. We used the Schoenfeld individual test to test the proportional hazards assumption for the Cox-PH models. All Schoenfeld residuals exhibited a random pattern through time, indicating that the proportional hazards assumption was met. To test for outliers, we used the “survminer” package^128^ in R to plot the dfbeta residuals against the regression coefficients to assess their influence on the model. Finally, we used the hazard ratios estimated from the Cox proportional-hazards models to simulate the response of juvenile coral assemblages to hypothetical sedimentation events and assess how variations in the duration of burial and the proportion of recruits buried reduce the total recruit stock within an affected area through time (see Supplementary Methods).

### Reporting summary

Further information on research design is available in the Nature Portfolio Reporting Summary linked to this article.

## Supplementary information

Supplementary Information

Reporting Summary

Transparent Peer Review file

## Data Availability

All data and R code are available at the following Zenodo repository^129^.

## Code availability

All code used for analysis is stored with the raw data at the following Zenodo repository^129^.
