# Supplementary material for: Sediment burial reduces coral larval settlement, recruit survivorship, and inhibits the recovery of coral populations
Source: Commun Biol. 2026 Jun 17;9:1284. doi: 10.1038/s42003-026-10370-1 (PMC13635240; doi:10.1038/s42003-026-10370-1)
Supplement: Supplementary file 1 [file 42003_2026_10370_MOESM1_ESM.pdf]

Supplementary Material: Sedimentation reduces coral larval settlement, recruit survivorship, and inhibits the recovery of coral populations

Authors: Victor Rodriguez-Ruano[1,2], Alain Duran[3], Allan J Bright[1,2], Kathryn Grazioso[1,2], Sophia Ippolito[1,2], Colin Murphy[1,2], Dana E Williams[1,2], Jolisa Velazquez[3], William Barriera[3], Silvana Guzman[3], Mark C Ladd[2*]

SUPPLEMENTARY MATERIALS AND METHODS

Modeling sedimentation scenarios

Using the results from our survivorship experiments, we devised multiple scenarios where we predicted the potential impact of multiple sedimentation events on the abundance of coral recruits on a reef. We used Port Everglades as a case study, and we delimited the area of hard-bottom habitat around the port that has been impacted by sediment plumes during previous dredging projects. We used the report of the US Army Corps of Engineers (USACE)[1] along with the spatial information obtained from maps of the port stored at the National Oceanographic and Atmospheric Administration's GeoPlatform to delimit the area of hard-bottom habitats that would be impacted by dredging activity. The USACE report analyzes the impact of four different scenarios based on different dredging strategies, some of which mitigate the formation of sediment plumes but are more financially and logistically costly, and others that are less costly but aggravate the spatial extent of sediment plumes. For this hypothetical scenario, we chose the scenario that would produce a sediment plume covering the largest extent of the adjacent reef. To estimate the recruit population size within this area, we used the estimates of recruit density reported for southeast Florida by Harper et al.[2].

We used *Pseudodiploria strigosa* for this modeling exercise because it was the only species for which we assessed survival trends across two life stages (1 and 3 months old) where the control trials did not exhibit high mortality rates due to other confounding factors. First, using the mortality rates from the control trials, we estimated background mortality rates (blue line on **Fig. 5**) across 10 days when the recruits were 1 month old (days 30–40). Then, we assessed the effects of different sedimentation events during days 40–50 to assess the decrease in population size attributable to sedimentation. The sedimentation events we simulated consisted of 1) the burial of different proportions of the pool of recruits (25, 50, 75, and 100% of recruits) under 4 mm of sediment for a total of 10 days (**Figs. 5a** and **b**) the burial of all coral recruits under 4 mm of sediment for 0, 3, 5, and 10 days (**Figs. 5c** and **d**). To estimate the background mortality of two-month-old recruits, we used the percent survival data from Ritson-Williams et al.[3] for two-month-old *P. strigosa* recruits to calculate deaths per day using an exponential decay model. Using this same methodology, we assessed the decrease in population size for the remaining coral population at the age of 3 months. To simulate the sedimentation events, we used the hazard ratios calculated by our Cox proportional hazards models for burial under 4 mm of sediment (**Figs. 2c** and **g**) to determine the magnitude of the decrease in coral populations for each age group.

SUPPLEMENTARY TABLES

Table S1: Outputs for the generalized linear mixed effects models with a logit link function testing the relationship between sediment load on top of settlement tiles and the presence of coral settlers on top of the tiles, and its variation among species. Cnat: *Colpohyllia natans*, Dlab: *Diploria labyrinthiformis*, Ofav: *Orbicella faveolata*, Pcli: *Pseudodiploria clivosa*, Pstr: *Pseudodiploria strigosa*.

	Estimate	Std. Error	z	p
**Intercept**	-30.184	4.601	-6.561	5.36×10^{-11}
**Dlab**	27.018	4.603	5.869	4.38×10^{-9}
**Ofav**	25.040	4.659	5.375	7.67×10^{-8}
**Pcli**	26.937	4.611	5.842	5.14×10^{-9}
**Pstr**	26.520	4.617	5.744	9.27×10^{-9}
**Sediment load**	- 31.561	5.015	-6.294	3.10×10^{-10}
**Dlab*Sediment load**	30.565	5.019	6.090	1.13×10^{-9}
**Ofav*Sediment load**	28.938	5.084	5.691	1.26×10^{-9}
**Pcli*Sediment load**	28.775	5.027	5.724	1.04×10^{-8}
**Pstr*Sediment load**	29.029	5.035	5.765	8.17×10^{-9}

Table S2: Outputs for the generalized linear mixed effects models with a logit link function testing the relationship between sediment load present around settlement tiles and the presence of coral settlers on top of the tiles, and its variation among species.

	Estimate	Std. Error	z	p
**Intercept**	-1.989	0.181	-10.975	$2x10^{-16}$
**Dlab**	-0.229	0.186	-1.236	0.217
**Ofav**	-1.703	0.263	-6.479	$9.23x10^{-11}$
**Pcli**	0.869	0.168	5.188	$2.12x10^{-7}$
**Pstr**	0.328	0.184	1.782	0.0747
**Sediment load**	-1.090	0.142	-7.698	$1.38x10^{-14}$
**Dlab*Sediment load**	0.967	0.181	5.350	$8.79x10^{-8}$
**Ofav*Sediment load**	0.145	0.251	0.578	0.5633
**Pcli*Sediment load**	0.702	0.163	4.315	$1.59x10^{-5}$
**Pstr*Sediment load**	0.346	0.181	1.918	0.0552

Table S3: Mean (±SE) values for the water quality parameters assessed for each treatment of the water quality assays across treatments for three days.

	**pH**	**Salinity (ppt)**	**Ammonia (mg L^{-1})**	**Nitrite (mg L^{-1})**	**Nitrate (mg L^{-1})**	**Sulfide (µg L^{-1})**
**Water source (day 0)**	8.14	35.00	0.028 ±0.007	0.0016 ±0.0002	1.50 ±0.09	10.4 ±0.5
**24 hours after start of experiment**						
	**pH**	**Salinity (ppt)**	**Ammonia (mg L^{-1})**	**Nitrite (mg L^{-1})**	**Nitrate (mg L^{-1})**	**Sulfide (µg L^{-1})**
**Control**	8.454 ±0.009	36.00 ±0.08	0.000 ±0.000	0.0012 ±0.0002	1.28 ±0.13	0.2 ±0.2
**KL sprinkle**	8.472 ±0.030	36.26 ±0.02	0.000 ±0.000	0.0050 ±0.0000	1.56 ±0.16	0.0 ±0.0
**KL 2 mm**	8.400 ±0.013	36.36 ±0.02	0.040 ±0.009	0.0062 ±0.0002	1.46 ±0.04	0.0 ±0.0
**PEV sprinkle**	8.426 ±0.026	36.52 ±0.11	0.000 ±0.000	0.0010 ±0.0000	1.28 ±0.06	0.0 ±0.0

**PEV 2 mm**	8.376 ±0.018	36.40 ±0.00	0.064 ±0.013	0.0058 ±0.0004	1.38 ±0.05	0.0 ±0.0
**PEV 4 mm**	8.288 ±0.007	35.96 ±0.02	0.118 ±0.037	0.0082 ±0.0004	1.74 ±0.07	0.0 ±0.0
**48 hours after start of experiment**						
	**pH**	**Salinity (ppt)**	**Ammonia (mg L^{-1})**	**Nitrite (mg L^{-1})**	**Nitrate (mg L^{-1})**	**Sulfide (µg L^{-1})**
**Control**	8.578 ±0.021	36.06 ±0.12	0.010 ±0.010	0.0032 ±0.0004	1.14 ±0.10	0.0 ±0.0
**KL sprinkle**	8.578 ±0.007	36.00 ±0.08	0.002 ±0.002	0.0056 ±0.0005	1.18 ±0.04	0.0 ±0.0
**KL 2 mm**	8.514 ±0.032	36.22 ±0.02	0.026 ±0.012	0.0070 ±0.0009	1.22 ±0.04	0.0 ±0.0
**PEV sprinkle**	8.568 ±0.047	36.02 ±0.04	0.000 ±0.000	0.0026 ±0.0004	1.18 ±0.07	0.0 ±0.0
**PEV 2 mm**	8.500 ±0.035	36.26 ±0.02	0.016 ±0.016	0.0052 ±0.0015	1.36 ±0.07	0.2 ±0.2
**PEV 4 mm**	8.314 ±0.015	36.48 ±0.04	0.092 ±0.034	0.0082 ±0.0002	1.54 ±0.08	2.6 ±0.4
**72 hours after start of experiment**						
	**pH**	**Salinity (ppt)**	**Ammonia (mg L^{-1})**	**Nitrite (mg L^{-1})**	**Nitrate (mg L^{-1})**	**Sulfide (µg L^{-1})**
**Control**	8.674 ±0.029	36.06 ±0.06	0.004 ±0.004	0.0058 ±0.0002	1.22 ±0.07	5.0 ±0.0
**KL sprinkle**	8.756 ±0.055	36.26 ±0.02	0.000 ±0.000	0.0076 ±0.0007	1.44 ±0.04	7.4 ±0.2
**KL 2 mm**	8.602 ±0.031	36.24 ±0.07	0.024 ±0.015	0.0076 ±0.0004	1.46 ±0.02	0.0 ±0.0
**PEV sprinkle**	8.752 ±0.034	36.18 ±0.04	0.000 ±0.000	0.0054 ±0.0002	1.30 ±0.09	5.4 ±0.2
**PEV 2 mm**	8.534 ±0.038	36.34 ±0.04	0.010 ±0.008	0.0084 ±0.0009	1.76 ±0.11	7.4 ±0.8
**PEV 4 mm**	8.430 ±0.025	36.54 ±0.04	0.192 ±0.050	0.0132 ±0.0030	1.56 ±0.08	3.4 ±0.7

Table S4: Contrast tables of pairwise comparisons from the similarity of percentages (SIMPER) test on the water quality parameters assessed for each treatment of the water quality assays. The rows highlighted in gray indicate variables that were significantly different ($p < 0.05$) for the respective treatment pairs.

Control–PE sprinkle							
	**Average**	**SD**	**Ratio**	**Ava**	**Avb**	**Cum. sum**	**p**
**Ammonia**	-0.022	0.047	-0.467	-0.425	-0.479	0.027	0.774
**Sulfide**	-0.023	0.030	-0.762	0.314	0.396	0.056	0.427
**Nitrite**	-0.047	0.049	-0.961	-0.590	-0.698	0.114	0.780
**pH**	-0.198	0.175	-1.130	0.356	0.906	0.357	0.754
**Salinity**	-0.235	0.175	-1.344	-1.179	-0.503	0.645	0.746
**Nitrate**	-0.289	0.247	-1.170	-1.051	-0.683	1.000	0.769
**Control–KL sprinkle**							
	**Average**	**SD**	**Ratio**	**Ava**	**Avb**	**Cum. sum**	**p**
**Ammonia**	0.065	0.439	0.148	-0.425	-0.479	-0.020	0.627
**Nitrite**	-0.073	1.939	-0.038	-0.590	-0.106	0.002	0.800
**Sulfide**	-0.342	1.779	-0.192	0.314	0.756	0.107	0.761
**Nitrate**	-0.366	2.403	-0.152	-1.051	-0.032	0.220	0.807
**pH**	-0.788	6.536	-0.121	0.356	0.932	0.461	0.902
**Salinity**	-1.756	6.767	-0.260	-1.179	-0.054	1.000	0.994
**Control–PE 2 mm**							
	**Average**	**SD**	**Ratio**	**Ava**	**Avb**	**Cum. sum**	**p**
**Nitrite**	-0.122	1.499	-0.081	-0.590	0.109	0.064	0.846
**Sulfide**	-0.144	0.895	-0.161	0.314	0.733	0.140	0.564
**Ammonia**	-0.219	0.506	-0.432	-0.425	-0.344	0.254	0.948
**Salinity**	-0.288	1.755	-0.164	-1.179	0.394	0.405	0.776
**pH**	-0.466	0.717	-0.650	0.356	-0.644	0.649	0.847
**Nitrate**	-0.669	4.648	-0.144	-1.051	1.247	1.000	0.911
**Control–KL 2 mm**							
	**Average**	**SD**	**Ratio**	**Ava**	**Avb**	**Cum. sum**	**p**
**Ammonia**	-0.084	0.098	-0.860	-0.425	-0.159	0.065	0.851
**Nitrite**	-0.112	0.091	-1.233	-0.590	-0.106	0.150	0.827
**pH**	-0.125	0.098	-1.277	0.356	-0.156	0.247	0.706
**Nitrate**	-0.216	0.132	-1.630	-1.051	0.058	0.412	0.712
**Salinity**	-0.261	0.214	-1.221	-1.179	-0.167	0.613	0.757
**Sulfide**	-0.504	0.168	-2.996	0.314	-2.049	1.000	0.865

Control–PE 4 mm							
	**Average**	**SD**	**Ratio**	**Ava**	**Avb**	**Cum. sum**	**p**
**Ammonia**	2.271	7.351	0.309	-0.425	1.886	0.327	0.060
**Salinity**	1.784	7.248	0.246	-1.179	1.510	0.584	0.063
**pH**	1.066	4.599	0.232	0.356	-1.394	0.737	0.099
**Nitrite**	0.991	2.326	0.426	-0.590	1.392	0.880	0.112
**Sulfide**	0.502	1.728	0.291	0.314	-0.149	0.952	0.084
**Nitrate**	0.333	2.648	0.126	-1.051	0.461	1.000	0.305
**PE sprinkle–KL sprinkle**							
	**Average**	**SD**	**Ratio**	**Ava**	**Avb**	**Cum. sum**	**p**
**Nitrate**	3.467	11.807	0.294	-0.683	-0.032	0.374	0.011
**Nitrite**	2.042	7.777	0.263	-0.698	-0.106	0.595	0.068
**Salinity**	1.646	5.608	0.294	-0.503	-0.054	0.773	0.074
**pH**	1.494	5.282	0.283	0.906	0.932	0.934	0.071
**Sulfide**	0.608	2.325	0.262	0.396	0.756	1.000	0.075
**Ammonia**	0.000	0.000	NaN	-0.479	-0.479	1.000	NA
**PE sprinkle–PE 2 mm**							
	**Average**	**SD**	**Ratio**	**Ava**	**Avb**	**Cum. sum**	**p**
**Ammonia**	-0.240	1.495	-0.161	-0.479	-0.344	0.022	0.952
**Sulfide**	-0.716	2.671	-0.268	0.396	0.733	0.086	0.944
**Nitrite**	-1.477	5.719	-0.258	-0.698	0.109	0.219	1.000
**Salinity**	-2.046	4.663	-0.439	-0.503	0.394	0.403	0.998
**Nitrate**	-2.463	6.413	-0.384	-0.683	1.247	0.624	1.000
**pH**	-4.179	12.100	-0.345	0.906	-0.644	1.000	1.000
**PE sprinkle–KL 2 mm**							
	**Average**	**SD**	**Ratio**	**Ava**	**Avb**	**Cum. sum**	**p**
**Ammonia**	-0.146	0.218	-0.668	-0.479	-0.159	0.070	0.910
**Nitrite**	-0.220	0.236	-0.935	-0.698	-0.106	0.177	0.907
**Salinity**	-0.278	0.296	-0.940	-0.503	-0.167	0.311	0.776
**Nitrate**	-0.301	0.206	-1.460	-0.683	0.058	0.456	0.772
**pH**	-0.324	0.223	-1.457	0.906	-0.156	0.613	0.809
**Sulfide**	-0.803	0.455	-1.764	0.396	-2.049	1.000	0.965
**PE sprinkle–PE 4 mm**							
	**Average**	**SD**	**Ratio**	**Ava**	**Avb**	**Cum. sum**	**p**
**pH**	2.272	3.571	0.636	0.906	-1.394	0.244	0.054
**Salinity**	1.941	2.824	0.687	-0.503	1.510	0.453	0.061
**Ammonia**	1.778	2.087	0.852	-0.479	1.886	0.645	0.077
**Nitrate**	1.528	3.180	0.480	-0.683	0.461	0.809	0.111
**Nitrite**	1.177	1.386	0.849	-0.698	1.392	0.936	0.112

**Sulfide**	0.597	0.837	0.713	0.396	-0.149	1.000	0.072
**KL sprinkle–PE 2 mm**							
	**Average**	**SD**	**Ratio**	**Ava**	**Avb**	**Cum. sum**	**p**
**pH**	1.086	2.160	0.503	0.932	-0.644	0.373	0.100
**Nitrate**	0.884	1.990	0.444	-0.032	1.247	0.676	0.153
**Salinity**	0.477	1.252	0.381	-0.054	0.394	0.840	0.237
**Nitrite**	0.257	0.326	0.788	-0.106	0.109	0.928	0.362
**Sulfide**	0.116	0.317	0.366	0.756	0.733	0.968	0.264
**Ammonia**	0.0944	0.169	0.5587	-0.479	-0.344	1	0.5776
**KL sprinkle–KL 2 mm**							
	**Average**	**SD**	**Ratio**	**Ava**	**Avb**	**Cum. sum**	**p**
**Sulfide**	16.776	88.030	0.191	0.756	-2.049	0.488	0.057
**Ammonia**	5.062	25.630	0.198	-0.479	-0.159	0.635	0.044
**Salinity**	3.419	17.950	0.190	-0.054	-0.167	0.734	0.043
**Nitrite**	3.285	17.260	0.190	-0.106	-0.106	0.830	0.052
**pH**	3.177	16.010	0.198	0.932	-0.156	0.922	0.037
**Nitrate**	2.690	13.860	0.194	-0.032	0.058	1.000	0.043
**KL sprinkle–PE 4 mm**							
	**Average**	**SD**	**Ratio**	**Ava**	**Avb**	**Cum. sum**	**p**
**pH**	0.600	0.284	2.113	0.932	-1.394	0.249	0.222
**Ammonia**	0.597	0.393	1.520	-0.479	1.886	0.496	0.181
**Salinity**	0.454	0.320	1.418	-0.054	1.510	0.685	0.251
**Nitrite**	0.273	0.168	1.632	-0.106	1.392	0.798	0.342
**Sulfide**	0.265	0.175	1.513	0.756	-0.149	0.908	0.152
**Nitrate**	0.222	0.225	0.990	-0.032	0.461	1.000	0.365
**PE 2 mm–KL 2 mm**							
	**Average**	**SD**	**Ratio**	**Ava**	**Avb**	**Cum. sum**	**p**
**Salinity**	-0.017	0.858	-0.019	0.394	-0.167	0.007	0.571
**Nitrite**	-0.069	0.840	-0.083	0.109	-0.106	0.035	0.793
**Nitrate**	-0.185	1.677	-0.110	1.247	0.058	0.112	0.690
**Ammonia**	-0.375	0.973	-0.386	-0.344	-0.159	0.267	0.989
**pH**	-0.432	0.995	-0.434	-0.644	-0.156	0.446	0.833
**Sulfide**	-1.341	3.619	-0.370	0.733	-2.049	1.000	0.998
**PE 2 mm–PE 4 mm**							
	**Average**	**SD**	**Ratio**	**Ava**	**Avb**	**Cum. sum**	**p**
**Ammonia**	0.520	0.365	1.426	-0.344	1.886	0.308	0.206
**Salinity**	0.305	0.233	1.309	0.394	1.510	0.488	0.318
**Nitrite**	0.250	0.164	1.525	0.109	1.392	0.635	0.365
**Sulfide**	0.228	0.140	1.622	0.733	-0.149	0.770	0.173

**pH**	0.204	0.165	1.240	-0.644	-1.394	0.890	0.460
**Nitrate**	0.186	0.159	1.167	1.247	0.461	1.000	0.387
**KL 2 mm–PE 4 mm**							
	**Average**	**SD**	**Ratio**	**Ava**	**Avb**	**Cum. sum**	**p**
**Nitrite**	0.092	1.112	0.083	-0.106	1.392	-0.104	0.588
pH	-0.019	1.901	-0.010	-0.156	-1.394	-0.083	0.649
Ammonia	-0.041	2.827	-0.014	-0.159	1.886	-0.036	0.799
Nitrate	-0.102	1.168	-0.087	0.058	0.461	0.079	0.631
Sulfide	-0.250	2.282	-0.109	-2.049	-0.149	0.363	0.681
Salinity	-0.561	2.432	-0.231	-0.167	1.510	1.000	0.879

Table S5: Location, depth, and approximate distance from shore of the study sites.

Site	Latitude (decimal degrees)	Longitude (decimal degrees)	Depth (m)	Distance from shore (m)
Fort Lauderdale	26.212200	-80.086683	6	439
Dania Beach	26.011577	-80.112620	6	277
South Canyon	25.85015	-80.10014	6	1200
Emerald Reef	25.66681	-80.08357	8	7000
Carysfort Reef	24.221947	-80.21145	8	9000
Conch Reef	25.22273	-80.20875	8	9000

Table S6: Average (±SE) sedimentation rate (g m^{-2} day^{-1}), LSAT benthic cover (%), and median (±MAD) LSAT sediment depth for each site surveyed during the summer of 2023.

Site	Sedimentation rate (g m^{-2} day^{-1})	LSAT benthic cover (%)	LSAT sediment depth (mm)
Ft Lauderdale	3.44 ±0.68	36.8 ±2.5	1 ±0.0
Dania Beach	2.90 ±0.35	46.3 ±4.6	2 ±1.5
South Canyon	2.96 ±0.46	34.2 ±3.6	1 ±1.5
Emerald Reef	5.05 ±1.23	31.0 ±3.4	2 ±0.0
Carysfort Reef	7.33 ±0.71	46.2 ±3.4	2 ±1.5
Conch Reef	9.74 ±1.75	58.5 ±4.1	3 ±1.5

Table S7: Dunn's multiple comparison test of median LSAT sediment depth across sites. Within each cell, the upper value is Dunn's pairwise z-test statistic, and the lower value is the raw p-value associated with each comparison. Significant pairwise comparisons are highlighted in gray.

	Carysfort Reef	Conch Reef	Dania Beach	Emerald Reef	Ft Lauderdale
**Conch Reef**	-4.07<br>0.00				
**Dania Beach**	-0.38<br>0.35	3.69<br>0.00			
**Emerald Reef**	-1.56<br>0.06	2.51<br>0.01	-1.18<br>0.12		
**Ft Lauderdale**	1.28<br>0.10	5.35<br>0.00	1.66<br>0.05	2.84<br>0.00	
**South Canyon**	1.48<br>0.07	5.55<br>0.00	1.86<br>0.03	3.04<br>0.00	0.20<br>0.42

Table S8: Tukey HSD post-hoc, pairwise comparisons of mean sedimentation rates across sites. Significant pairwise comparisons are highlighted in gray.

Pair	Difference	Lower 95% CI	Upper 95% CI	p
Dania Beach-Ft Lauderdale	-0.1704	-1.0040	0.6631	0.990
South Canyon-Ft Lauderdale	-0.1887	-1.0223	0.6448	0.984
Emerald Reef-Ft Lauderdale	0.2265	-0.6071	1.0600	0.964
Carysfort Reef-Ft Lauderdale	0.7964	-0.0371	1.6300	0.069
Conch Reef-Ft Lauderdale	1.0565	0.0356	2.0774	0.039
South Canyon-Dania Beach	-0.0183	-0.6989	0.6623	1.000
Emerald Reef-Dania Beach	0.3969	-0.2837	1.0775	0.514
Carysfort Reef-Dania Beach	0.9669	0.2863	1.6475	0.002
Conch Reef-Dania Beach	1.2270	0.3266	2.1273	0.003
Emerald Reef-South Canyon	0.4152	-0.2654	1.0958	0.465
Carysfort Reef-South Canyon	0.9852	0.3046	1.6658	0.001
Conch Reef-South Canyon	1.2453	0.3449	2.1456	0.002
Carysfort Reef-Emerald Reef	0.5700	-0.1106	1.2505	0.148
Conch Reef-Emerald Reef	0.8300	-0.0703	1.7304	0.086
Conch Reef-Carysfort Reef	0.2601	-0.6403	1.1604	0.954

Table S9: Tukey HSD post-hoc, pairwise comparisons of mean LSAT cover across sites. Significant pairwise comparisons are highlighted in gray.

Pair	Difference	Lower 95% CI	Upper 95% CI	p
Dania Beach-Ft Lauderdale	9.48	-5.38	24.34	0.44
South Canyon-Ft Lauderdale	-2.60	-17.46	12.26	1.00
Emerald Reef-Ft Lauderdale	-5.76	-20.62	9.10	0.87
Carysfort Reef-Ft Lauderdale	9.40	-5.46	24.26	0.45
Conch Reef-Ft Lauderdale	21.68	6.82	36.54	<0.001
South Canyon-Dania Beach	-12.08	-26.94	2.78	0.18
Emerald Reef-Dania Beach	-15.24	-30.10	-0.38	0.04
Carysfort Reef-Dania Beach	-0.08	-14.94	14.78	1.00
Conch Reef-Dania Beach	12.20	-2.66	27.06	0.17
Emerald Reef-South Canyon	-3.16	-18.02	11.70	0.99
Carysfort Reef-South Canyon	12.00	-2.86	26.86	0.19
Conch Reef-South Canyon	24.28	9.42	39.14	<0.0001
Carysfort Reef-Emerald Reef	15.16	0.30	30.02	0.04
Conch Reef-Emerald Reef	27.44	12.58	42.30	0.00000
Conch Reef-Carysfort Reef	12.28	-2.58	27.14	0.17

Table S10. Average coral recruit densities within 25 plots (0.25 x 0.25 m) by site during the summer of 2023.

**Site**	***Siderastrea* spp. density (ind m^{-2})**	***Porites* spp. density (ind m^{-2})**	***Montastraea* spp. density (ind m^{-2})**	***Diploria* spp. density (ind m^{-2})**	***Agaricia* spp. density (ind m^{-2})**	***Favia* spp. density (ind m^{-2})**	**Total Recruit density (ind m^{-2})**
Fort Lauderdale	1.9 ±1.1	0.6 ±0.6	0.0 ±0.0	0.0 ±0.0	0.0 ±	0.0 ±	2.6 ±1.2
Dania Beach	1.9 ±1.1	1.3 ±0.9	1.3 ±0.9	0.6 ±0.6	0.0 ±	0.0 ±	5.1 ±1.5
South Canyon	5.1 ±2.4	1.3 ±0.9	0.6 ±0.6	0.0 ±0.0	0.0 ±	0.0 ±	7.0 ±2.5
Emerald Reef	5.8 ±2.8	1.3 ±0.9	0.0 ±0.0	0.6 ±0.6	0.0 ±	0.6 ±	8.3 ±2.9
Carysfort Reef	5.8 ±3.4	1.3 ±0.9	0.0 ±0.0	0.0 ±0.0	0.6 ±	0.0 ±	7.7 ±3.7
Conch Reef	1.9 ±1.1	0.0 ±0.0	0.0 ±0.0	0.0 ±0.0	1.3 ±	0.0 ±	3.2 ±1.6
**Total**	3.7 ±0.1	1.0 ±0.0	0.3 ±0.0	0.2 ±0.0	0.3 ±0.0	0.1 ±0.0	5.7 ±0.1

Table S11: Average (±SD) relative abundance of sand grains for the ≥500, 499–250, 249–125 and <125 μm size classes.

Site	≥500 μm	250–499 μm	125–249 μm	<125 μm
Ft Lauderdale	0.11 ±0.07	0.16 ±0.03	0.53 ±0.05	0.20 ±0.06
Dania Beach	0.08 ±0.05	0.47 ±0.06	0.40 ±0.08	0.05 ±0.02
South Canyon	0.13 ±0.04	0.56 ±0.05	0.23 ±0.06	0.08 ±0.04
Emerald Reef	0.11 ±0.04	0.47 ±0.09	0.37 ±0.09	0.05 ±0.02
Carysfort Reef	0.26 ±0.08	0.34 ±0.04	0.25 ±0.04	0.15 ±0.06
Conch Reef	0.27 ±0.05	0.40 ±0.04	0.27 ±0.04	0.06 ±0.02

Table S12: Sources of larvae and recruits used for the settlement and survival assays

**Species**	**Source**
Acropora cervicornis	*Ex situ* – Florida Aquarium<br>*In situ* – Collected from nursery off of Miami, FL by the Reef Futures Lab at the University of Miami
Colpophyllia natans	*Ex situ* – Florida Aquarium
Diploria labyrinthiformis	*In situ* – Collected from upper FL Keys
Orbicella faveolata	*In situ* – Collected from Biscayne National Park, Florida and from upper FL Keys
Pseudodiploria clivosa	*Ex situ* – University of North Carolina, Wilmington and Florida Aquarium
Pseudodiploria strigosa	*Ex situ* – Florida Aquarium

SUPPLEMENTARY FIGURES

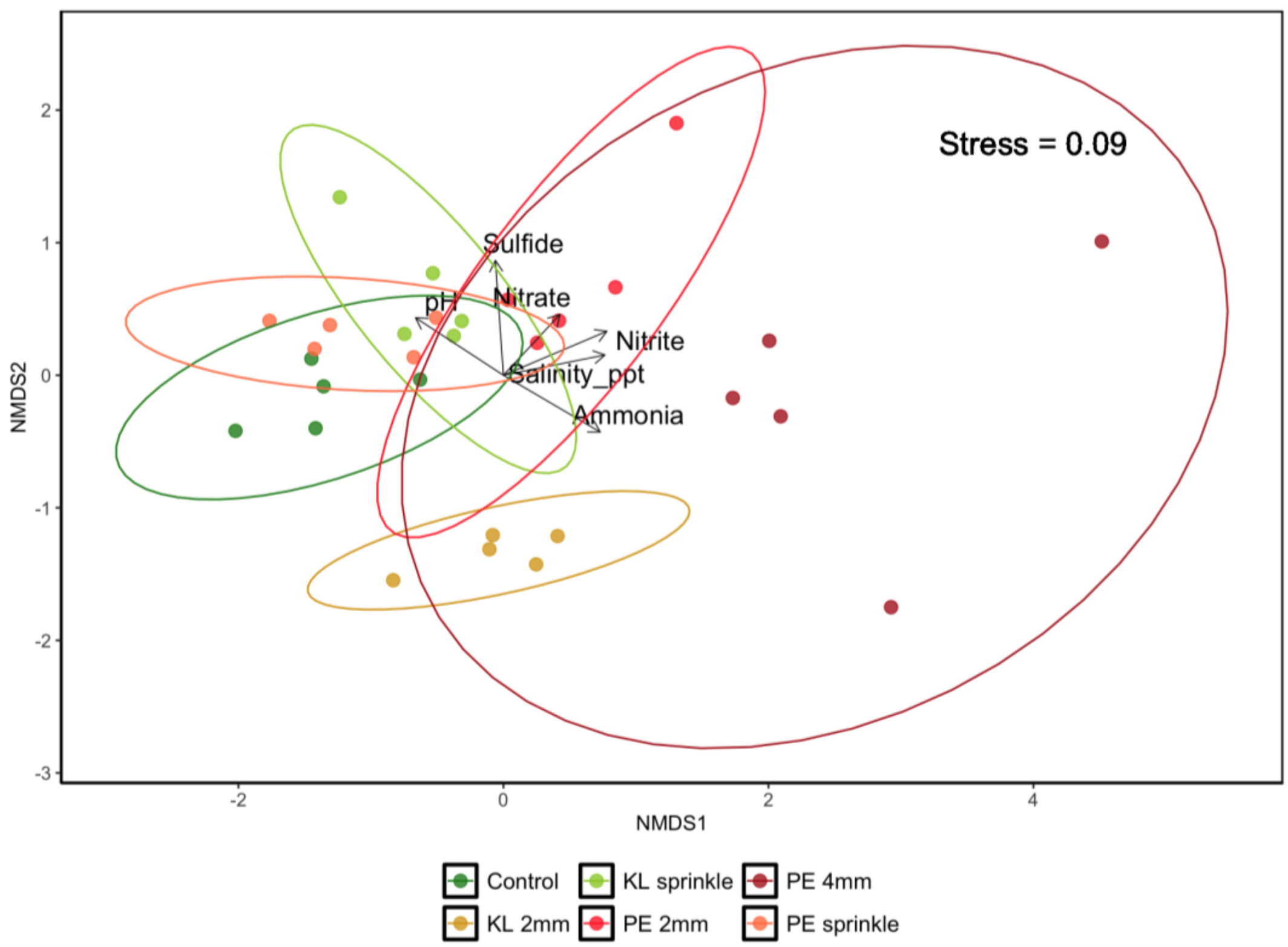

Figure S1: A non-metric multidimensional scaling (nMDS) plot showing the relationship of water quality parameters to the separation among treatments. The vectors shown are the water quality parameters that contribute the most to the separation among treatments. The ellipses represent the 95% confidence interval based on the centroid and covariance matrix of the points for each treatment.

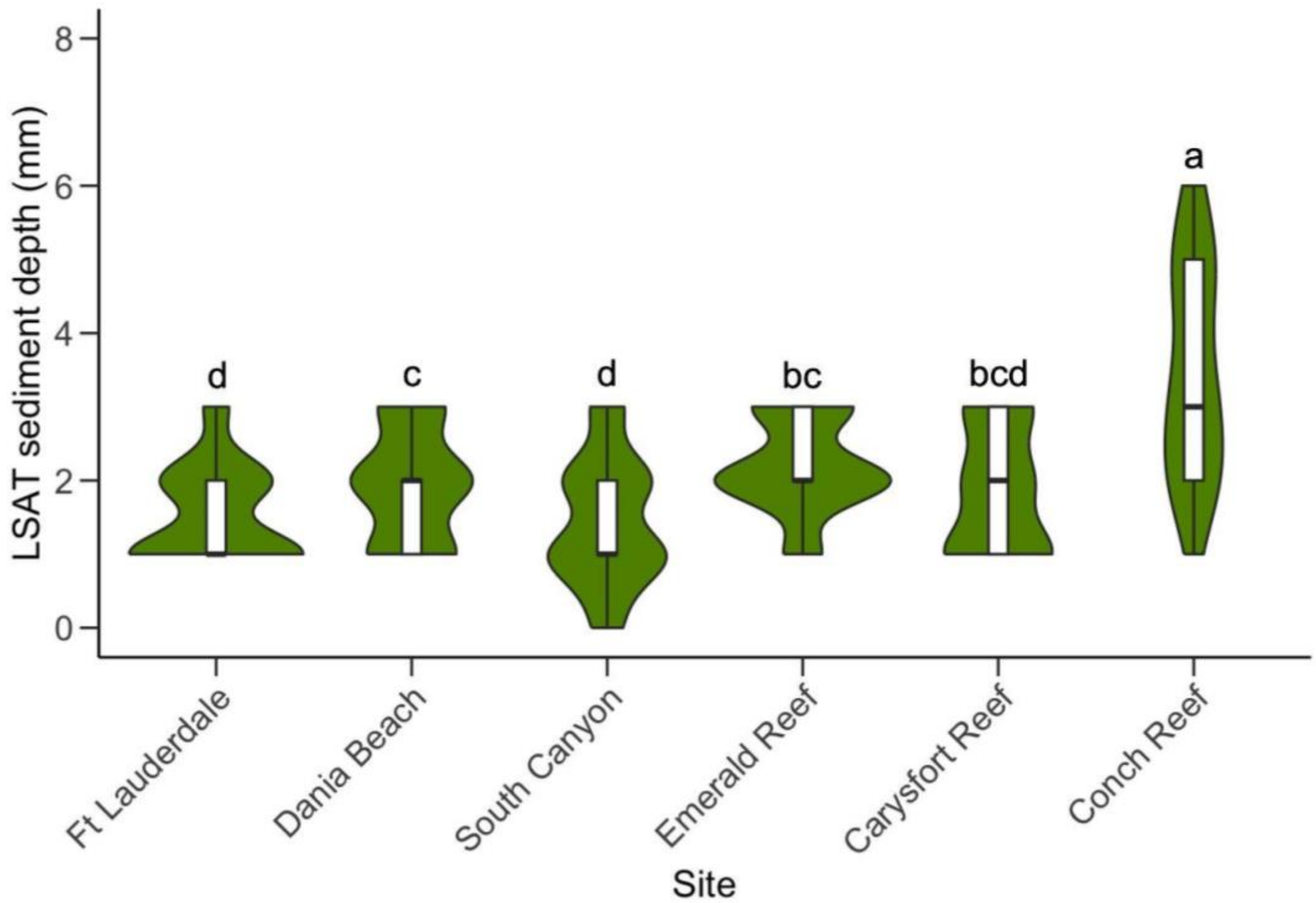

Figure S2: Box plot depicting the median (± interquartile range) and violin plot depicting LSAT sediment depth (mm) frequency distribution across our survey sites.

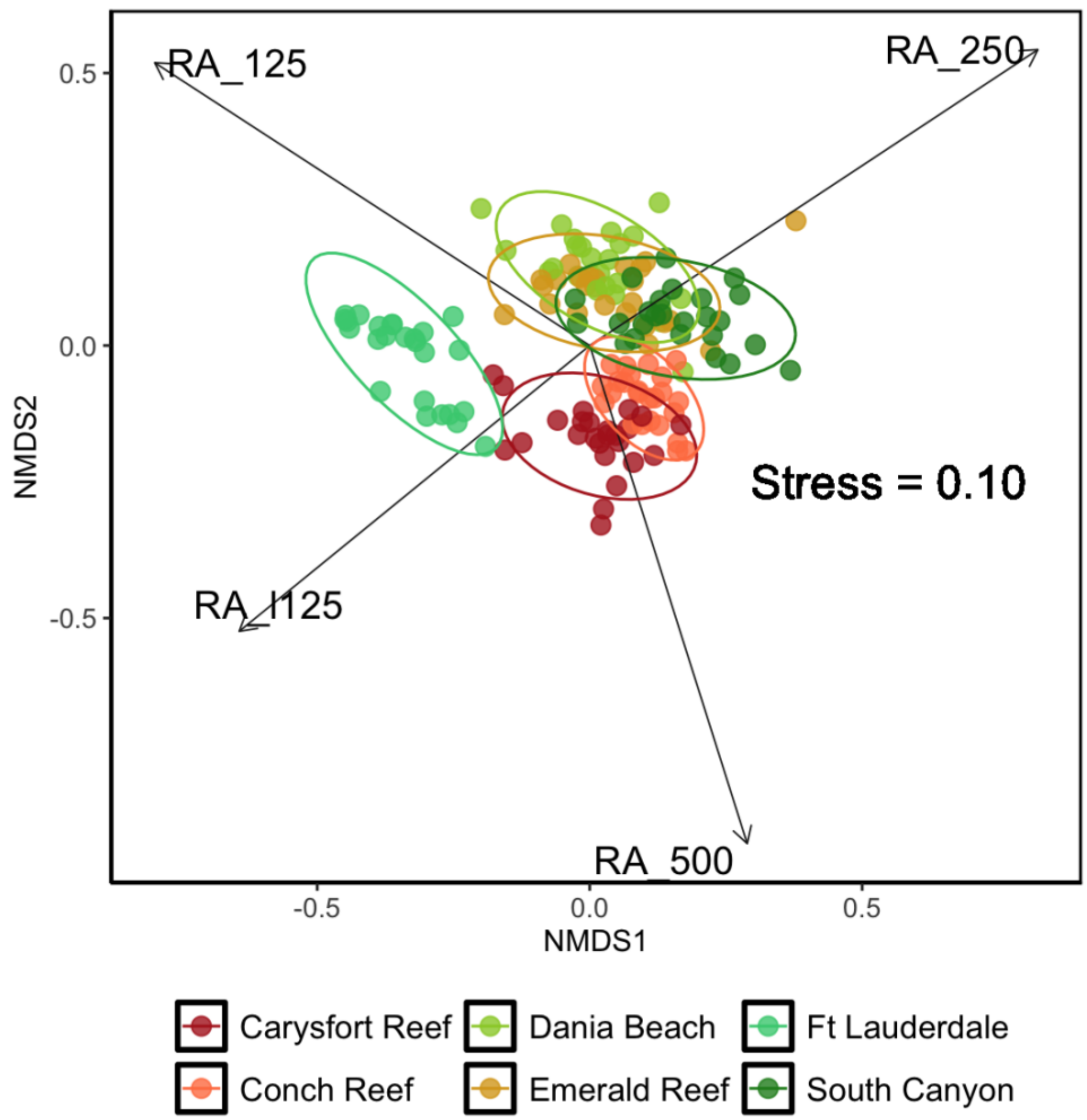

Figure S3: A non-metric multidimensional scaling (nMDS) plot showing the influence of the relative abundance of different sediment-grain size classes on the separation among sites. The species vectors shown are the sediment-grain size classes that contribute the most to the site separation. The ellipses represent the 95% confidence interval based on the centroid and covariance matrix of the points for each site. RA_500: relative abundance of sediment grains in the 500 µm size class. RA_250: relative abundance of sediment grains in the 250 µm size class. RA_125: relative abundance of sediment grains in the 125 µm size class. RA_l125: relative abundance of sediment grains in the >125 µm size class.

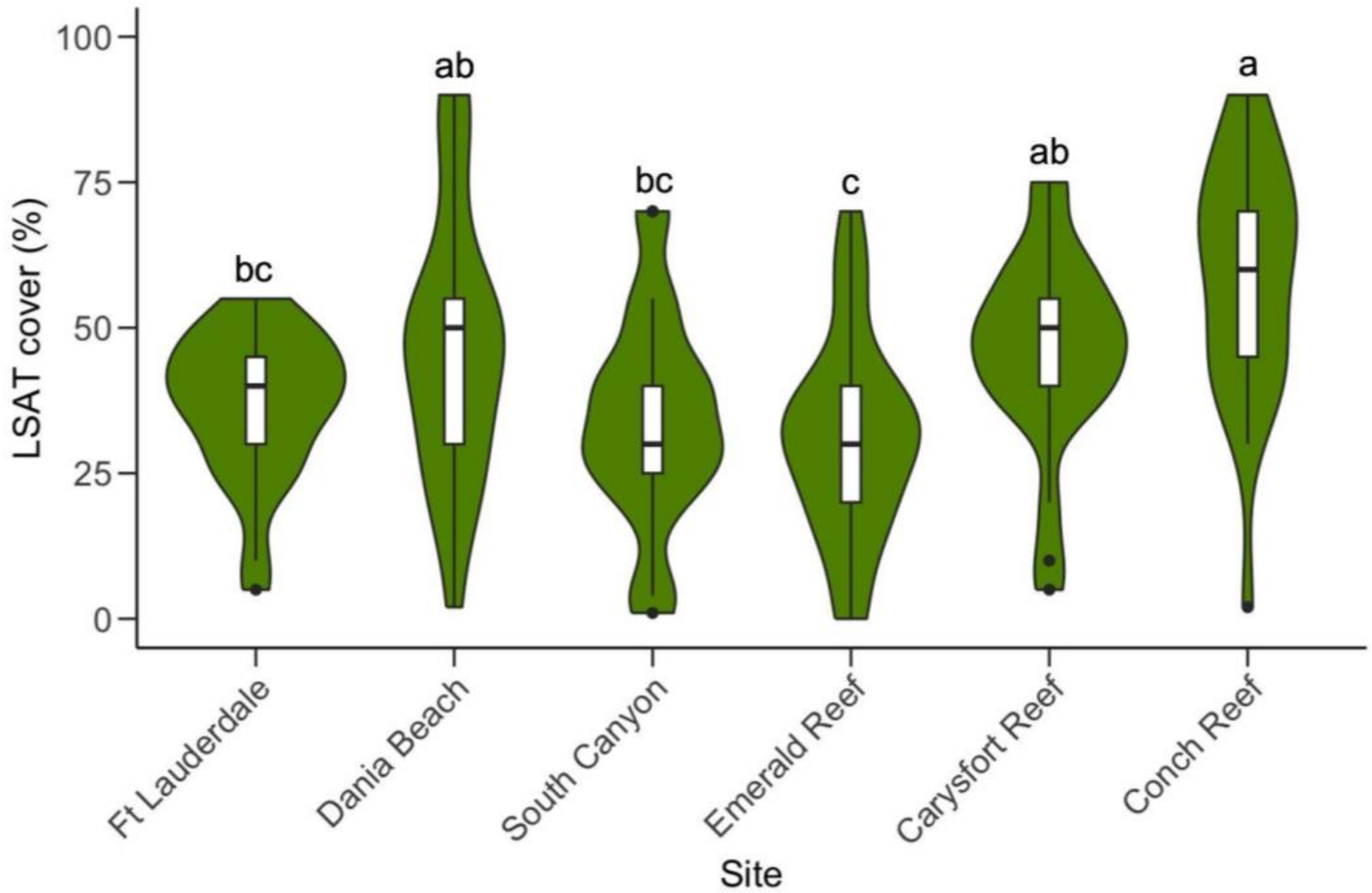

Figure S4: Box plot depicting the median (± interquartile range) and violin plot depicting the frequency distribution of LSAT percent cover (%) across our survey sites.

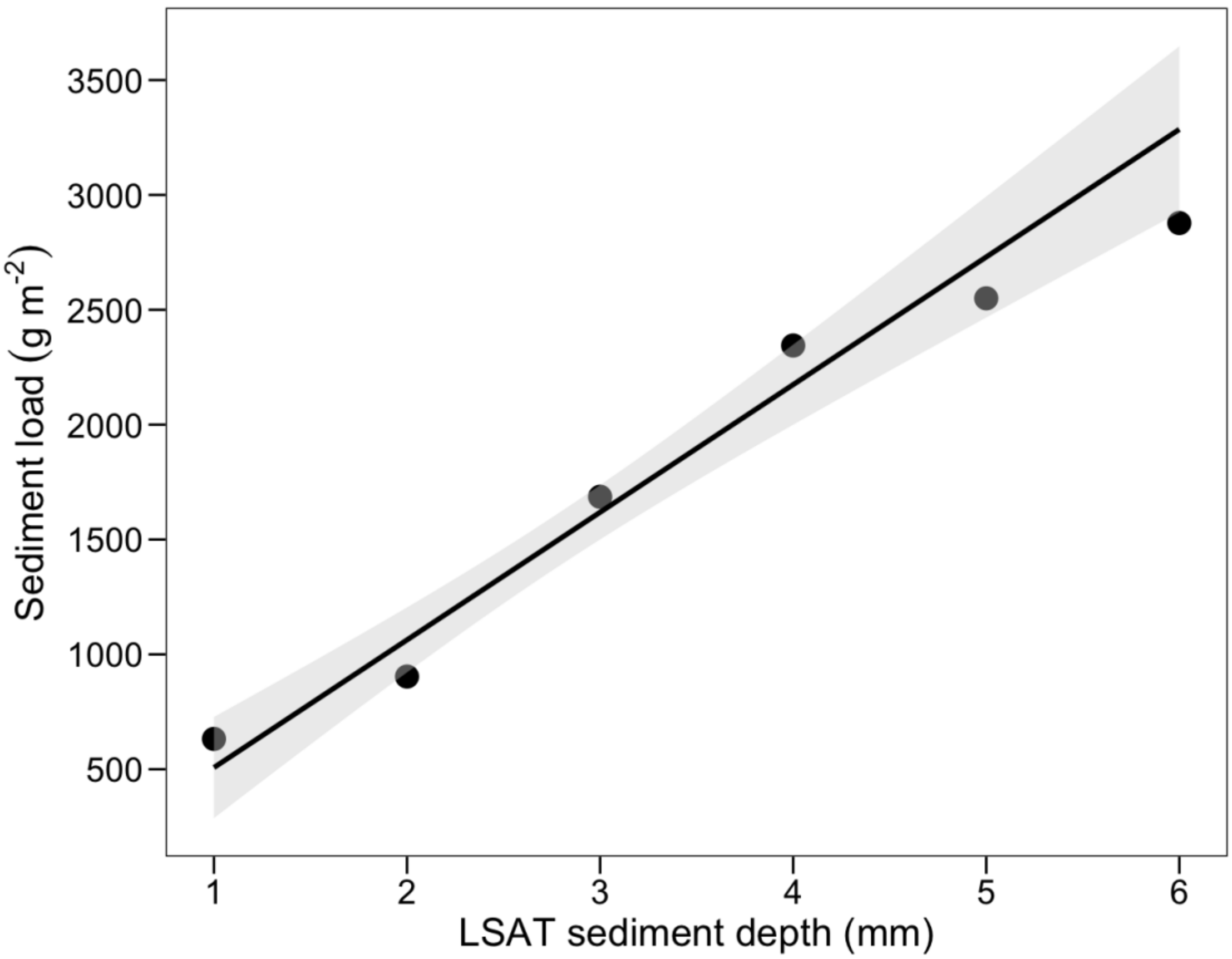

Figure S5: Sediment load (g m^{-2}) as a function of the depth (mm) of the sediment layer trapped within the long sediment-laden algal turf (LSAT) across all of our field sites. Sediment load was calculated based on the weight of sediments collected from our 5 x 5 mini plots at our field sites and the LSAT sediment depth measured within those same mini plots.

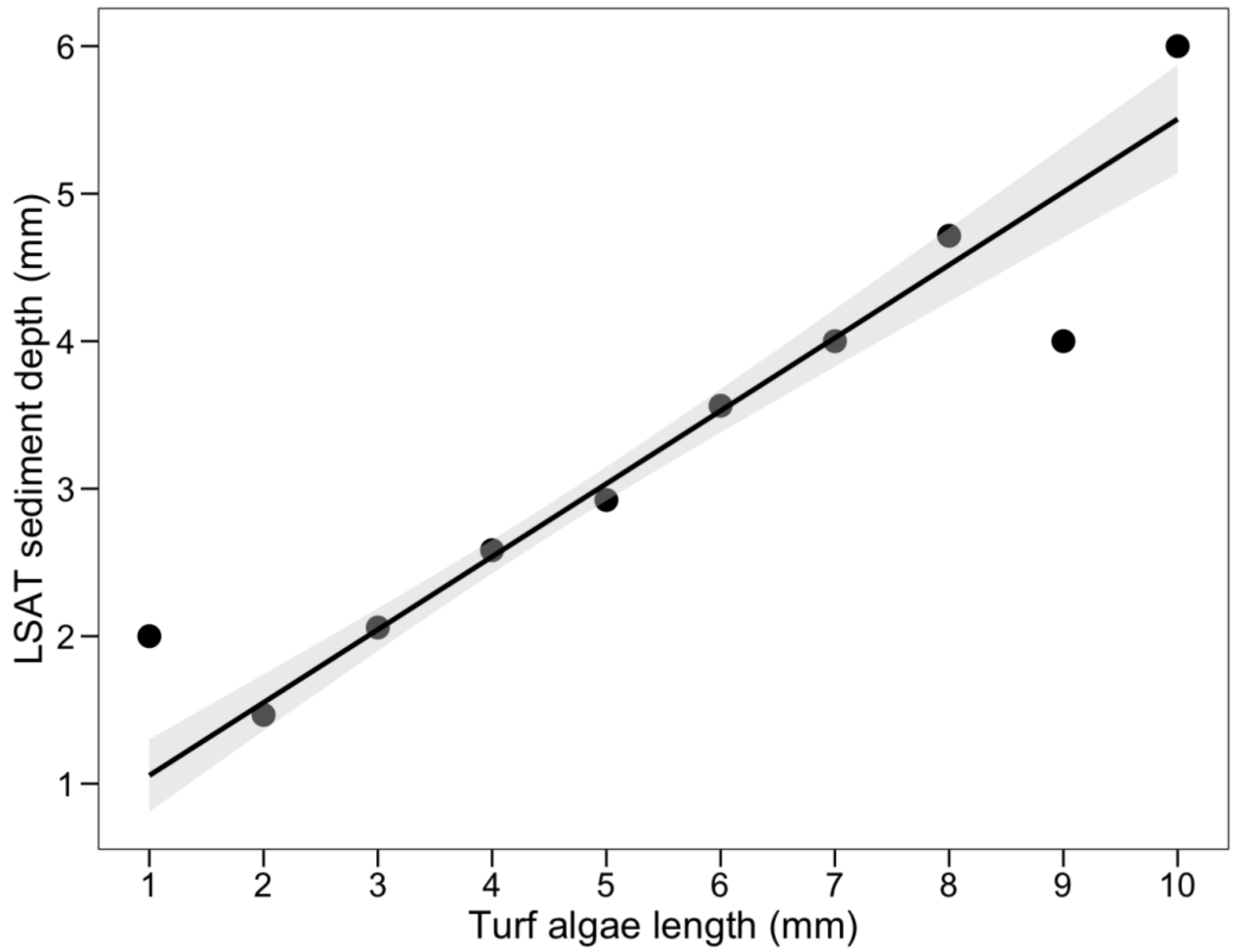

Figure S6: Relationship between LSAT sediment depth (mm) and the length of the turf algae entrapping the sediment (mm). LSAT sediment depth and turf algae length were measured within the mini plots established across our field sites.

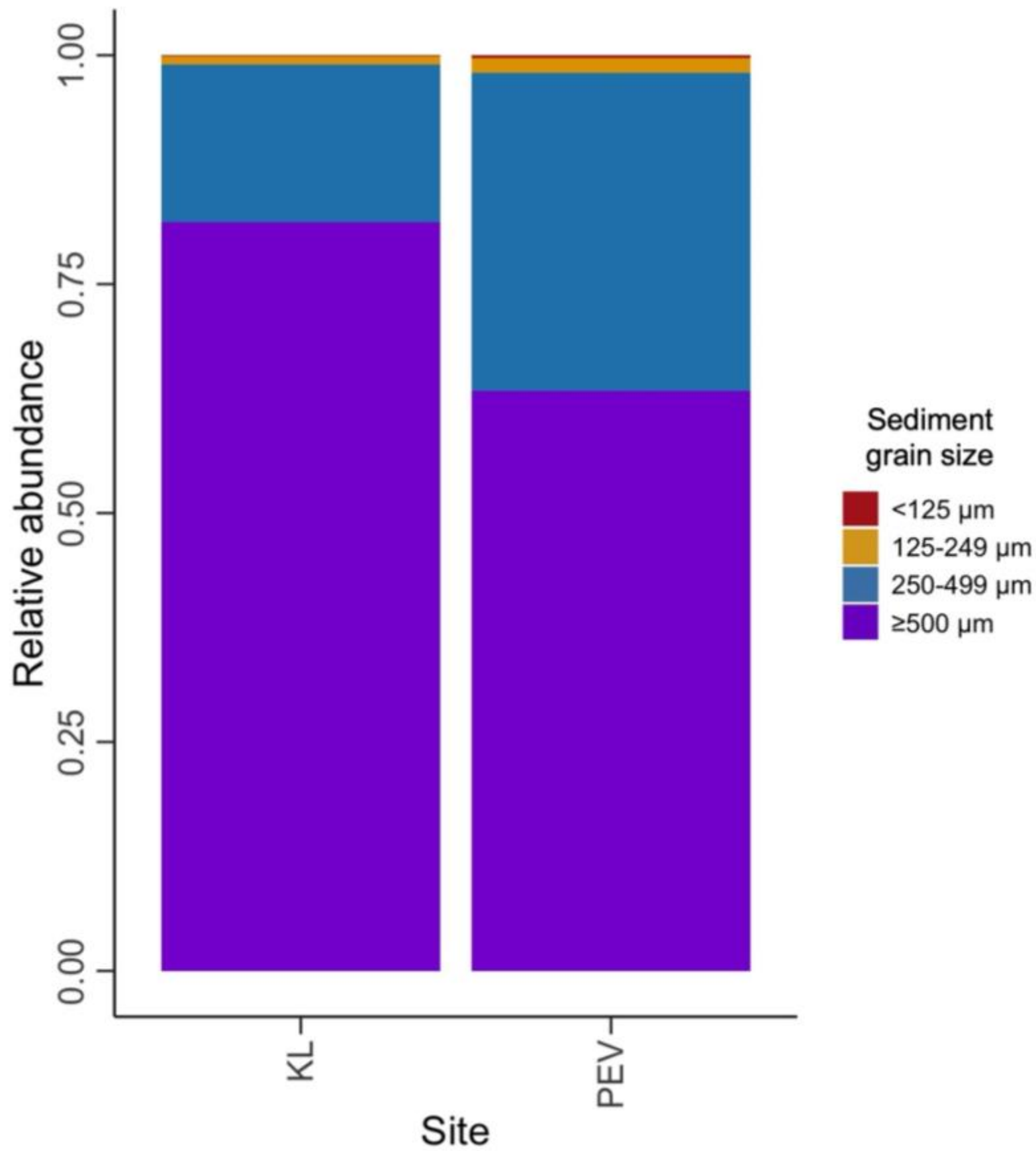

Figure S7: Relative abundance for different grain size categories measured for sediments used in our settlement assays and sediment burial survival experiments. KL = sediments collected from Key Largo, PEV = sediments collected from Port Everglades

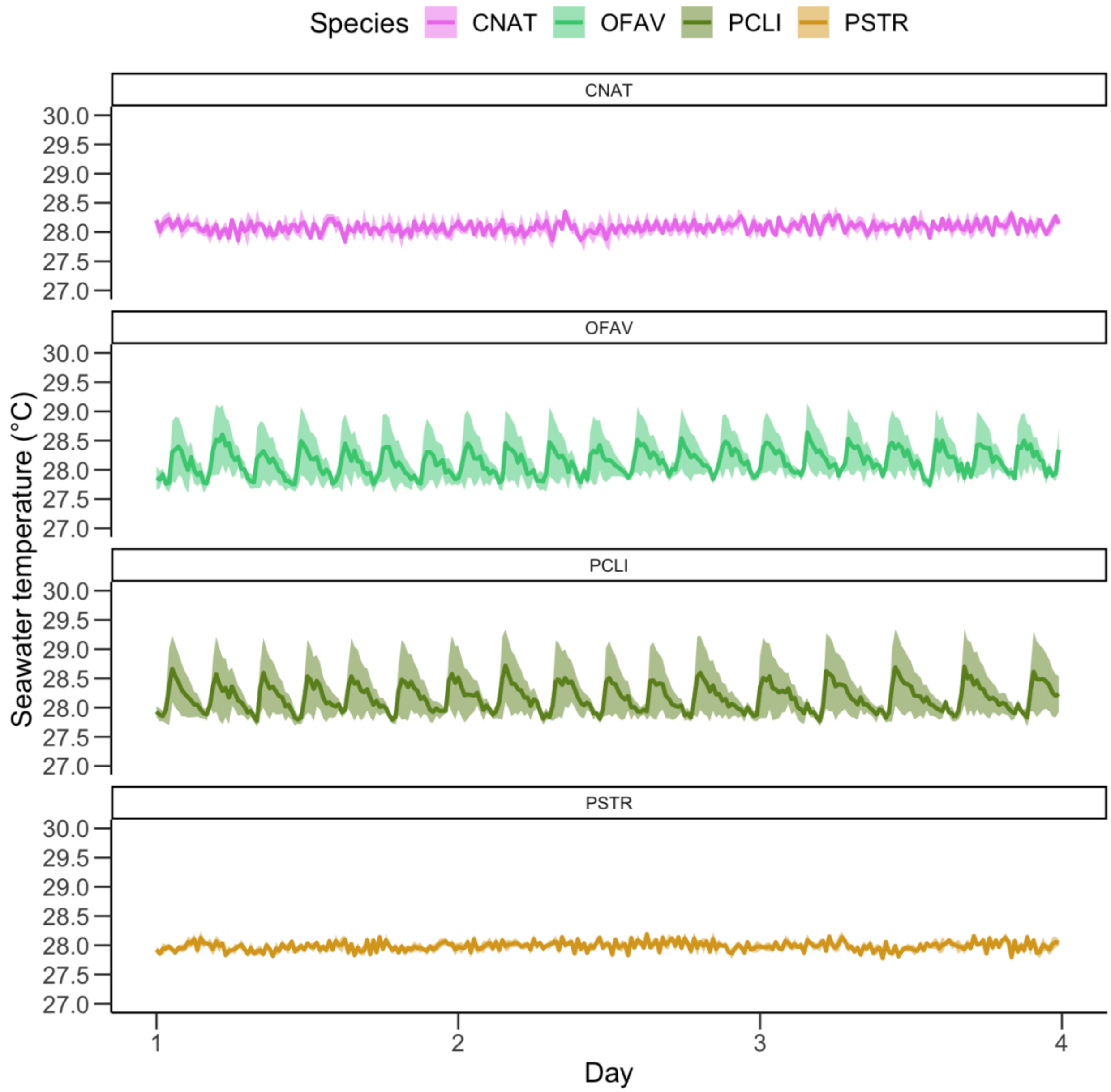

Figure S8: Mean (± SE) seawater temperature (°C) across replicates for the water baths where the larval settlement assays were conducted for each species tested. Seawater temperature was recorded every 15 minutes using a HOBO data logger. CNAT = *Colpophyllia natans*, OFAV = *Orbicella faveolata*, PCLI = *Pseudodiploria clivosa*, PSTR = *Pseudodiploria strigosa*.

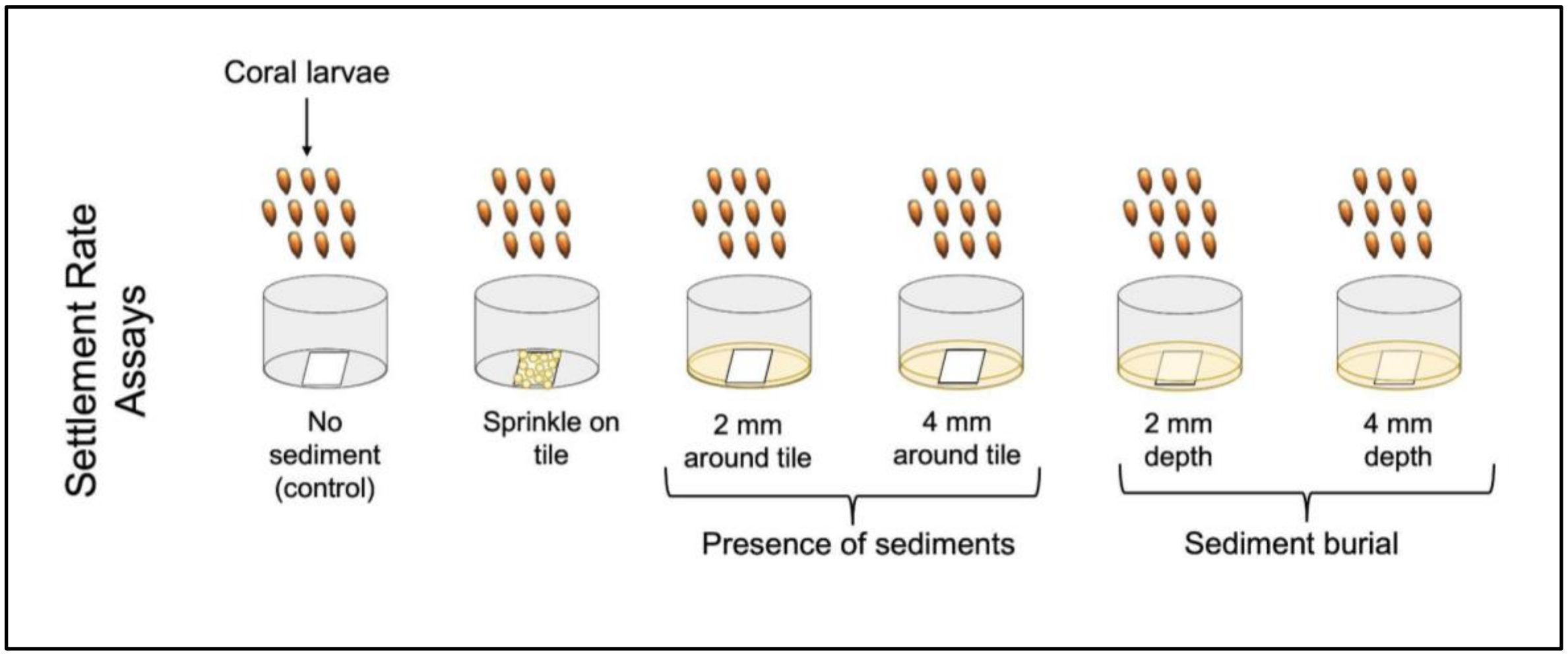

Figure S9: Schematic representation of the larval settlement assays depicting the different treatments established to test the response of larval settlement to the presence of sediments around the substrate and the burial of the substrate by sediments.

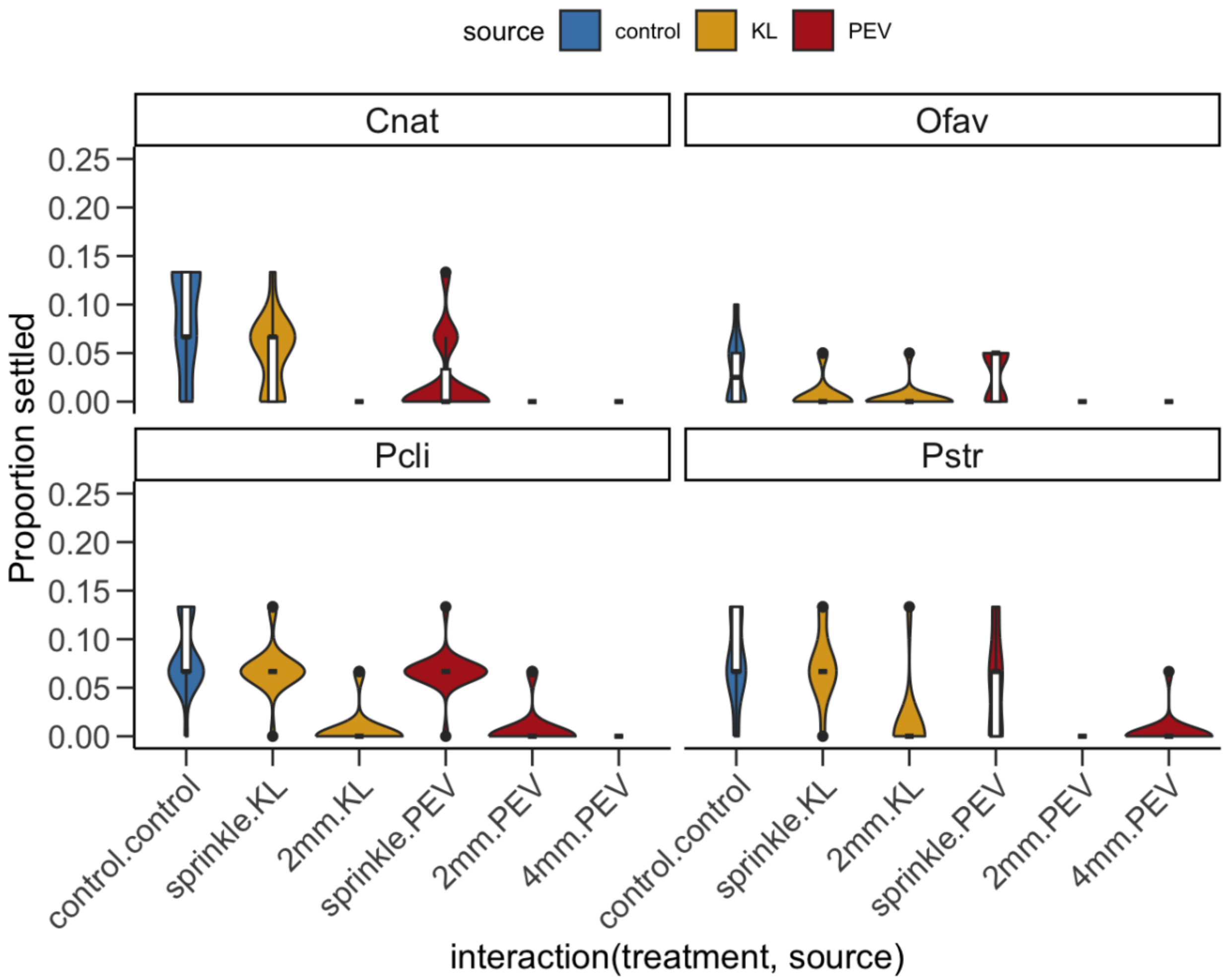

Figure S10: Box and violin plot depicting the median proportion of larval that settled on substrata that were buried under different sediment sources: KL = Key Largo; PEV = Port Everglades. Note: we did not set up any KL 4 mm treatments set up in any of the settlement assays.

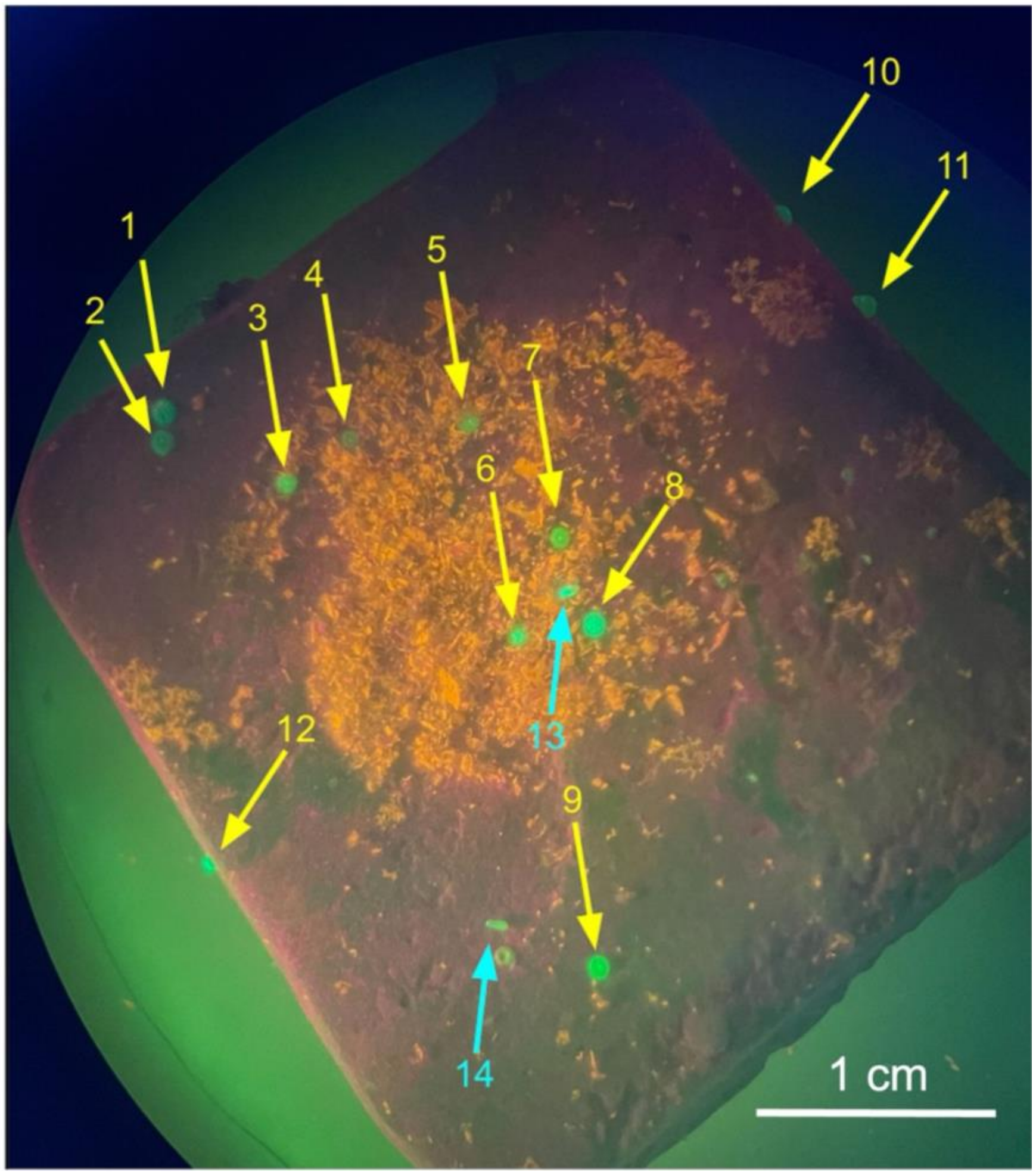

Figure S11: Photograph of a settlement tile (control treatment) after 72 hours. The fluorescent green dots are coral larvae that have settled or attached onto the tile. The yellow arrows point to larvae that have successfully settled and metamorphosed into a flat, disk-like shape to adhere to the substrate. The cyan arrows point to larvae that have firmly attached themselves to the substrate but have not yet metamorphosed. The orange areas are crushed CCA allocated to each tile to encourage coral settlement.

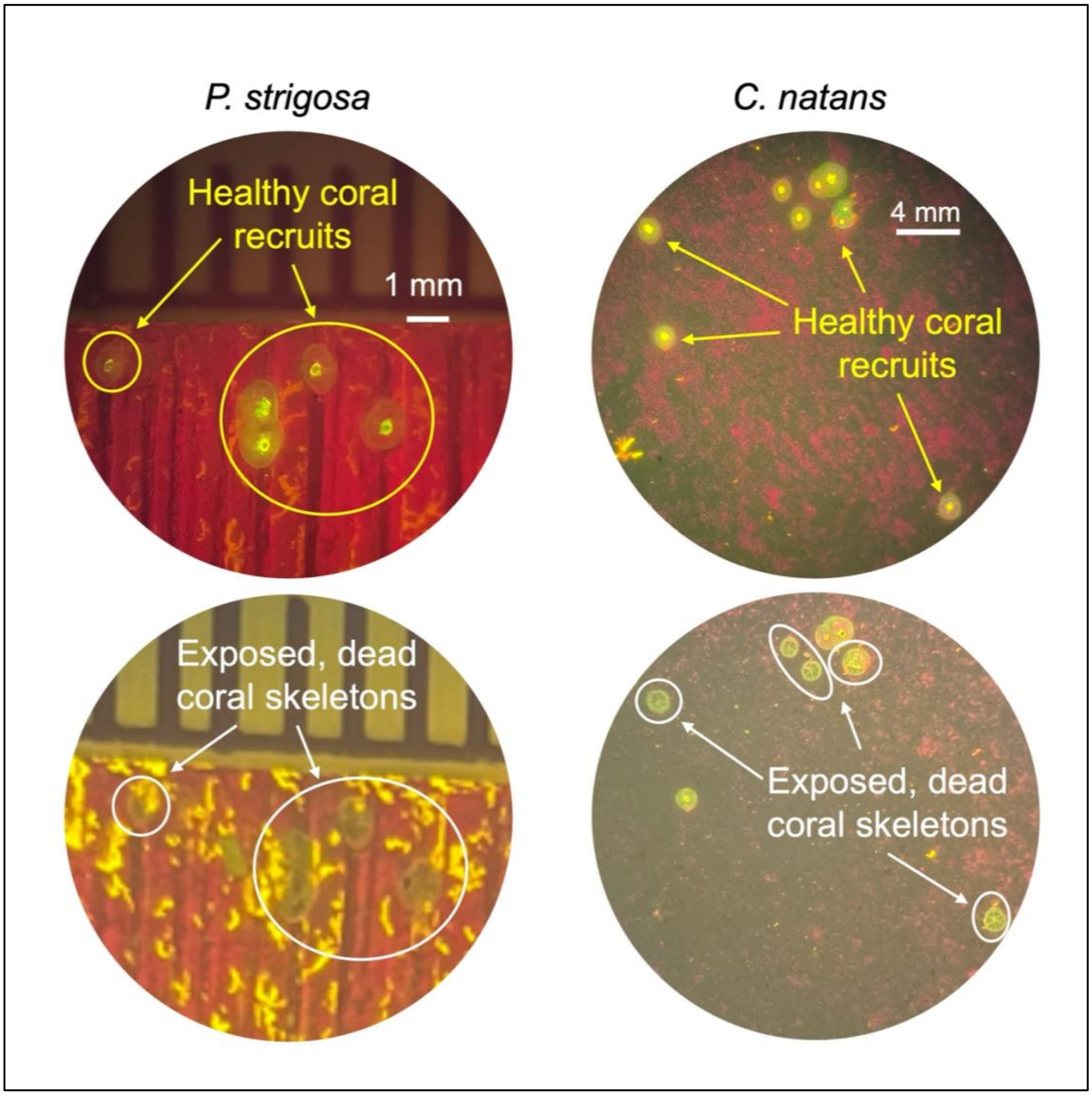

Figure S12: Photograph of 1-month-old *P. strigosa* and *C. natans* recruits before sediment burial (top) and after 10 days of burial under 4 mm of sediments (bottom). The fluorescent green polyps with live tissue represent healthy, living coral recruits and the dull, bare skeletons represent coral recruits that died after 10 days of burial.

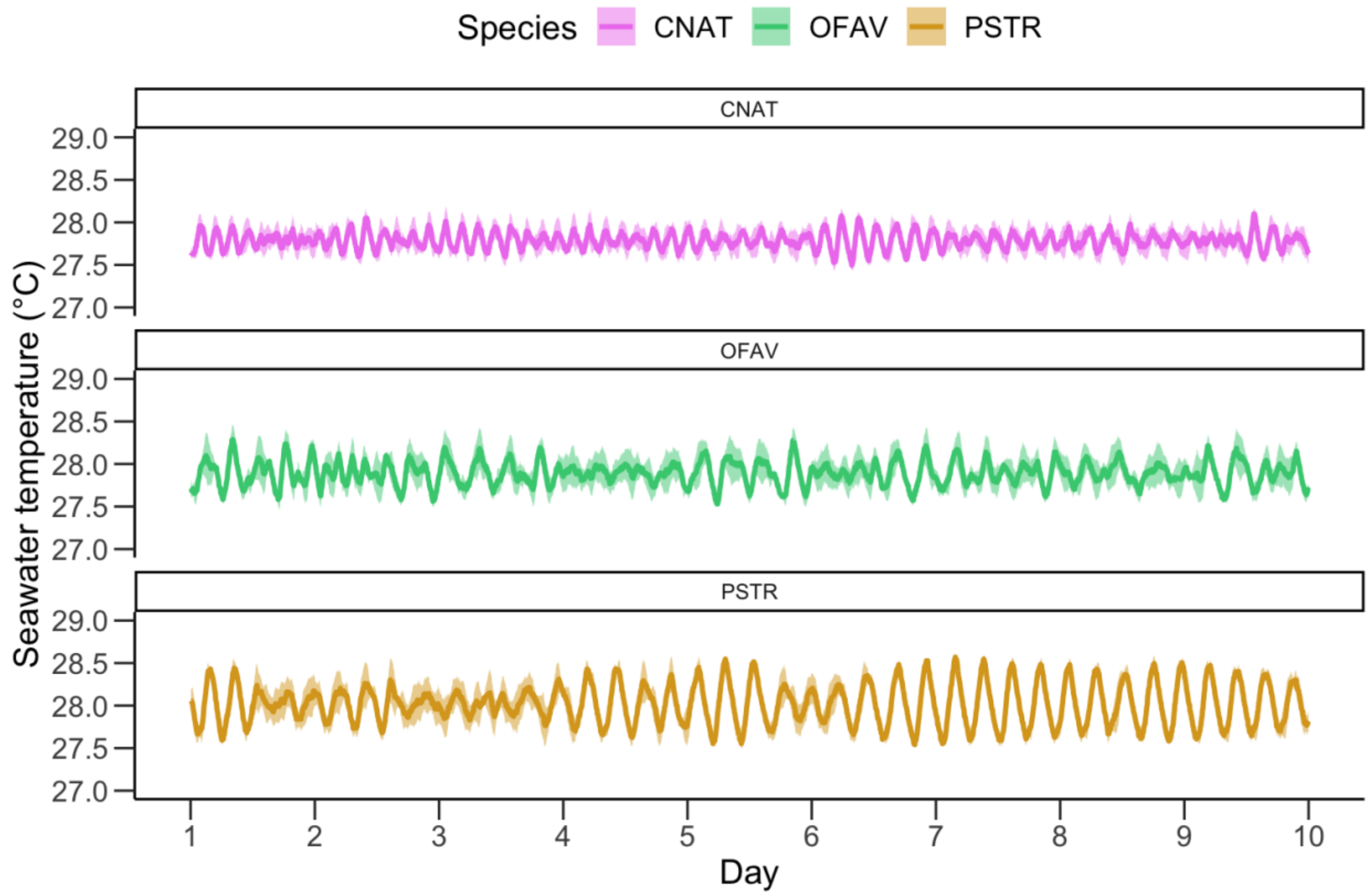

Figure S13: Mean (± SE) seawater temperature (°C) across replicates for the tanks where sedimentation exposure assays were conducted for the 1-month-old recruits of each species tested. Seawater temperature was recorded every 5 minutes using a HOBO data logger. CNAT = *Colpophyllia natans*, OFAV = *Orbicella faveolata*, PSTR = *Pseudodiploria strigosa*.

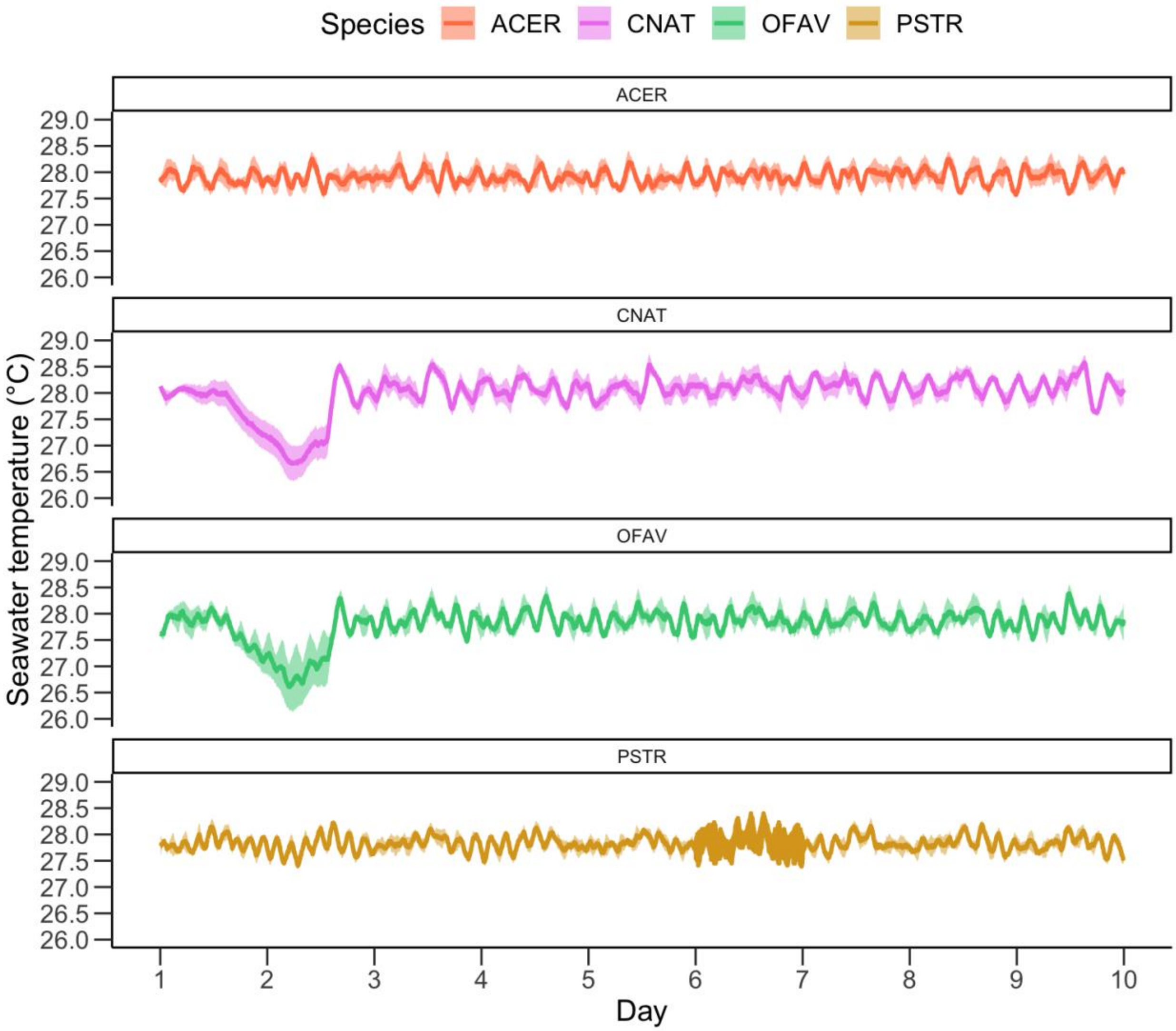

Figure S14: Mean (± SE) seawater temperature (°C) across replicates for the tanks where the sedimentation exposure assays were conducted for the 3-month-old recruits of each species tested. Seawater temperature was recorded every 5 minutes using a HOBO data logger. ACER = *Acropora cervicornis*, CNAT = *C. natans*, OFAV = *O. faveolata*, PSTR = *P. strigosa*.

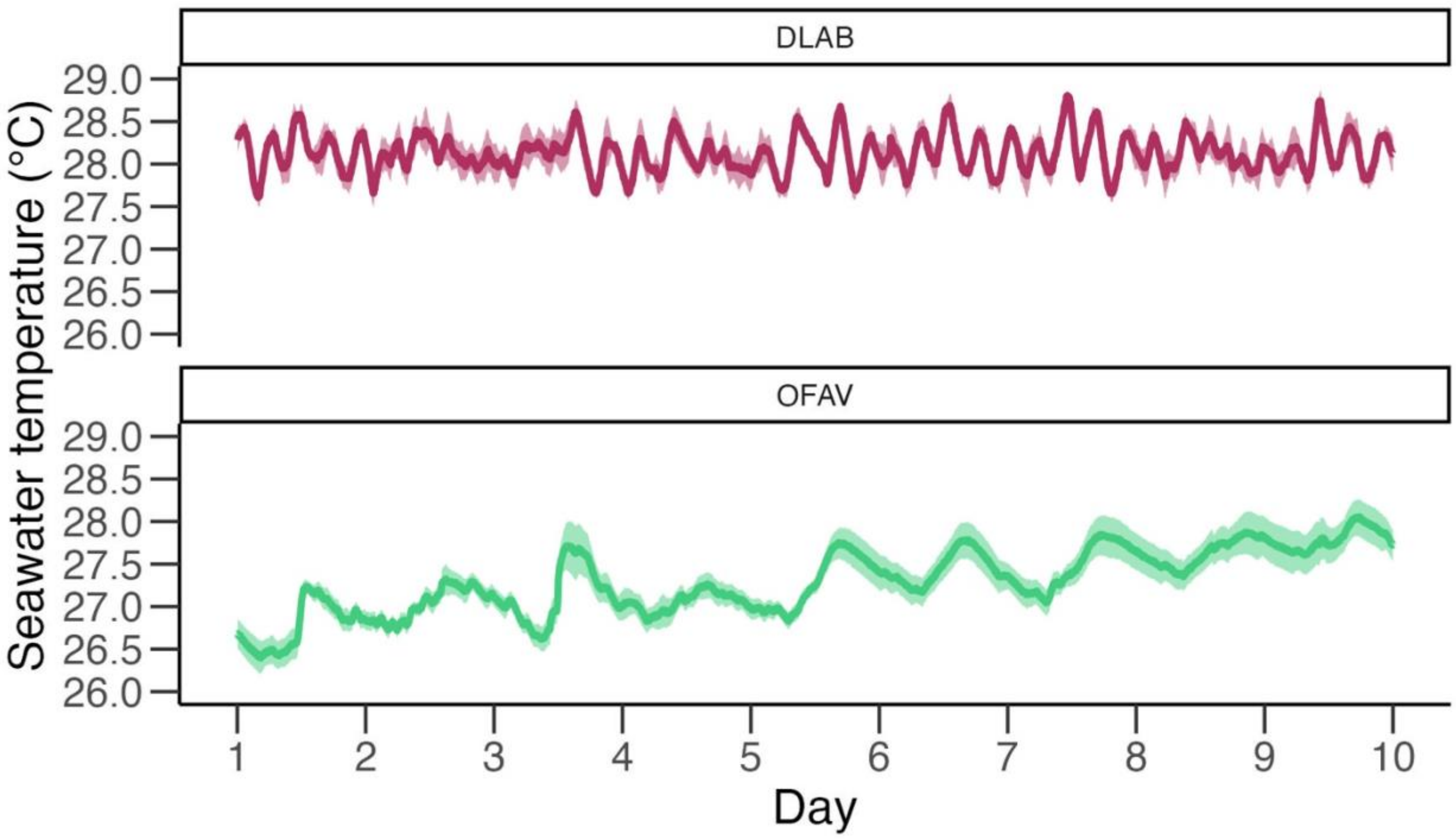

Figure S15: Mean (± SE) seawater temperature (°C) across replicates for the tanks where the sedimentation exposure assays were conducted for the 6-month-old *Diploria labyrinthiformis* recruits and the fragments of *O. faveolata* representative of 6-month-, 12-month- and 18-month-old recruits. Seawater temperature was recorded every 5 minutes using a HOBO data logger. DLAB = *D. labyrinthiformis*, OFAV = *O. faveolata*.

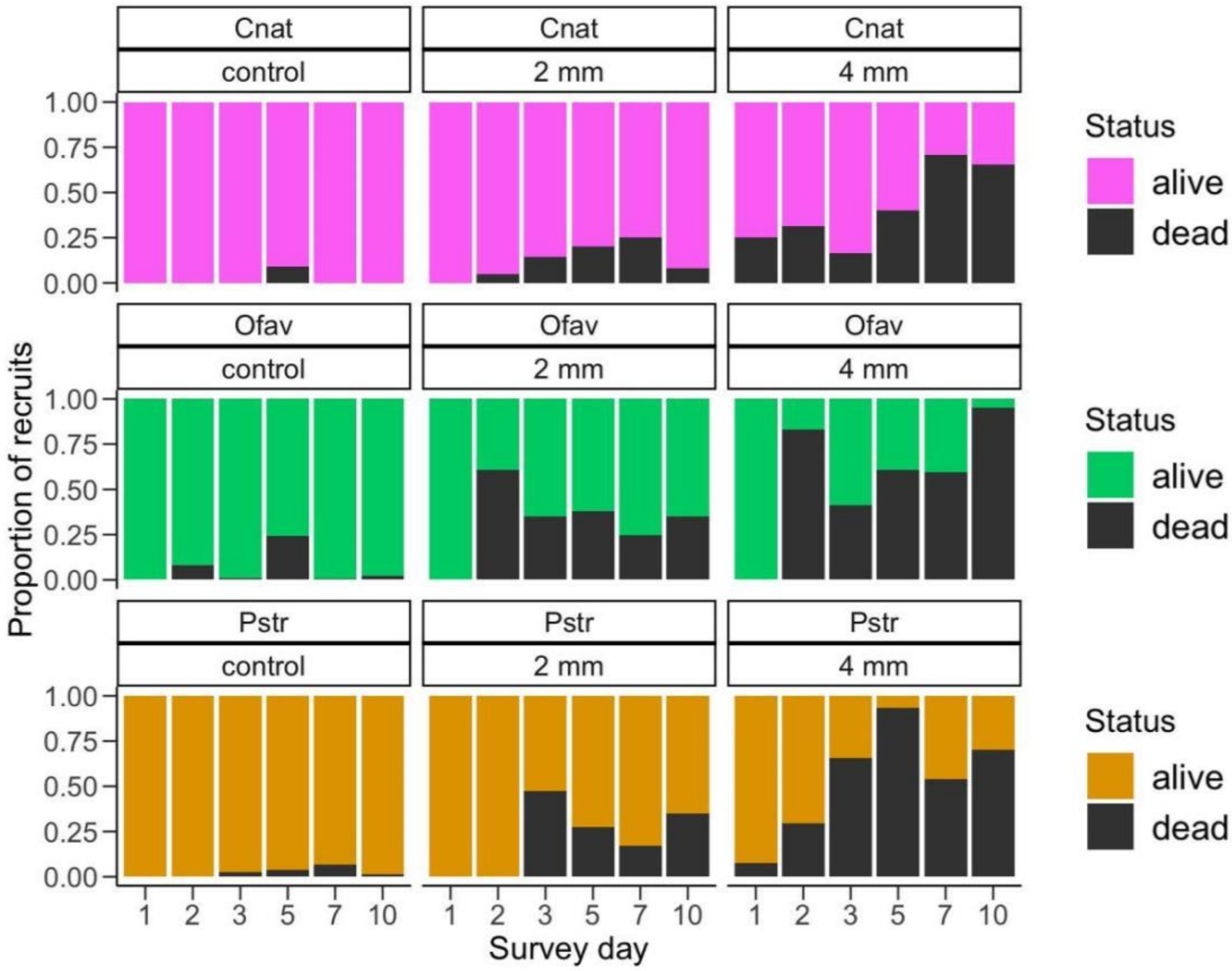

Figure S16: Stacked bar plots depicting the relative abundance of alive and dead 1-month-old recruits for each species tested across all survey days and treatments. Relative abundance was calculated across all replicates. Cnat = *C. natans*, Ofav = *O. faveolata*, Pstr = *P. strigosa*.

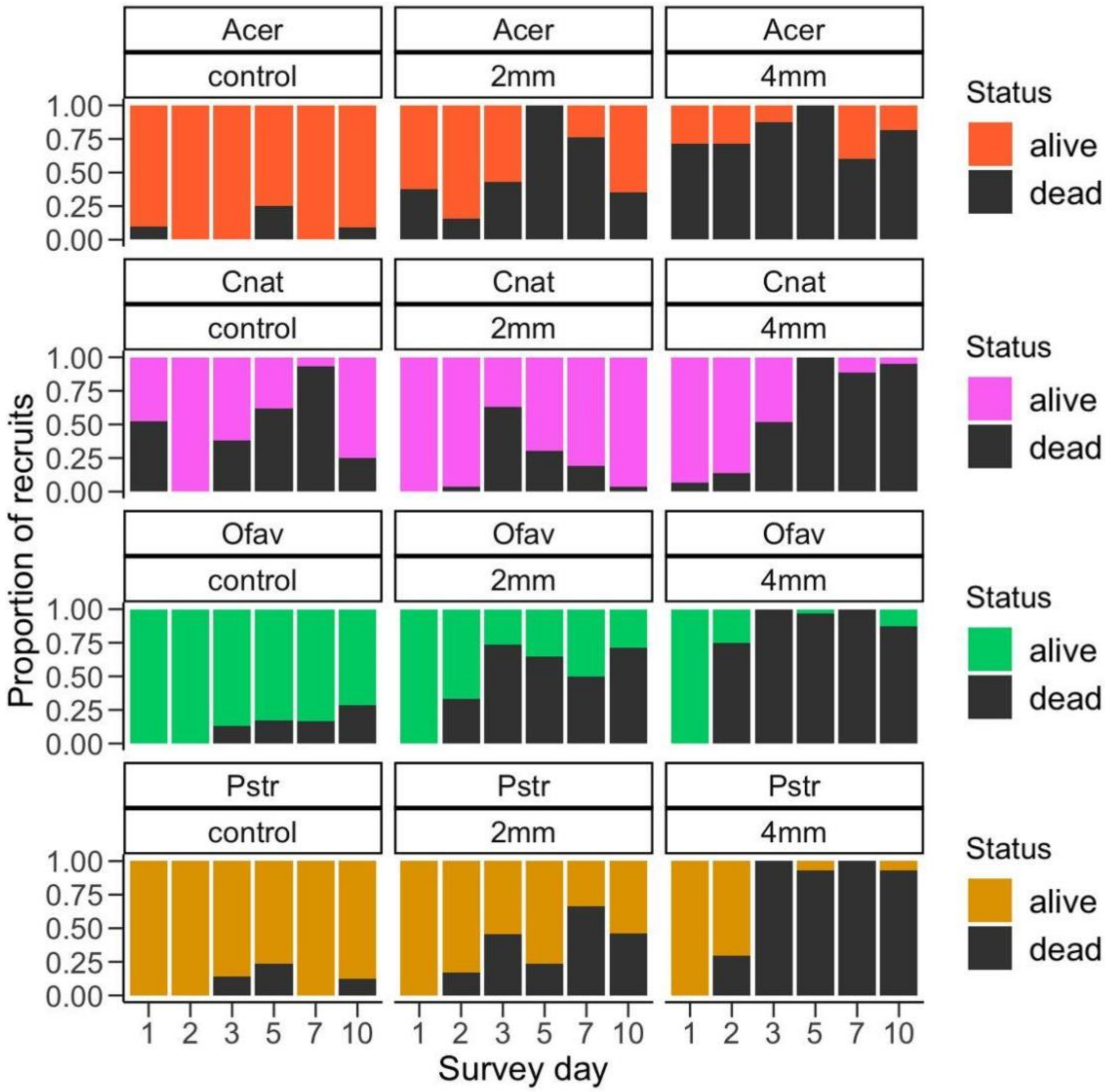

Figure S17: Stacked bar plots depicting the relative abundance of alive and dead 3-month-old recruits for each species tested across all survey days and treatments. Relative abundance was calculated across all replicates. Acer = *Acropora cervicornis*, Cnat = *C. natans*, Ofav = *O. faveolata*, Pstr = *P. strigosa*.

LITERATURE CITED

1. U.S. Army Corps of Engineers. *Appendix I: Evaluation of expected sedimentation exposure in the offshore of Port Everglades as a result of the Port Everglades, Florida project*. U.S. Army Corps of Engineers, Jacksonville District (2022). Available at: https://usace.contentdm.oclc.org/utils/getfile/collection/p16021coll7/id/19927.

2. Harper, L. M., Huebner, L. K., O'Cain, E. D., Ruzicka, R., Gleason, D. F. & Fogarty, N. D. Multi-year coral recruitment study across the Florida Reef Tract reveals boom-or-bust pattern among broadcast spawners and consistency among brooders. *Mar. Ecol. Prog. Ser.* **721**, 39–58 (2023).

3. Ritson-Williams, R., Arnold, S. N. & Paul, V. J. Patterns of larval settlement preferences and post-settlement survival for seven Caribbean corals. *Mar. Ecol. Prog. Ser.* **548**, 127–138 (2016).
