# Supplementary material for: Sediment burial reduces coral larval settlement, recruit survivorship, and inhibits the recovery of coral populations
Source: Commun Biol. 2026 Jun 17;9:1284. doi: 10.1038/s42003-026-10370-1 (PMC13635240; doi:10.1038/s42003-026-10370-1)
Supplement: Supplementary file 2 [file 42003_2026_10370_MOESM2_ESM.pdf]

nature portfolio

Corresponding author(s): Mark C Ladd

Last updated by author(s): Feb 10, 2026

Reporting Summary

Nature Portfolio wishes to improve the reproducibility of the work that we publish. This form provides structure for consistency and transparency in reporting. For further information on Nature Portfolio policies, see our Editorial Policies and the Editorial Policy Checklist.

Statistics

For all statistical analyses, confirm that the following items are present in the figure legend, table legend, main text, or Methods section.

n/a	Confirmed	
☐	☒	The exact sample size (*n*) for each experimental group/condition, given as a discrete number and unit of measurement
☐	☒	A statement on whether measurements were taken from distinct samples or whether the same sample was measured repeatedly
☐	☒	The statistical test(s) used AND whether they are one- or two-sided<br>*Only common tests should be described solely by name; describe more complex techniques in the Methods section.*
☐	☒	A description of all covariates tested
☐	☒	A description of any assumptions or corrections, such as tests of normality and adjustment for multiple comparisons
☐	☒	A full description of the statistical parameters including central tendency (e.g. means) or other basic estimates (e.g. regression coefficient) AND variation (e.g. standard deviation) or associated estimates of uncertainty (e.g. confidence intervals)
☐	☒	For null hypothesis testing, the test statistic (e.g. *F*, *t*, *r*) with confidence intervals, effect sizes, degrees of freedom and *P* value noted<br>*Give P values as exact values whenever suitable.*
☐	☒	For Bayesian analysis, information on the choice of priors and Markov chain Monte Carlo settings
☐	☒	For hierarchical and complex designs, identification of the appropriate level for tests and full reporting of outcomes
☐	☒	Estimates of effect sizes (e.g. Cohen's *d*, Pearson's *r*), indicating how they were calculated

Our web collection on statistics for biologists contains articles on many of the points above.

Software and code

Policy information about availability of computer code

Data collection	All data used in this manuscript are available at https://github.com/victorruano/Effects-of-sedimentation-on-juvenile-corals-2023-2024-
Data analysis	All R code developed to analyze the data are available at https://github.com/victorruano/Effects-of-sedimentation-on-juvenile-corals-2023-2024-

For manuscripts utilizing custom algorithms or software that are central to the research but not yet described in published literature, software must be made available to editors and reviewers. We strongly encourage code deposition in a community repository (e.g. GitHub). See the Nature Portfolio guidelines for submitting code & software for further information.

Data

Policy information about availability of data

All manuscripts must include a data availability statement. This statement should provide the following information, where applicable:

- Accession codes, unique identifiers, or web links for publicly available datasets
- A description of any restrictions on data availability
- For clinical datasets or third party data, please ensure that the statement adheres to our policy

All data used in this manuscript are available at https://github.com/victorruano/Effects-of-sedimentation-on-juvenile-corals-2023-2024-

Research involving human participants, their data, or biological material

Policy information about studies with human participants or human data. See also policy information about sex, gender (identity/presentation), and sexual orientation and race, ethnicity and racism.

Reporting on sex and gender	Not applicable
Reporting on race, ethnicity, or other socially relevant groupings	Not applicable
Population characteristics	Not applicable
Recruitment	Not applicable
Ethics oversight	Not applicable

Note that full information on the approval of the study protocol must also be provided in the manuscript.

Field-specific reporting

Please select the one below that is the best fit for your research. If you are not sure, read the appropriate sections before making your selection.

☐ Life sciences ☐ Behavioural & social sciences ☒ Ecological, evolutionary & environmental sciences

For a reference copy of the document with all sections, see nature.com/documents/nr-reporting-summary-flat.pdf

Ecological, evolutionary & environmental sciences study design

All studies must disclose on these points even when the disclosure is negative.

Study description

We conducted experimental research that assessed the response of larval settlement and recruit survival to the presence of sediments and substrate burial across multiple Caribbean species and life stages. We assessed the probability of settlement of coral larvae when sediment was present around the substrate or burying it under a "sprinkle", (0.15 g), 2 mm, and 4 mm of sediment. The burial of suitable substrate significantly decreased the settlement probability for all species tested, with just 2 mm of sediment decreasing settlement probability to 25% or less and 4 mm suppressing settlement entirely. To test how the effects of sediment burial may change with coral age, we used recruits of different ages, including 1-month-old recruits (C. natans, O. faveolata, and P. strigosa), 3-month-old recruits (A. cervicornis, C. natans, O. favelata, P. strigosa), and 6-month-old recruits (D. labyrinthiformis only). In addition, we tested the effect of sediment burial on fragments of O. faveolata approximating the size of 6-month-, 12-month- and 18-month-old recruits. This was accomplished by fragmenting established O. faveolata colonies into size classes representative of each age group: 1 x 1 cm (1 cm2) for 6-month-old recruits, 1.5 x 1.5 cm (2.3 cm2) for 12-month-old recruits, and 2 x 2 cm (4 cm2) for 18-month-old recruits. The species included at each life history stage were based on recruit availability. For these experiments, we exposed coral recruits to three treatments: a control (no sediment present), burial under 2 mm of sediment, or burial under 4 mm of sediment. To test how burial duration influences the survival of recruits, recruits were exposed to sediment burial treatments for 1, 2, 3, 5, 7, or 10 days. We used a block design to conduct the experiments in 20-gallon tanks where we deployed 6 x 3 grids of PVC chambers (5 x 5 cm each), whereby each chamber was designed to house a single tile, with each row containing the three treatments (control, 2 mm, 4 mm) per day (6 days for 3 treatments). We had four tanks per species per age group (24 tanks total), each consisting of one replicate per species per age group.

We sampled six shallow (6–8 m deep) reef sites along the Florida reef tract, from Fort Lauderdale in Broward County, USA, to Key Largo in the upper Florida Keys, USA. Our sites include Fort Lauderdale (Broward County), Dania Beach (Broward County), South Canyon (Miami Beach), Emerald Reef (Key Biscayne), Carysfort Reef (Key Largo), and Conch Reef (Key Largo; Table S4). These reefs are characterized as offshore shelf-edge reefs101, and we focused on sampling the shallow fore-reef habitat of each site (5–7 m). Each site was sampled in May of 2023. At each site, beginning at a random location, we outlined a permanent 25 m transect oriented from north to south using the surveyor's tape. We hammered metal nails at every 1-meter interval to mark all 25 plots in a site. We used a 25 x 25 cm quadrat to characterize the in-situ benthic community composition at the lowest taxonomic resolution possible. Whenever we found LSAT within the 25 x 25 cm plot, we recorded the sediment depth at five randomly selected locations. Each plot was photographed for quality control (taxa identification and percent cover estimation). Within each 25 x 25 cm quadrat, we also identified every juvenile coral (≤ 4 cm diameter) to the genus level and measured their maximum diameter (cm). The number of coral juveniles was divided by the area of the plot (0.0625 m2) to estimate the density of juvenile corals (individuals m-2).

Research sample

We collected coral larvae from reefs offshore of Key Largo, Florida. These reefs are monitored annually by our research group at NOAA's Southeast Fisheries Science Center. Coral colonies are monitored during their respective spawning windows, gametes are collected, fertilized and reared for settlement assays and coral recruit burial assays.

Sampling strategy

Sample sizes for each experiment were maximized to account for as much of the variation as possible within each experiment. The sample size in each of our experiments was constrained by personnel and resource availability. Nevertheless, our results demonstrate consistent, significant trends that accurately portray the variations of the response variables to manipulations of the predictor variables.

Data collection

All data were collected by the authors when laboratory samples were processed and in situ in the field. All data underwent QA/QC by the authors after the data was collected and during the analyses.

Settlement assays were performed in individual glass chambers placed into water baths in a random block design. Each water bath contained 30 individual chambers, such that a single replicate (n = 45 chambers) was spread across two water baths. Each chamber was assigned one of eight experimental sediment treatments or a control, which received no sediment addition. Altogether, each settlement assay consisted of nine different treatments: (1) +2.15 g of PEV sediment present, (2) +4.30 g of PEV sediment present, (3) +0.15 g of PEV sediment burial, (4) +2.15 g of PEV sediment burial, (5) +4.30 g of PEV sediment burial, (6) +0.15 g of KL sediment present, (7) +2.15 g of KL sediment present, (8) +0.15 g of KL sediment burial, (9) +2.15 g of KL sediment burial, as well as (10) controls where no sediment was present in the chamber.

For the recruit burial experiments, we exposed coral recruits to three treatments: a control (no sediment present), burial under 2 mm of sediment, or burial under 4 mm of sediment. To test how burial duration influences the survival of recruits, recruits were exposed to sediment burial treatments for 1, 2, 3, 5, 7, or 10 days. We used a block design to conduct the experiments in 20-gallon tanks where we deployed 6 x 3 grids of PVC chambers (5 x 5 cm each), whereby each chamber was designed to house a single tile, with each row containing the three treatments (control, 2 mm, 4 mm) per day (6 days for 3 treatments). We had four tanks per species per age group (24 tanks total), each consisting of one replicate per species per age group. On day 0, we counted, photographed, and mapped the location of all recruits within each tile using a dissecting microscope. The tiles were then placed into their respective chambers, and sediment was added to each one based on their assigned experimental treatment (2 mm and 4 mm) using a pipette. At each timepoint, sediment was removed from the respective tiles using a siphon, and the number of living and dead coral recruits was counted and photographed.

We sampled six shallow (6–8 m deep) reef sites along the Florida reef tract, from Fort Lauderdale in Broward County, USA, to Key Largo in the upper Florida Keys, USA. Our sites include Fort Lauderdale (Broward County), Dania Beach (Broward County), South Canyon (Miami Beach), Emerald Reef (Key Biscayne), Carysfort Reef (Key Largo), and Conch Reef (Key Largo). These reefs are characterized as offshore shelf-edge reefs, and we focused on sampling the shallow fore-reef habitat of each site (5–7 m). Each site was sampled in May of 2023. At each site, beginning at a random location, we outlined a permanent 25 m transect oriented from north to south using the surveyor's tape. We hammered metal nails at every 1-meter interval to mark all 25 plots in a site. We used a 25 x 25 cm quadrat to characterize the in-situ benthic community composition at the lowest taxonomic resolution possible. Whenever we found LSAT within the 25 x 25 cm plot, we recorded the sediment depth at five randomly selected locations. Each plot was photographed for quality control (taxa identification and percent cover estimation). Within each 25 x 25 cm quadrat, we also identified every juvenile coral (≤ 4 cm diameter) to the genus level and measured their maximum diameter (cm). The number of coral juveniles was divided by the area of the plot (0.0625 m2) to estimate the density of juvenile corals (individuals m-2).

Timing and spatial scale

All experiments were conducted during the summer of 2023. Since this is the time of the year in which most Caribbean coral species spawn, we were constrained to this time of the year due to the availability of larvae for experiments. Since coral larvae settle, on average, 72 days after they start probing for suitable substrate, settlement assays were sampled 72 hours after they were set up. Recruit burial assays were sampled across two-day intervals for ten days based on previous literature indicating the onset of detrimental effects of sediment burial on coral health. For field surveys, the transects were designed to describe the benthic composition at the reef scale (tens to hundreds of meters) across the Florida Reef Tract (tens of km).

Data exclusions

No data were excluded from this study.

Reproducibility

The experiments described in this manuscript were reproduced during the summer of 2024 an we achieved similar results. The results of the experiment are described in the final report submitted to the Florida Department of Environmental Protection on June 15, 2025, and is available upon request.

Randomization

Replicates within water baths and tanks were haphazardly placed to ensure that all treatments were spread evenly across each quadrant of the water baths and tanks to minimize the effects of covariates such as variations in light, temperature, water flow, etc. Powerheads and heaters were placed in each water bath and tanks, fluctuations in conditions were monitored using HOBO pendant data loggers.

Blinding

Not applicable

Did the study involve field work? ☒ Yes ☐ No

Field work, collection and transport

Field conditions

Field work was conducted during conditions that were conducive to safe boating and SCUBA diving operations. The main factors considered for safe field operations were wind speed (anything <12 knots) and swells (anything <3 feet).

Location

Field surveys were conducted at six sites across the Florida Reef Tract. All sites were at 5 m deep:

Fort Lauderdale (26.207320, -80.085170)
Dania Beach (26.074640, -80.096050)
South Canyon (25.849710, -80.103680)
Emerald Reef (25.678100, -80.097367)
Carysfort Reef (25.222730, -80.208750)
Conch Reef (24.961640, -80.453610)

Access & import/export

Sites were accessed by boat. When available at certain sites, the boat was attached to mooring buoys via ropes. Otherwise, the boat

Access & import/export	was anchored ensuring the anchor was placed in a sandy patch to not damage the surrounding reef environment.<br><br>Protocols were approved by the Florida Department of Environmental Protection, the Florida Keys National Marine Sanctuary (FKNMS), and the Florida Fish and Wildlife Conservation Commission (FWC). All research was conducted under permits provided by FWC (permit SAL-23-2526-SRP) and FKNMS (permits FKNMS-2018-163-A4 and FKNMS-2023-021).
Disturbance	Masonry nails were hammered into the reef framework to delineate the transects for resampling during subsequent seasons. We ensured that no nails were hammered into living corals or other benthic animals.

Reporting for specific materials, systems and methods

We require information from authors about some types of materials, experimental systems and methods used in many studies. Here, indicate whether each material, system or method listed is relevant to your study. If you are not sure if a list item applies to your research, read the appropriate section before selecting a response.

Materials & experimental systems

n/a	Involved in the study
☒	☐ Antibodies
☒	☐ Eukaryotic cell lines
☒	☐ Palaeontology and archaeology
☐	☒ Animals and other organisms
☒	☐ Clinical data
☒	☐ Dual use research of concern
☒	☐ Plants

Methods

n/a	Involved in the study
☒	☐ ChIP-seq
☒	☐ Flow cytometry
☒	☐ MRI-based neuroimaging

Animals and other research organisms

Policy information about studies involving animals; ARRIVE guidelines recommended for reporting animal research, and Sex and Gender in Research

Laboratory animals	Six species were used in this study: Acropora cervicornis, Colpophyllia natans, Orbicella faveolata, Pseudodiploria clivosa, Pseudodiploria strigosa, and Diploria labyrinthiformis. We used individuals that were 1-month, 3-months, 6-months, 12 months, and 18-months old.
Wild animals	Gametes were collected from the field by placing a spawning net on the colony and accumulating gamete bundles in a collection tube attached at the top of the net. The top of the net consists of a plastic transmission funnel, the mouth of which leads to a 50ml centrifuge tube screwed onto a permanently attached cap at the top of the net (Figure 1). The cap has a hole drilled in the center that is placed over the narrow end of the funnel allowing gamete bundles to pass through while retaining the threaded sides for attaching and swapping centrifuge tubes.<br><br>Gametes were transported to NOAA's Southeast Fisheres Science Center in Miami, Florida, where they were fertilized and reared to the larval stage. Larvae were used for settlement assays. Those that successfully settled on ceramic tiles from the experiment were kept and reared in tanks. They were eventually grown out into coral colonies. Once they achieved a significant size (>5 cm), they were outplanted onto reefs undergoing restoration activities.<br><br>Larvae that were not used in settlement assays were reared to recruits and used for the recruit burial assays. Those that survived were reared in the hatchery into coral colonies destined for outplanting in future restoration efforts.
Reporting on sex	All species tested are hermaphroditic broadcast spawners. All results apply equally to all individuals tested.
Field-collected samples	All larvae and corals collected and used for experimentation were subjected to water temperatures between 27.5 and 28.5 °C and a 12-hour photoperiod (07:00–19:30). Recruits were housed in 20-gallon aquarium tanks with 20-gallon sumps fed filtered (20-micron) and UV-sterilized seawater with a turnover rate of ~6x per day. Lighting was consistent for each cohort via Radion XR30 G6 Pro LED aquarium lights (EcoTech Marine, PA USA), and the temperature was kept consistent at ~28 °C using 300W titanium heaters (Finnex TH-303005 titanium heater, ISK Merchandising Inc. USA) controlled by an Apex Neptune controller system.
Ethics oversight	Protocols were approved by the Florida Department of Environmental Protection, the Florida Keys National Marine Sanctuary, and the Florida Fish and Wildlife Conservation Commission. All research was conducted under permits provided by the Florida Fish and Wildlife Conservation Commission (SAL-23-2526-SRP) and the Florida Keys National Marine Sanctuary (permits FKNMS-2018-163-A4 and FKNMS-2023-021).

Note that full information on the approval of the study protocol must also be provided in the manuscript.

Plants

Seed stocks	No plants were used for this study.
Novel plant genotypes	Not applicable.
Authentication	Not applicable.
