# Supplementary material for: Sediment burial reduces coral larval settlement, recruit survivorship, and inhibits the recovery of coral populations
Source: Commun Biol. 2026 Jun 17;9:1284. doi: 10.1038/s42003-026-10370-1 (PMC13635240; doi:10.1038/s42003-026-10370-1)
Supplement: Supplementary file 3 [file 42003_2026_10370_MOESM3_ESM.pdf]

nature portfolio

Peer Review File

Sediment burial reduces coral larval settlement, recruit survivorship, and inhibits the recovery of coral populations

Corresponding Author: Dr Mark Ladd

This manuscript has been previously submitted at another journal. This document only contains information relating to versions considered at Communications Biology.

This file contains all reviewer reports in order by version, followed by all author rebuttals in order by version.

Version 0:

Reviewer comments:

Reviewer #1

(Remarks to the Author)
Rodriguez-Ruano et al. investigate how sediment presence, burial depth, and burial duration affect coral larval settlement and recruit survivorship across several Caribbean species and life stages. They combine aquaria experiments with field surveys in Florida. The key findings are that even shallow burial layers significantly reduce settlement and survival across species, and that the presence of sediment in chambers reduces settlement in some taxa. They also run hazard models to simulate how multiple sediment events could impact recruit cohorts, concluding that sedimentation is a major bottleneck for coral population recovery.

This manuscript addresses an understudied aspect of coral ecology: the impact of sediment burial depth and duration on larval settlement and early recruit survivorship. The experimental work is valuable and relevant, but the paper is weakened by sensationalist language, occasional misrepresentation of context, and a lack of clarity in how results are reported. Cross-species comparisons are implied without statistical testing (that I can see), uncertainty is not reported for model predictions or hazard ratios, and the deterministic presentation of scenario modelling is problematic. Methods also need clearer justification, especially regarding sediment definitions and conversions.

Comments:

Line 60–64: The description of Bonaire's reef trajectory overstates Steneck et al. (2019). That study shows coral cover declined from ~45% to ~35% (a 22 percentage-point drop), not a halving. While parrotfish biomass was unusually high, the relationship between herbivory and macroalgal decline was correlative, not experimentally demonstrated. Also note that macroalgal cover at the final survey remained higher than at baseline. Reword to reflect these nuances and avoid over sensationalism (see Bruno et al., 2019).

Line 71–73: Consider acknowledging the increasing role of marine heatwaves in shaping coral recovery.

Line 96–100: The term "paucity" overstates the literature gap. Soften by acknowledging existing studies and clarifying that the gap lies in multi-species, multi-stage Caribbean work.

Line 100: Typographical issue: ".Surprisingly" should read ". Surprisingly".

Line 103: Please acknowledge dredging and sediment burial studies, even if not controlled laboratory experiments.

Line 107–112: The contrast between Caribbean and Indo-Pacific recovery should also note life history differences: Indo-Pacific reefs retain fast-growing branching taxa that recover rapidly, whereas Caribbean assemblages are dominated by slower-growing massive species and fewer opportunists. Include this alongside regional stressor context.

Line 109–112: Repeat bleaching events, driven by global climate change, are a dominant driver of mortality and must be acknowledged to avoid overstating sediment impacts.

Line 113–114: Rephrase too sweeping.

Line 149–151: Phrases such as "appeared to be the most resistant" are vague. If species-level differences are claimed, statistical tests are required. Otherwise, describe trends without implying significance. Your stated aim was to examine interspecific variation, but no direct comparisons are shown.

Results: No errors or confidence intervals are reported for settlement probabilities or hazard ratios. These should be included.

Results (Field surveys): Clear and well presented.

Figure 1g and 1i: For consistency, please show model fits.

Line 253: The statement on sediment stress is overly simplistic. Sediment impacts are multifaceted and context dependent.

Line 368–383: The scenario modelling reports numerical outcomes and reads as results?

Line 390–395: Avoid absolute language such as "sufficient to kill" and "plummets."

Line 440–452: Clarify how mass was converted to both mg cm^{-2} and mm burial depth. These conversions depend on tile area and sediment density, which should be stated.

Line 463–464: If combining sediment sources, please report the statistical test used to conclude "no observed difference."

General: Define what is meant by "sediment" in this study. Was grain size distribution, composition, or organic content quantified? Since these factors strongly affect burial and survivorship, they should be reported for the experimental sediments, not only in field surveys.

Reviewer #2

(Remarks to the Author)
I commend the authors on a well-designed and executed set of experiments! The paper is a nice contribution to the state of knowledge on the potential impacts of excessive reef sediments on the success of coral larvae in finding a suitable place to settle and survive as young recruits when buried by reef sediments. These are important topics in the discipline of coral reef science, as researchers and managers around the word seek solutions to aid coral reef recovery from mortality events caused by escalating ocean temperatures, diseases, etc. Outstanding features of the study are that five species of corals at various life stages were tested, including 2 that are threatened species, and all are important habitat-forming species essential for reef-ecosystem functioning. It is particularly informative to know that there are species-specific responses, indicating that some species are more at risk to excessive sediments than others. I have only minor suggestions for additional background information, clarity, and interpretation, which I include below.

Title: The word "sedimentation" implies a rate (amount per unit time), so I suggest using "Excessive sediments may reduce…" and then fix subject-verb agreement so "Excessive sediments may reduce larval settlement, recruit survivorship, and the recovery of coral populations"

Abstract: line 30, state early that this was an aquarium-based study with supporting field surveys. Last sentence is a little vague and yet too strong at the same time. I think your survey results suggest that the availability of suitable coral-settlement habitat is currently low in Florida, at least in the upper Keys, and the experiments showed that treatments simulating the accumulation of sediments on the reef substratum decreased coral settlement and recruit survival of 5 important habitat-forming species. The implications for managers section of the discussion needs a little work, so conclusion statement of abstract may need to be adjusted accordingly.

Introduction: Nicely written and well-cited. A couple little things.
Line 73, re: the two papers cited for "overgrowth by competitors," citation 35 doesn't document overgrowth, and instead suggests possible allelopathy, but citation 36 does encompass general algal-grazing-recruitment processes that could include overgrowth. This could be fixed easily by adding "allelopathy and/or overgrowth by competitors" to that sentence.
Line 84, What about type of source material? Grain size?

Line 87, re: “paucity of Caribbean data,” See this pioneering study with caged and uncaged settlement blocks from the early 70s in San Blas Islands, Caribbean side of Panama: Birkeland, C. 1977. The importance of rate of biomass accumulation in early successional stages of benthic communities to the survival of coral recruits. Pages 15-21 in Proceedings of the 3rd International Coral Reef Symposium, Miami. Paraphrasing from their study: "Mortality of recruits on upper surfaces was caused by the small corals being smothered in sediment. The filamentous algae did not directly overgrow the coral but created a sediment trap."
Line 129 and throughout paper, the word “substrates” is an awkward one that has been misused for decades. Enzymes work on substrates, and the bottom of the ocean is more properly the “substratum” (substrata = plural). I understand that the coral science literature has adopted the word substrate, but the use of it in the sentence stuck out to me as more odd than usual. How about “…the burial of the substratum and corals…”
Line 140, “the need for avoiding and minimizing sediment sources,” I suggest adding the word “anthropogenic,” so “minimizing anthropogenic sediment sources.” Managers will have little control over natural sediment-transport processes, and I have added comments below for the discussion to add some attention to the literature on these processes. Sediment resuspension from periodic wind/wave events can be a significant driver of LSAT-sediment depth, and the passage of hurricanes can also cause redistribution and un-compaction of soft sediments.

Results:
Line 146, suggest “burial of potential settlement substratum.”
Line 150 & 164, this a cool result about DLAB and the other species-specific responses! The results mirror habitat conditions where the species are found, like DLAB and PCLI having high abundances in the back-reef and lagoon areas where soft sediments are often deep, and reservoirs are nearby.
Line 167, the “water quality” data and interpretations that result are my one main complaint about the aquarium experiments. The static (unstirred, I believe?) conditions inside the small vessels where the treatments were applied do not replicate the reef environment well, when it comes to exposure to and flux rates of nutrients. These were also sterilized sediments lacking the microbiome that remineralizes organics and drive N and P cycling on reefs. Please add some caveats about “water quality” and water-flow conditions in the experiments not being like those experienced on reefs to the discussion. Line 168, “high” concentrations of nitrate…how high were they? Please report mean and/or maximum measurements here. Also, Table 3 is not informative. Please change the table to include the raw data values measured in SI units.
Line 224, during what season were the surveys conducted in, and was the seasonal timing roughly the same? LSAT depth and turf-algal growth could be affected by season (e.g., wind, rain, wave conditions). Also, did these field observations drive the treatment levels for the sea-table experiments? It would be good, if they were, to justify treatment levels. If so, then maybe flip the order of how these pieces are presented in the methods and results.
Line 227, do you think the variability was because of differential sampling times? Were there any timeseries data to back-up temporal persistence of the spatial patterns observed? Any previously published data to support these?

Discussion:
Line 257, “changes” in water quality across treatments, do you mean differences among treatments? “Changes” imply timeseries data. Also, these differences are related to there being no water motion in the chambers, which is not very realistic. Maybe not in the second sentence, but somewhere here would be a good place to add the caveats about water motion in the vessels.
Line 264, “all but for one species tested.” Specify by name, DLAB, here because this is an important result!
Lines 286 – 289, as I mentioned above, LSAT and amounts and depths of sediments trapped could vary seasonally, with wind events creating windows of sediment resuspension and transport. I’ve observed and heard likewise from others who are in the field a lot, that strong hurricanes leave the reef sediments less compacted (i.e., “loose”, where you can easily stick your arm up to your elbow in the sand). I’m curious if observations like this have been measured or published. This could be a possible mechanism for the reported overall trend of declining (or less accessible) suitable recruitment habitat on Florida reefs. Bigger hurricanes could be allowing more frequent and greater magnitude sediment-resuspension events. See Yates et al. https://doi.org/10.5194/os-20-661-2024. Another aspect of LSAT that isn’t really mentioned is the turf-algal component. How has that changed over time? Are turfs growing taller than they used to (less urchin grazing) and catching more sediments, or did increase in sediments allow turfs to grow longer? There is potential for a positive feedback loop here.
Line 335, “contrary to our hypothesis…” Yes, but not that is unsurprising since bigger recruits respire more and would suffocate in these artificially still conditions. Could happen during large sediment redistribution event (hurricane), but on a day to day basis, I’d think most reefs are well flushed enough to keep that suffocation from happening for 10 days straight.
Line 364, these results are likewise unsurprising, probably hypoxia. Effects of dissolved oxygen on coral early life stages deserves a sentence somewhere in here. See for example, https://doi.org/10.3389/fmars.2023.1254965. That reminds me, do you have any DO data from the experiments? People tend to overlook DO, but it can cause major issues if it drops below 2 mg/L, and in small, closed vessels, this is not unlikely at night if biomass is high.

Conclusion:
Line 392, There's really no discussion of if there is a significant increase in sediment supply on Florida's or other reefs, but this is implied here. If this has been documented, please cite papers. Increased rates of erosion of carbonate framework as corals die maybe? See Lidz & Hallock, 2000, https://www.jstor.org/stable/4300079.
Line 396, “continued sedimentation,” From what source, are you implying terrigenous input? or dredging? Similar to my comment above, there was a school of thought among geologists in the last century that reefs in subtropical latitudes were just in a different spot in their trajectory (senescence) because of SLR and slowing down of reef accretion and an increasing pace of erosion. See NEUMANN, A.C. and MACINTYRE, I.G., 1985. Response to sea level rise: keep-up, catch-up, or give-up. Proceedings of the 5th International Coral Reef Symposium (Papeete, Tahiti). Volume 3, pp. 105–110. This is where the idea that reefs can be "shot in the back by their own lagoons" came from, meaning that reefs get choked out or clogged up by sediments when they run out of accommodation space or start to die and degrade for whatever reason.

Line 298, same issue here. If sediment loads are from the back-reefs themselves, during resuspension events, then what could managers possibly do?
Line 401, alternatively, managers could see that assisted-reproduction coral recruits were outplanted on reef topological highs that are better flushed and less likely to accumulate autochthonous sediments.

Methods:
Line 420, the paper is very repetitive in places. Goals are stated numerous times.
Line 443, more details are needed about the settlement tiles. What were they made up, and were they conditioned in any way? Were the undersides available to larvae?
Line 445, what about grain size and size uniformity, was this kept constant?
Line 462, sediments were sieved, but were they sorted by grain size? If so, what fractions were used? Was this silt or sand?
Line 485, Was there any stirring or mixing? Not very realistic conditions, if none.
Lines 508 – 513, please report the detection limits of these instruments and analyses. Were they able to measure micromolar levels of these water quality metrics (nutrients were very low, no doubt).
Line 562, state when (dates) these surveys occurred for each location.
Lines 569 and 573, were sites and spots to measure LSAT truly randomly selected, or was it haphazard? Sounds haphazard. If truly random, include description how assigned (e.g., using a random numbers table, roll of dice, coin flips, etc.).
Line 591, was this the same 14 day period for all sites? If not, I'm sure there'd be a lot of temporal variability depending on wind and waves.
Statistics section: State upfront what statistics software was used, and if R, what packages. How were assumptions of the models evaluated? These are stated for some tests, but not others.

Figures and Tables:
Fig. 1. State what the data points represent. Each point represents a single vessel or is a mean of replicate vessels in the same treatment? I understand that the statistical models were chosen to deal with high variability, but could the raw data points (proportion of larvae settling with each vessel) still be shown on the plots? The binary response can still be used for statistics, but it would be more interesting to see where the data fall.
Supp. Material:
Table S3: I'd rather see the raw data values in SI units so we can judge for ourselves the ecological relevance of the studies (are these WQ measurements within normal ranges reported in the field?). What are the units reported for "average" column? Negative numbers suggest these are not uM values. If data are reported in SI units, then just the p-values of these contrasts could be included. These other stats numbers are not useful.

I hope you find my comments helpful and constructive! I enjoyed reading the paper and it hits on a lot of important processes in the recovery (or not) of FL reefs.

Reviewer #3

(Remarks to the Author)
Overarching comments
Well written and engaging overall, congratulations to the authors. A relatively straightforward study confirming negative effects of sediment on larval settlement and survival through early life stages in six Caribbean coral species. Findings are consistent with studies in other regions although not well documented in Florida. The study makes useful links to water quality management and will provide an important contribution to the literature on the effects of sediment load on coral recruitment in Floridian reefs.

I have some minor editorial suggestions, noted below in Specific Comments. An overall comment is that the manuscript is very long given the study - while good - is fairly straightforward. This comprehensive approach is appropriate in a thesis chapter where all the extras are needed to satisfy examiners, but for publication, it would benefit from some trimming to reduce wordage and thus improve readability and impact. This would be particularly beneficial in the methods and results sections.

The introduction and discussion are well reasoned, with good connections to water quality management challenges. However, some discussion paragraphs repeat results in more detail than necessary. These would be improved by a sentence or two focusing on the main finding of interest for comparison with other studies, rather comparing direct results.

Specific comments

Abstract
Overall nice abstract, concise and clear.

L29 – "…there is a surprising lack of information…" Suggest replacing "surprising" with "limited". Whether or not something is surprising depends on the point of view.

L31-32 – "…across multiple Caribbean species…" quantifying this number would be more informative, suggesting replacing "multiple" with "six".

Introduction
Overall solid intro, well written and sets the scene effectively. Clearly articulates the management relevance of the study.

L46 – “coral-killing” might be a bit strong. Declines are also caused by disturbances that limit growth and disrupt reproduction, without necessarily causing direct mortatily.

L48-52 – Great summarising sentence.

L52 – “Recruitment, survival and growth into reproductive adults…” Suggest replace “adults” with “maturity”. However, this sentence is a little clunky. Arguably, recruitment IS growth to reproductive maturity, so this may need to be recast depending on how the authors define recruit/recruitment (more comments on that later).

L56 – “…multitude of factors…” there’s a figure in Randall et al 2020 that summarises these neatly, might be a good citation here (Sexual production of corals for reef restoration in the Anthropocene, in MEPS).

L100 – “Surprisingly” again, suggests delete, for same reasons as L29.

L109 – Suggest replacing “futile” with “poor”.

L131 – “Surprisingly” again! Not sure it is good fit here either. The study findings are definitely interesting, but are they really surprising? They sound generally consistent with the predictions in lines 125-127. Suggest delete.

Results
Overall, nicely written, as is the whole paper, but very long and would benefit from tightening up to give greater focus on the interesting results.

L172 – Typo at end of line “the the”.

Discussion
L257 - “… the effects of sediment burial…” Should this be “sediment burial of substrates”? Suggest clarifying. Parts of this sentence seem repetitive, plus it is very long and would be more readable if made into two.

L260 – Not sure this is “surprising”, but it does contradict the phrase preceding. Please review.

L269 – At the end of this line it implies the findings are applicable to all species, which is an overreach. Suggest replacing “a coral’s” with “these coral species” or something similar. Given dominant species found in field (Siderastrea and Porites) were not among those investigated in this study, consequences of sediments may not be as pervsive for them.

L281 – Instead of “… induce severe declines or inhibition of…”, suggest just say “Inhibit”.

L289 – Might be a local way of saying this, but consider making the word “reef” after Florida’s plural?

L290 – why is it “surprising” that field surveys corroborated lab observations?

L300 – “bottlenecks” might be used out of context here, suggest “inhibition” might be more appropriate.

L318-320 – Good point well made.

L325-329 - Repeating results here and throughout this para. Would be more concise (and therefore compelling) just to strongly summarise the key finding for each point you're making.

L353-354 – Nice way to contrast different findings. It’s helpfully complementary to have two different sediment studies investigating alternate sediment deposition scenarios and finding different outcomes. Lots more to learn in this space.

L368-383 - Suggest toning down some of the modelled assertions, the reasoning is sound, but still speculative where they extend beyond what was explicitly measured in this study.

L372 – mention of “coral recruits”, given this term is not used uniformly across coral literature and scientists, please clarify your definition in the Methods and then ensure you use that consistently throughout. Some more comments on this below.

Conclusion
Really nice conclusion summarising this neat and interesting study.

L399 – Suggest not using “bottlenecks”, it doesn’t fit well here. “inhibitors” may be an alternative to consider.

L403 – Great to link this work to management priorities, however it's an overreach to imply that improved sediment management could "increase" survival and population recovery, as other external factors could override that. Suggest replacing with "improve" or something similar.

Methods
L429 – "Settlement assays were performed in individual glass chambers" Can a different word or alternate phrasing be found for this? Strictly speaking, "individual" could mean individual larva, but there were 15-20 larvae per assay. As a suggestion for authors to consider, perhaps "Each assay was performed in individual glass chambers..." ??

L445 – reference to Fig S5. Not clear on the direct relevance of this figure here? If authors are implying sediment loads were selected for correlation with sediments loads associated with LSAT across field sites, it would be helpful to articulate that plainly.

L479 – "After establishing sediment treatments…" Please clarify how long the sediments had to settle before larvae were introduced. This is relevant to the interesting comparison with another sediment study at L353.

L515 - This is relevant to the whole manuscript. It would be worth defining what you mean by "recruit" either here (or somewhere). There's not a clear definition of what stage "recruit" refers to in corals. Some use it to mean corals that have finished the process of recruiting to the reproductive adult population, others use to for corals over 2 months of age, others use it for juvenile corals <5cm diameter. Peter Edmunds gives a useful summary of this in his 2023 review in Biological Reviews (page 1864). https://onlinelibrary.wiley.com/doi/10.1111/brv.12987

L525 – on species recruit availability: This is a helpful clarification. Please also provide a short explanation of where these recruits came from and their health status prior to the burial treatment (presumably they weren't the survivors from the settlement assays??).

L548 – on temperature consistency at ~28oC, except the figures show drops in temperature on Day 2 for CNAT and OFAV. Perhaps provide brief reasoning if this was not deemed significant.

L553 - Instead of "born", maybe "spawned"? Don't think "born" is appropriate term where broadcast spawning species are included.

L554 – Maybe a typo, should there be a comma between 2024 and coral?

Tables and Figures
Well presented and east to interpret,

Supplementary material
No comments. The supplementary figures are helpful.

Version 1:

Reviewer comments:

Reviewer #1

(Remarks to the Author)
Rodriguez-Ruano et al presents a clear assessment of how sediment burial affects coral larval settlement and early recruit survivorship across multiple Caribbean coral species. The study combines controlled laboratory experiments with supporting field observations, which together provide a robust evaluation of sedimentation as a bottleneck to coral population recovery.The experimental design is sound, the statistical analyses are appropriate, and the manuscript is clearly written and balanced throughout. The results are presented logically, and the conclusions are supported by the data. I did not identify any further issues that require revision. The manuscript is suitable for publication in its current form.

Recommendation: Accept

Reviewer #2

(Remarks to the Author)
The authors addressed all of my concerns in their revision. I do not have any further recommendations for improvement.

Reviewer #3

(Remarks to the Author)
The authors have done an admirable job of addressing detailed comments from all three reviewers. I feel satisfied that my

specific comments have been appropriately handled.

In general, I still find the paper over long, particularly the methods and results sections. I do recognise it is difficult to achieve brevity when there are numerous comments to address, but there remain sections that could be tightened up, depending on the editor's preference.

However, I don't think the length is a critical problem, probably more a personal preference. I support publication of this MS and think it will be a worthwhile and useful addition to the literature.

Open Access This Peer Review File is licensed under a Creative Commons Attribution 4.0 International License, which permits use, sharing, adaptation, distribution and reproduction in any medium or format, as long as you give appropriate credit to the original author(s) and the source, provide a link to the Creative Commons license, and indicate if changes were made.
In cases where reviewers are anonymous, credit should be given to 'Anonymous Referee' and the source.
The images or other third party material in this Peer Review File are included in the article's Creative Commons license, unless indicated otherwise in a credit line to the material. If material is not included in the article's Creative Commons license and your intended use is not permitted by statutory regulation or exceeds the permitted use, you will need to obtain permission directly from the copyright holder.
To view a copy of this license, visit https://creativecommons.org/licenses/by/4.0/

Dear Dr. Stortz,

Thank you for the constructive comments you and the reviewers provided regarding our manuscript. We appreciate the time and effort that was put into the reviews, and believe that the suggestions and input have strengthened the manuscript. We have thoroughly revised the manuscript to address all of the reviewer comments and have provided line-by-line responses below in blue text.

Reviewers' comments

Reviewer #1 (Remarks to the Author):

Rodriguez-Ruano et al. investigate how sediment presence, burial depth, and burial duration affect coral larval settlement and recruit survivorship across several Caribbean species and life stages. They combine aquaria experiments with field surveys in Florida. The key findings are that even shallow burial layers significantly reduce settlement and survival across species, and that the presence of sediment in chambers reduces settlement in some taxa. They also run hazard models to simulate how multiple sediment events could impact recruit cohorts, concluding that sedimentation is a major bottleneck for coral population recovery.

This manuscript addresses an understudied aspect of coral ecology: the impact of sediment burial depth and duration on larval settlement and early recruit survivorship. The experimental work is valuable and relevant, but the paper is weakened by sensationalist language, occasional misrepresentation of context, and a lack of clarity in how results are reported. Cross-species comparisons are implied without statistical testing (that I can see), uncertainty is not reported for model predictions or hazard ratios, and the deterministic presentation of scenario modelling is problematic. Methods also need clearer justification, especially regarding sediment definitions and conversions.

We appreciate Reviewer #1's positive assessment of our manuscript and are grateful for the suggestions and feedback. We have thoroughly reviewed the manuscript to identify and remove language that could be taken as sensionalist or misrepresenting context. We have also overhauled the results section to more clearly report the results and address the Reviewer #1's comments regarding statistical reporting. Below we address each of Reviewer #1's comments.

Comments:

Line 60–64: The description of Bonaire's reef trajectory overstates Steneck et al. (2019). That study shows coral cover declined from ~45% to ~35% (a 22 percentage-point drop), not a halving. While parrotfish biomass was unusually high, the relationship between herbivory and macroalgal decline was correlative, not experimentally demonstrated. Also note that macroalgal cover at the final survey remained higher than at baseline. Reword to reflect these nuances and avoid over sensationalism (see Bruno et al., 2019).

Thank you for this suggestion; we have reworded this statement as follows (Now L59–64): "*Similarly, healthy assemblages of herbivorous fish on the Caribbean reefs of Bonaire correlated with a decrease in macroalgal populations after coral cover declined by 22% due to Hurricane Omar in 2008 and a bleaching event between 2007 and 2011. In turn, the abundance of suitable settlement substrate allowed for the recruitment of new individuals, growth of extant corals*[21]*, and recovery of corals to pre-disturbance levels (>45% coral cover; Ref.*[21]*) by 2017*."

Line 71–73: Consider acknowledging the increasing role of marine heatwaves in shaping coral recovery.

We agree that marine heatwaves have increasingly become a major factor influencing coral recruitment success and the subsequent recovery of coral populations. We added the following sentence (L73–74):
"*More recently, marine heatwaves have emerged as a major stressor limiting recruit survival and coral recovery across the globe*[39–41]."

Line 96–100: The term "paucity" overstates the literature gap. Soften by acknowledging existing studies and clarifying that the gap lies in multi-species, multi-stage Caribbean work.

We have softened the tone by rewording the sentence on (L99–101) and have added a reference supporting the statements made in the sentence.
"*Although studies documenting the impacts of sediments on corals have been conducted in the Caribbean*[72–76]*, the impact of sedimentation across multiple reef-building species and early life stages remains a significant knowledge gap in the region*[81]*.*"

Line 100: Typographical issue: ".Surprisingly" should read ". Surprisingly".

We have corrected this typo in the main text.

Line 103: Please acknowledge dredging and sediment burial studies, even if not controlled laboratory experiments.

We added a statement acknowledging field observations of the impacts of sediment burial on coral populations in the Caribbean (L106–109):

"*Although field observations have documented the effects of sediment burial on coral populations, no experimental studies that we are aware of have isolated the effects of sediment depth or burial duration on coral settlement and recruit survivorship across Caribbean species.*"

Line 107–112: The contrast between Caribbean and Indo-Pacific recovery should also note life history differences: Indo-Pacific reefs retain fast-growing branching taxa that recover rapidly, whereas Caribbean assemblages are dominated by slower-growing massive species and fewer opportunists. Include this alongside regional stressor context.

We decided to remove the recovery comparison with the Indo-Pacific to streamline the introduction and keep it concise and relatively short. We agree that Caribbean and Indo-Pacific coral communities differ due to a myriad of factors (species richness/functional redundancy, metapopulation connectivity, basin size, disturbance return period, etc.). Because contrasting coral communities between these two ocean basins is not an aim of our study, we felt that explaining them would distract readers from the main focus of Caribbean corals.

Line 109–112: Repeat bleaching events, driven by global climate change, are a dominant driver of mortality and must be acknowledged to avoid overstating sediment impacts.

We agree that the increase in the intensity and frequency of bleaching events is driving most of the coral decline worldwide. To this end, we have added a sentence stating that thermal stress is a major driving force in recruitment/recovery dynamics on contemporary coral reefs, per the comment for lines 71–73.

The aim of this paragraph is to narrow the topic to the specific stressors and factors that were the focus of this study, i.e., sedimentation. Therefore, we believe that restating that thermal stress and subsequent bleaching are major players in the decline of coral reefs would affect the flow of ideas in this paragraph and the rest of the introduction. We do not believe that the statement overstates sediment impacts, but rather highlights that they "may constitute a significant but underappreciated impediment to the recovery of increasingly disturbed Caribbean coral reefs". This language (i.e., 'significant') is less strong than our edits in the preceding section that underscore that recurring bleaching events are a major driver of coral decline and lack of recovery and is supported by the five references included at the end of the sentence.

Line 113–114: Rephrase too sweeping.

We have rephrased this sentence to avoid language that is too sweeping (L116–119):

"*Here, we present results of experimental tests on the effects of sediment presence, depth, burial duration, and source on larval settlement and recruit survival of six key reef-building species of Caribbean corals: Acropora cervicornis, Colpophyllia natans, Diploria labyrinthiformis, Orbicella faveolata, Pseudodiploria clivosa, and Pseudodiploria strigosa.*"

Line 149–151: Phrases such as "appeared to be the most resistant" are vague. If species-level differences are claimed, statistical tests are required. Otherwise, describe trends without implying significance. Your stated aim was to examine interspecific variation, but no direct comparisons are shown.

The reviewer makes a good point here. We have edited the text in the introduction (L119–121) that our aim was to assess the effect of sediment burial on multiple coral species and life history stages, and we have updated the statistical analysis to account for interspecific variation by adding "species" as a predictor variable to the GLMMs. The description of the updated analysis can be found in the *Statistical analysis* subsection of the Methods section (L687–L694):

"*For the larval settlement experiments, we conducted two binomial GLMMs with logit link functions: one to determine the influence of sediment presence around the substratum on the settlement success of coral larvae among multiple species, and a second one to determine the influence of burial of the substratum on the settlement success of coral larvae among multiple species. For each model, coral species and sediment load (mg cm^{-2}) were specified as fixed predictor variables and the replicates nested within treatments were specified as random effects. For the response variable, we used the proportion of larvae (0.0–1.0) that settled onto each replicate.*"

Results: No errors or confidence intervals are reported for settlement probabilities or hazard ratios. These should be included.

The errors and CIs for settlement probabilities and hazard ratios have been added to the main text of the Results section. We did not include them in the main text in our original submission because they are represented visually in the figure and we wanted to avoid cluttering the results section.

Results (Field surveys): Clear and well presented.

Thank you for the positive comment.

Figure 1g and 1i: For consistency, please show model fits.

We have updated Figure 1 to include the model fits for each sub-plot.

Line 253: The statement on sediment stress is overly simplistic. Sediment impacts are multifaceted and context dependent.

We reworded this sentence to make it clearer and concise relative to what was tested in this study (i.e., the impacts of sediment burial; L270–271):

"*This study thoroughly assessed the influence of sediment burial on larval settlement and recruit survivorship of multiple early life-stages across a suite of Caribbean coral species*."

Line 368–383: The scenario modelling reports numerical outcomes and reads as results?

We agree that this paragraph reports modeled data instead of interpreting trends; therefore, we moved this paragraph to the Results section at the end of the "sedimentation exposure assays" subsection. We were careful and explicit in stating that these data are predictive models based on simulated simulation events to avoid any misunderstanding that they were also lab- or field-based data.

Line 390–395: Avoid absolute language such as "sufficient to kill" and "plummets."

We have softened the language in both statements as follows (L425–426 and L430–431):

"*…and that burial under such sediment loads for 10 days can kill coral recruits up to 4 cm^2 in size (approximately 18 months old).*"

"*…recruit abundance on Florida's reefs sharply declines in the presence of LSAT sediment layers ≥4 mm deep*."

Line 440–452: Clarify how mass was converted to both mg cm^{-2} and mm burial depth. These conversions depend on tile area and sediment density, which should be stated.

We added the following statements to the second paragraph of the "Coral settlement assays" subsection in the methods to clarify our conversions (L473–480 and L484–489):

"*To create a gradient of sediment presence and depth of settlement substrate burial, we added enough sediment to 3.8 x 3.8 x 0.5 cm (L x W x H) ceramic tiles in seawater to create a 'sprinkle' on the tile (a thin veneer of sediment covering ~30% of the tile surface), a layer of sediment 2 mm deep covering 100% of the tile surface, and a layer of sediment 4 mm deep covering 100% of the tile surface (n = 10 per treatment). Once enough sediment was added to achieve these treatments (verified by measuring the sediment layers with a ruler), the sediment was carefully removed, dried, and weighed to standardize the amount of sediment added to each replicate for each treatment*."

"*To quantify sediment loads in mg cm^{-2}, we calculated the surface area of the ceramic tiles that we used as substrate for larvae to settle on (3.8 x 3.8 cm = 14.4 cm^2) and divided it by the weight of sediment for each treatment used to determine the sediment load in g cm^{-2} (example for 2 mm treatment = 2.15 g/14.4 cm^2 = 0.1489 g cm^{-2}). We then multiplied the sediment load in g cm^{-2} by 1,000 to convert it to mg cm^{-2}, which are the units commonly used in the literature*."

Line 463–464: If combining sediment sources, please report the statistical test used to conclude "no observed difference."

We have included a boxplot in the Supplementary materials (Fig. S10) and the statistical analysis that supports our claim (L516–520):

"*We ran a binomial generalized linear mixed-effects model (GLMM) to determine whether there were differences in larval settlement success among sediment sources (Key Largo [KL] vs. Port Everglades [PEV]), and there was no observed difference in settlement success between the source treatments (coefficient for "source" = -0.163, SE = 0.208, p = 0.43; Fig. S10).*"

General: Define what is meant by "sediment" in this study. Was grain size distribution, composition, or organic content quantified? Since these factors strongly affect burial and survivorship, they should be reported for the experimental sediments, not only in field

surveys.

We agree with the reviewer — we did quantify grain size distribution and include a figure depicting grain size distribution for each sediment source (Port Everglades and Key Largo) in the supplementary information (Fig. S7). We have added a statement in the Methods section highlighting the main sediment grain-size composition (third paragraph of the "Settlement assays" subsection to guide the reader to this supplementary figure if they would like to review the more detailed analysis of grain size distribution. We also include the sediment classification per the Wentworth grain size classification (with the associated reference) in the main text (L514–516).

"The sediment used in this study was predominantly of a grain size ≥500 µm (KL = 82%, PEV = 63%; Fig. S7), which is classified as medium to coarse sand by Wentworth[95]."

Reviewer #2 (Remarks to the Author):

I commend the authors on a well-designed and executed set of experiments! The paper is a nice contribution to the state of knowledge on the potential impacts of excessive reef sediments on the success of coral larvae in finding a suitable place to settle and survive as young recruits when buried by reef sediments. These are important topics in the discipline of coral reef science, as researchers and managers around the word seek solutions to aid coral reef recovery from mortality events caused by escalating ocean temperatures, diseases, etc. Outstanding features of the study are that five species of corals at various life stages were tested, including 2 that are threatened species, and all are important habitat-forming species essential for reef-ecosystem functioning. It is particularly informative to know that there are species-specific responses, indicating that some species are more at risk to excessive sediments than others. I have only minor suggestions for additional background information, clarity, and interpretation, which I include below.

We thank Reviewer #2 for their positive review of our manuscript and helpful feedback and suggestions. We have incorporated the edits and suggestions of Reviewer #2 in our revised manuscript and provide line-by-line responses to address Reviewer #2's comments below.

Title: The word "sedimentation" implies a rate (amount per unit time), so I suggest using "Excessive sediments may reduce…" and then fix subject-verb agreement so "Excessive sediments may reduce larval settlement, recruit survivorship, and the recovery of coral populations"

Reviewer #2 raises a good point here regarding terminology surrounding sediments and sedimentation. We agree that "sedimentation" may imply a rate rather than sediment loads, which is what we tested. We have changed the title to "*Sediment burial reduces coral larval settlement, recruit survivorship, and inhibits the recovery of coral populations*" to better express what was tested in our study.

However, by incorporating the word “excessive”, we would be implying that we tested sediment loads that are high for coral reef ecosystems. This would not be an accurate representation of the sediment loads used in our study, which based on published peer-reviewed literature (e.g., review by Tebbett and Bellwood 2019), are now commonly found within these ecosystems. Thus, we do not feel that including “excessive” in the title is appropriate.

Abstract: line 30, state early that this was an aquarium-based study with supporting field surveys. Last sentence is a little vague and yet too strong at the same time. I think your survey results suggest that the availability of suitable coral-settlement habitat is currently low in Florida, at least in the upper Keys, and the experiments showed that treatments simulating the accumulation of sediments on the reef substratum decreased coral settlement and recruit survival of 5 important habitat-forming species. The implications for managers section of the discussion needs a little work, so conclusion statement of abstract may need to be adjusted accordingly.

We modified the sentence beginning at line 30 to read as follows (now L29–32):
“*In this study, we conducted aquarium-based experiments with supporting field surveys to assess the response of larval settlement and recruit survival to the presence of sediment and the burial of substrata across six Caribbean species and multiple early life stages.*”

Introduction: Nicely written and well-cited. A couple little things.
Line 73, re: the two papers cited for “overgrowth by competitors,” citation 35 doesn’t document overgrowth, and instead suggests possible allelopathy, but citation 36 does encompass general algal-grazing-recruitment processes that could include overgrowth. This could be fixed easily by adding "allelopathy and/or overgrowth by competitors" to that sentence.

Thank you for this suggestion to make the sentence more accurate. We have added allelopathy as a process that can contribute to recruit mortality and it now reads as follows (L71–73):

“*For example, allelopathy or overgrowth by competitors*[35,36]*, incidental grazing by herbivores*[37]*, and predation*[38] *can all contribute to recruit mortality.*”

Line 84, What about type of source material? Grain size?

We modified the sentence to include those two additional sedimentation factors (L83–86):

“*Sedimentation can manifest as an acute or chronic stressor, with impacts on corals dependent upon the duration, magnitude, and return time of sediment stress events*[51,56,58]*, as well as sediment characteristics such as grain size and source material*[59,60]*.*”

Line 87, re: “paucity of Caribbean data,” See this pioneering study with caged and uncaged settlement blocks from the early 70s in San Blas Islands, Caribbean side of Panama: Birkeland, C. 1977. The importance of rate of biomass accumulation in early successional stages of

benthic communities to the survival of coral recruits. Pages 15-21 in Proceedings of the 3rd International Coral Reef Symposium, Miami. Paraphrasing from their study: "Mortality of recruits on upper surfaces was caused by the small corals being smothered in sediment. The filamentous algae did not directly overgrow the coral but created a sediment trap."

Thank you for providing the Birkeland (1977) reference. We edited this sentence based on reviewer #1's similar comments to dial back our language. We have also incorporated the suggested reference. The sentence now reads as follows (L99–101):

"*Although studies documenting the impacts of sediments on corals have been conducted in the Caribbean[72–77], the impact of sedimentation across multiple reef-building species and early life stages remains a significant knowledge gap in the region[78].*"

Line 129 and throughout paper, the word "substrates" is an awkward one that has been misused for decades. Enzymes work on substrates, and the bottom of the ocean is more properly the "substratum" (substrata = plural). I understand that the coral science literature has adopted the word substrate, but the use of it in the sentence stuck out to me as more odd than usual. How about "…the burial of the substratum and corals…"

Thank you for this suggestion; we agree that substratum is a better term for our context in benthic ecology. We have incorporated this suggestion into the sentence and substituted substrate/substrates for substratum/substrata throughout the manuscript. The highlighted sentence now reads as follows (L131–133):

"*We found that the burial of the substratum and corals under small amounts of sediment (150–300 mg cm^{-2}, equivalent to sediment layers 2–4 mm deep) caused drastic reductions in coral settlement and survivorship.*"

Line 140, "the need for avoiding and minimizing sediment sources," I suggest adding the word "anthropogenic," so "minimizing anthropogenic sediment sources." Managers will have little control over natural sediment-transport processes, and I have added comments below for the discussion to add some attention to the literature on these processes. Sediment resuspension from periodic wind/wave events can be a significant driver of LSAT-sediment depth, and the passage of hurricanes can also cause redistribution and un-compaction of soft sediments.

We have clarified the sentence referring to the sediment sources as "anthropogenic sediment sources".

Results:
Line 146, suggest "burial of potential settlement substratum."

This phrase was edited based on the previous comment regarding the same issue, and now reads as follows (L148–149): "*The burial of potential settlement substratum by sediment*

significantly decreased the probability of larval settlement for all five species of coral we tested (Figs. 1a–e, Table S1)."

Line 150 & 164, this a cool result about DLAB and the other species-specific responses! The results mirror habitat conditions where the species are found, like DLAB and PCLI having high abundances in the back-reef and lagoon areas where soft sediments are often deep, and reservoirs are nearby.

Thank you, we appreciate the positive feedback and agree that the species-specific responses were a really interesting result of our work.

Line 167, the "water quality" data and interpretations that result are my one main complaint about the aquarium experiments. The static (unstirred, I believe?) conditions inside the small vessels where the treatments were applied do not replicate the reef environment well, when it comes to exposure to and flux rates of nutrients. These were also sterilized sediments lacking the microbiome that remineralizes organics and drive N and P cycling on reefs. Please add some caveats about "water quality" and water-flow conditions in the experiments not being like those experienced on reefs to the discussion. Line 168, "high" concentrations of nitrate…how high were they? Please report mean and/or maximum measurements here. Also, Table 3 is not informative. Please change the table to include the raw data values measured in SI units.

We agree with Reviewer #2 that the conditions within our settlement chamber differ from those that coral larvae would experience in a natural setting and is a prime example of the unfortunate but necessary tradeoffs that often arise to effectively isolate single variables in controlled experiments (in this case, sediment depth). To ensure we explicitly recognize these important differences, we have added a paragraph in the discussion (L349–363) highlighting these water flow and quality caveats:

"*Lab-based studies that accurately emulate natural coral-reef environments are difficult to execute but important to understand how abiotic factors like water flow dynamics of the benthos and the complex biogeochemical processes that characterize sediment matrices influence larval settlement. Indeed, microbial assemblages within the sedimentary matrix and the hydrodynamics of the benthos play important roles in the remineralization of organic nutrients, the decomposition of detritus, and larval settlement cues, which can influence the survival and distribution of benthic macroorganisms[93–95]. The chambers where we conducted our settlement assays intentionally lacked water flow to allow us to establish and maintain our sediment burial depth treatments, and the sediments were sterilized with heat (oven dried) to prevent rapid declines in water quality in the chambers. Additionally, sterilizing the sediments allowed us to assess the sole contribution of the physical presence of sediments, isolated from any potential biogeochemical co-factors. Consequently, our results provide a baseline for the response of larval settlement to the physical effects of sediment deposition; however, future studies that can incorporate water flow or use 'live' sediments are important next steps to understand how these factors influence settlement rates.*"

Per Reviewer #2's suggestion, we have also updated the manuscript to report the mean ±SE values in the main text of the results and added a new table reporting these values for all treatments tested (Table S3).

Line 224, during what season were the surveys conducted in, and was the seasonal timing roughly the same? LSAT depth and turf-algal growth could be affected by season (e.g., wind, rain, wave conditions). Also, did these field observations drive the treatment levels for the sea-table experiments? It would be good, if they were, to justify treatment levels. If so, then maybe flip the order of how these pieces are presented in the methods and results.

Thank you for highlighting this important detail that was not included in our original submission. The LSAT surveys were conducted during May 2023, which is now specified in the Methods section. We also added a brief statement when reporting these results to explicitly state the time of year these data were collected. The treatment levels were defined based on discussions with the Florida Department of Environmental Protection and specialists at the NOAA Fisheries Southeast Regional Office to test levels relevant to future dredging operations in South Florida. These sediment depths, in particular, were based on the minimal amounts of sediment produced by potential dredging activities at Port Everglades (sediment layers 1–5 mm deep) that could impact the survivorship of juvenile corals in the vicinity of the project. Additionally, higher sediment loads were not tested because preliminary trials with (substantially) higher loads led to rapid decreases in water quality in the 100 mL jars used for this experiment. We added the following statement in the Methods section justifying the treatment levels (L489–L496):

"*These sediment depth treatments were selected to correlate with those measured on LSAT across our field sites (Fig. S5). Additionally, we wanted to test the impacts that minimal amounts of sediment could have on early life-history-stages in the context of proposed dredging activities in South Florida (sediment layers 1–5 mm deep Ref.111). Since the sediments we were working with were coarse (Fig. S7), the shallowest sediment layer that we could achieve while covering the entire substratum was 2 mm, and therefore this was set as our lowest threshold.*"

Line 227, do you think the variability was because of differential sampling times? Were there any timeseries data to back-up temporal persistence of the spatial patterns observed? Any previously published data to support these?

All sites were measured in May 2023 to control for seasonal variability and to test for spatial variability among sites. We originally stated this at the end of the first paragraph of the "Field surveys" subsection of the Methods. We moved it to the beginning of the paragraph to make it clearer to the readers, and we also include the survey timing in the Results section at the beginning of the "Field surveys" subsection.

Discussion:

Line 257, "changes" in water quality across treatments, do you mean differences among treatments? "Changes" imply timeseries data. Also, these differences are related to there being no water motion in the chambers, which is not very realistic. Maybe not in the second sentence, but somewhere here would be a good place to add the caveats about water motion in the vessels.

To address this comment and the one pertaining to L167, we have added a paragraph discussing the caveats of water flow and water quality to explicitly recognize this aspect of our study.

Line 264, "all but for one species tested." Specify by name, DLAB, here because this is an important result!
Thank you, we have specified that this species was DLAB at the end of the sentence.

Lines 286 – 289, as I mentioned above, LSAT and amounts and depths of sediments trapped could vary seasonally, with wind events creating windows of sediment resuspension and transport. I've observed and heard likewise from others who are in the field a lot, that strong hurricanes leave the reef sediments less compacted (i.e., "loose", where you can easily stick your arm up to your elbow in the sand). I'm curious if observations like this have been measured or published. This could be a possible mechanism for the reported overall trend of declining (or less accessible) suitable recruitment habitat on Florida reefs. Bigger hurricanes could be allowing more frequent and greater magnitude sediment-resuspension events. See Yates et al. https://doi.org/10.5194/os-20-661-2024. Another aspect of LSAT that isn't really mentioned is the turf-algal component. How has that changed over time? Are turfs growing taller than they used to (less urchin grazing) and catching more sediments, or did increase in sediments allow turfs to grow longer? There is potential for a positive feedback loop here.

The reviewer highlights a good point, which is that LSAT cover, sediment depth, and turf length likely vary seasonally, which could be driven by a variety of factors. As highlighted above in the previous LSAT seasonality comment, we conducted all of our surveys during the summer of 2023. At this time, however, it is still unknown to what extent LSAT parameters vary seasonally: are seasonal variations driven by turf length, sediment depth, or benthic cover. We added a sentence in the discussion where we highlight these knowledge gaps and the need for future studies to address them (L305-315):

"*It is evident that a positive feedback loop between sedimentary and algal turf dynamics contributes to the prevalence and stability of this ecological state (Ref.[90]; Fig. S6). The drastic loss of coral cover, coupled with high bioerosion rates[91], local dredging activities[3], and hurricanes[92] could be increasing the sediment load on Florida's reefs. However, the individual contributions of these stressors to the feedback loop are yet to be addressed. Furthermore, algal turf productivity and biomass vary seasonally, peaking in the summer and decreasing during the winter[93–95]. Therefore, LSAT characteristics at our sites likely exhibit some seasonal variability (we conducted our surveys during May 2023[96]. Future studies should address the*

spatiotemporal stability of LSAT and the interplay between algal turf productivity and sediment dynamics to better understand the mechanisms that underpin LSAT formation and persistence."

Regarding the role of hurricanes influencing sediment dynamics via redistribution and resuspension, we agree with the Reviewer. Hurricanes can deposit (or remove) large amounts of sediment on a reef. However, as Yates et al. and other studies (Li et al. 2002; https://onlinelibrary.wiley.com/doi/abs/10.1046/j.1365-3091.1998.00133.x) report, most sediments end up in the backreef and the sediment apron seaward from the forereef. All of our field surveys were conducted within shallow forereef platforms that were between 6–8 meters deep. The shallow fore reef platform is also the reef habitat that collects the least sediment from hurricanes, since they are usually flushed from these shallow habitats into deeper habitats (hence the formation of sediment aprons seaward of the fore reef). Moreover, the location where our study took place (Southeast Florida), has not experienced a major (or minor) hurricane event since Irma in 2017, six years before these surveys were conducted.

In addition, the LSAT sediment layer was never more than 6 mm deep. The sediment deposition values that Yates et al. report at Looe Key are several orders of magnitude higher than the sediment loads we recorded at our sites. It would be interesting to assess the influence of hurricanes on LSAT persistence, but that remains to be determined at this point.

Line 335, "contrary to our hypothesis…" Yes, but not that is unsurprising since bigger recruits respire more and would suffocate in these artificially still conditions. Could happen during large sediment redistribution event (hurricane), but on a day to day basis, I'd think most reefs are well flushed enough to keep that suffocation from happening for 10 days straight.

The Reviewer makes a valid point regarding the fact that larger recruits would respire more and thus may suffer from hypoxia/anoxia faster under burial conditions. We have removed the "contrary to our hypothesis…" wording to avoid controversy.

Regarding the 'artificially still' conditions, it may be possible that the reviewer is conflating the conditions in the settlement chambers (sealed with no water movement) and the recruit burial experiments, which were conducted in 20-gallon tanks that included water flow, though the water flow was necessarily positioned such that it did not suspend the sediments and nullify our sediment burial treatments (L611–616).

Regarding burial by sediments for extended periods of time - the level of reef flushing and thus ability to prevent suffocation from happening for 10 days straight depends on a myriad of factors - season, currents, wind, reef habitat, location within a reef habitat, etc. There are certainly locations that experience strong daily flushing, and others that are calm and may only experience flushing during some sort of event (e.g., strong winds). Beyond this, one of the main motivators of this study was to assess the potential impacts of sediment deposition on the early life history stages of corals resulting from dredging events that can generate large sediment plumes and deposition events, which can smother corals for days to weeks (e.g., Cunning et al. 2016).

Line 364, these results are likewise unsurprising, probably hypoxia. Effects of dissolved oxygen on coral early life stages deserves a sentence somewhere in here. See for example, https://doi.org/10.3389/fmars.2023.1254965. That reminds me, do you have any DO data from the experiments? People tend to overlook DO, but it can cause major issues if it drops below 2 mg/L, and in small, closed vessels, this is not unlikely at night if biomass is high.

Similar to the above, we agree with the reviewer's valid point regarding the likely role of hypoxia/anoxia in driving mortality, and agree the discussion of DO is warranted here. Unfortunately, measuring DO was beyond the scope of this study and thus we do not have DO data for these experiments. However, it is something we are incorporating into our current research (soon to be submitted for publication). Regarding the potential for DO to drop in the settlement chambers, this is a fair point. However, our 'sediment presence' treatment was employed as a sediment control — thus if decreases in DO were happening and this was causing issues for the coral larvae, this would have been reflected in those data (i.e., settlement rates for the 2 mm of 4 mm sediment present and sediment burial treatments would have been similar, which they were not). Moreover, we sterilized our sediments before using them in the trials as a measure to minimize potentially confounding biological or chemical factors and isolate the physical effects of sediment burial on larval settlement and recruit survivorship.

In addition, we added a paragraph in the Discussion section highlighting the importance of DO data in these experiments (L396–410):

"*The reduction in light levels and the high rates of microbial respiration within sedimentary matrices are some of the major biogeochemical mechanisms that contribute to coral stress and subsequent mortality*[59,107]*. Previous studies have shown that the mineralization of organic matter, driven by microbial respiration within reef sediments,*[13] *can induce hypoxic or anoxic conditions within the first 2–4 mm of the sedimentary matrix*[107,108] *—the depth range tested in this study— and induce coral mortality. Furthermore nutrient-rich sediments, often introduced from terrestrial systems, tend to promote higher respiration rates and deplete oxygen faster and at shallower depths than nutrient-poor reef sediments*[59]*. Although the effects of these biogeochemical mechanisms are known to be major contributors to coral mortality, few studies have quantified them within this context. Our study, for instance, did not measure oxygen concentration within sediment pore water because it was out of our scope —we were primarily interested in assessing how different sediment loads impact larval settlement rates and overall coral survivorship for multiple coral species. Nevertheless, it is essential for future studies to incorporate biogeochemical parameters to determine how the interactions between physical, chemical, and biological factors impact coral survivorship.*"

Conclusion:
Line 392, There's really no discussion of if there is a significant increase in sediment supply on Florida's or other reefs, but this is implied here. If this has been documented, please cite papers. Increased rates of erosion of carbonate framework as corals die maybe? See Lidz & Hallock, 2000, https://www.jstor.org/stable/4300079.

We agree with the reviewer that this is an important point to address. Based on previous suggestions, we added a brief discussion about it along with the potential positive feedback loop between turf algae and sediment (L305–310).

Line 396, “continued sedimentation,” From what source, are you implying terrigenous input? or dredging? Similar to my comment above, there was a school of thought among geologists in the last century that reefs in subtropical latitudes were just in a different spot in their trajectory (senescence) because of SLR and slowing down of reef accretion and an increasing pace of erosion. See NEUMANN, A.C. and MACINTYRE, I.G., 1985. Response to sea level rise: keep-up, catch-up, or give-up. Proceedings of the 5th International Coral Reef Symposium (Papeete, Tahiti). Volume 3, pp. 105–110. This is where the idea that reefs can be "shot in the back by their own lagoons" came from, meaning that reefs get choked out or clogged up by sediments when they run out of accommodation space or start to die and degrade for whatever reason.

We specified that we are referring to acute (dredging) and chronic disturbances (reef erosion and runoff). We added the following statement to the sentence to clarify this (L431–435):

“*This study highlights the serious consequences that continued sedimentation—from chronic disturbances such as reef erosion and runoff, and acute disturbances such as dredging—can have on the recovery of Caribbean coral populations by suppressing larval settlement and increasing the mortality risk of established recruits*.”

Line 298, same issue here. If sediment loads are from the back-reefs themselves, during resuspension events, then what could managers possibly do?

We agree that it is not feasible to manage processes such as sediment resuspension from hurricane events or chronic bioerosion pressure. In the last sentence of the paragraph, we specified that tangible actions managers can take are to minimize the impacts from dredging and beach renourishment.

Line 401, alternatively, managers could see that assisted-reproduction coral recruits were outplanted on reef topological highs that are better flushed and less likely to accumulate autochthonous sediments.

Although outplanting coral recruits in areas with high topography would improve the survivorship of those corals, the lower-relief areas dominated by LSAT and susceptible to sediment burial, would prevent any further natural recruitment. We want to highlight the idea that improving habitat quality, by reducing sediment loads on reefs, is necessary to promote the natural recovery of coral populations.

Methods:
Line 420, the paper is very repetitive in places. Goals are stated numerous times.

We have removed the repetitive paragraph and restructured the first paragraph of the Methods to make it less repetitive (L445–458):

"To address our questions, we coupled aquaria-based experiments using larvae and coral recruits generated from 2023 coral spawning collections with field surveys conducted during May 2023 on coral reefs in Florida, USA. In this study, we define coral recruits as any settled and metamorphosed coral (from the planula larval stage to the polyp stage) of 1–18 months of age. When the age of the coral colony is unknown, we define recruits as a coral colony with a maximum length of ≤4 cm. We conducted a series of aquaria-based experiments to address two main goals: (1) understand how burial of the substratum influences coral settlement, and (2) understand the effect of sediment burial depth and duration on recruit survivorship across different life history stages. Multiple species and life history stages were used to tease apart species-specific responses to sedimentation and determine how these responses may vary as a function of coral age. The main objectives of the field surveys were to assess the variation of benthic assemblages and sedimentation rates on coral reefs in southeast Florida across a north-to-south gradient and identify any potential relationships between ecological and sedimentological processes."

Line 443, more details are needed about the settlement tiles. What were they made up, and were they conditioned in any way? Were the undersides available to larvae?

The details of the tiles are specified in the paragraph starting at line 467 of the original submission. We added a sentence specifying that tiles were conditioned in our site at Key Largo for approximately 2 months before the start of the experiment (L523–526):

"*Each chamber received a single 3.8 x 3.8 x 0.5 cm (L x W x H) ceramic tile (Boston Aquafarms, Boston, MA USA). All tiles were seasoned in the field for two months prior to the start of the experiment at the same site where we collected Key Largo sediment (Fig. 4b). Once a chamber received a tile, it was filled with 100 ml of filtered (20 µm) and UV-sterilized seawater originating from Bear Cut, Miami, FL USA*."

Line 445, what about grain size and size uniformity, was this kept constant?

We originally designed this study to test the effects of sediment originating from Port Everglades, regardless of grain size, on larval settlement and the survivorship of juvenile corals. We wanted to use the sediment that was present at the port without segregating grain sizes to establish baseline trends with the sediment that naturally occurs there. We also have a figure in the supplementary materials (Fig. S7) that shows the relative abundance of different grain sizes for the sediments we used in our settlement and recruit survivorship assays. Overall, for both Port Everglades and Key Largo sediments were coarse, with the ≥500 µm fraction being the predominant one for both sediment sources.

Line 462, sediments were sieved, but were they sorted by grain size? If so, what fractions were used? Was this silt or sand?

The sediments were only sieved to remove large grains (anything >5 mm), but the rest of the sedimentary matrix was used. We added a sentence with more details of the sediment used based on other reviewer comments:

"The sediment used for this study was predominantly of a grain size ≥500 µm (KL = 82%, PEV = 63%; Fig. S7), which is classified as medium to coarse sand by Wentworth[109]."

Line 485, Was there any stirring or mixing? Not very realistic conditions, if none.

We did not include any stirring or mixing given that constant stirring/mixing was not viable given the experimental design (sediments placed on top of settlement substratum). While we understand that this would be an ideal experimental setup, it was not feasible to include in our experimental design given the sediment treatments (more below) and the high number of replicates conducted (i.e,. Hundreds of chambers). In this light, intermittent stirring/mixing would have been the only option. However, intermittent stirring and mixing was not conducted for the settlement assays for several reasons: (1) Water movement sufficient to mix the water within the chamber would likely have dislodged sediment from the settlement substrata and thus nullify our ability to replicate sediment burial depths across so many replicates; (2) Mixing and stirring would likely affect larvae and may dislodge them if they are in the early stages of settling, confounding our settlement results. Moreover, we employed a large (100 mL) volume of water for coral larvae settlement assays specifically to avoid water quality issues. Often, larval settlement assays are done in small well plates (i.e., 1–5 mL). Although loose sediment can be resuspended, we were emulating situations when sediment is trapped on the benthos, either by surrounding structure that can create sediment pockets or by long turf algae, which would form LSAT; a stable algal turf and sedimentary matrix.

Lines 508 – 513, please report the detection limits of these instruments and analyses. Were they able to measure micromolar levels of these water quality metrics (nutrients were very low, no doubt).

We have included the detection limits in the paragraph. Our ammonia reagent was able to detect at least 10 µg/L (detection limit = 0.01–0.50 mg/L NH_3-N), our nitrite reagent was able to detect at least 2 µg/L (detection limit = 0.002–0.300 mg/L NO2- -N), our nitrate reagent was able to detect at least 300 µg/L (detection limit = 0.3–30.0 mg/L NO3- N), and our sulfide reagent was able to detect 5–800 µg/L.

Line 562, state when (dates) these surveys occurred for each location.

We included the dates in the sentence below where we list all six sites (L634–638):

"*Our sites include Fort Lauderdale (Broward County; surveyed on May 10, 2023), Dania Beach (Broward County; surveyed on May 9, 2023), South Canyon (Miami Beach; surveyed on May*

11, 2023), Emerald Reef (Key Biscayne; surveyed on May 16, 2023), Carysfort Reef (Key Largo; surveyed on May 24, 2023), and Conch Reef (Key Largo; surveyed on May 25, 2023; Table S5)."

Lines 569 and 573, were sites and spots to measure LSAT truly randomly selected, or was it haphazard? Sounds haphazard. If truly random, include description how assigned (e.g., using a random numbers table, roll of dice, coin flips, etc.).

Indeed, it was haphazard. We edited the sentence to reflect that (L640–641):

"*At each site, we haphazardly outlined a permanent 25 m transect oriented from north to south using the surveyor's tape*."

Line 591, was this the same 14 day period for all sites? If not, I'm sure there'd be a lot of temporal variability depending on wind and waves.

We aimed to leave the sediment traps out for a period of approximately 14 days, and most traps were left out for only 1 or 2 days more or less than that target except for South Canyon, which was left out for 29 days; those sediment traps were not retrieved near the 14-day window because of poor weather conditions:

Ft Lauderdale: 15 days
Dania Beach: 15 days
South Canyon: 29 days
Emerald Reef: 16 days
Carysfort Reef: 13 days
Conch Reef: 12 days

However, as seen in Fig. 4d, the calculated sedimentation rates for this site exhibit the same range of values and variation as the sediment traps of nearby sites that were left out for 15 days (Dania Beach and Fort Lauderdale). Since sediment traps at our southernmost sites (Emerald, Carysfort and Conch) were left out closer to the 14-day baseline and within the same season and year (May 2023), the higher sedimentation values at our southernmost sites likely reflect variations due to higher sediment fluxes within that time of the year rather than variations in wind and waves.

Statistics section: State upfront what statistics software was used, and if R, what packages. How were assumptions of the models evaluated? These are stated for some tests, but not others.

We stated upfront that R was used for analyses. Additionally, we stated all packages that were used at the beginning of the sentences where we describe the analyses that required those packages.

Figures and Tables:
Fig. 1. State what the data points represent. Each point represents a single vessel or is a mean of replicate vessels in the same treatment? I understand that the statistical models were chosen to deal with high variability, but could the raw data points (proportion of larvae settling with each vessel) still be shown on the plots? The binary response can still be used for statistics, but it would be more interesting to see where the data fall.

Each point represents a single vessel, and we have updated the figure caption to include this:

"*Each data point represents a single replicate, which consist of the glass chamber with a ceramic tile, 10–15 coral larvae, and a designated sediment load between 0–300 mg cm^{-2}.*"

Supp. Material:
Table S3: I'd rather see the raw data values in SI units so we can judge for ourselves the ecological relevance of the studies (are these WQ measurements within normal ranges reported in the field?). What are the units reported for "average" column? Negative numbers suggest these are not uM values. If data are reported in SI units, then just the p-values of these contrasts could be included. These other stats numbers are not useful.

We added a new table (now Table S3) that shows the raw data values in SI units for each water quality parameter for each treatment. The SIMPER output table is now Table S4.

I hope you find my comments helpful and constructive! I enjoyed reading the paper and it hits on a lot of important processes in the recovery (or not) of FL reefs.

Thank you, the comments and feedback were helpful and definitely improved the manuscript.

Reviewer #3 (Remarks to the Author):

Overarching comments
Well written and engaging overall, congratulations to the authors. A relatively straightforward study confirming negative effects of sediment on larval settlement and survival through early life stages in six Caribbean coral species. Findings are consistent with studies in other regions although not well documented in Florida. The study makes useful links to water quality management and will provide an important contribution to the literature on the effects of sediment load on coral recruitment in Floridian reefs.

I have some minor editorial suggestions, noted below in Specific Comments. An overall comment is that the manuscript is very long given the study - while good - is fairly straightforward. This comprehensive approach is appropriate in a thesis chapter where all the extras are needed to satisfy examiners, but for publication, it would benefit from some trimming to reduce wordage and thus improve readability and impact. This would be particularly beneficial in the methods and results sections.

We have thoroughly edited the manuscript to remove redundancies and streamline the paper as much as possible, while adequately addressing all of the comments received from the 3 reviewers and editor.

The introduction and discussion are well reasoned, with good connections to water quality management challenges. However, some discussion paragraphs repeat results in more detail than necessary. These would be improved by a sentence or two focusing on the main finding of interest for comparison with other studies, rather comparing direct results.

We thank Reviewer #3 for their positive assessment of our manuscript. Please see below for line-by-line responses to specific comments.

Specific comments

Abstract
Overall nice abstract, concise and clear.

L29 – "…there is a surprising lack of information…" Suggest replacing "surprising" with "limited". Whether or not something is surprising depends on the point of view.

We have reworded this sentence to include the discrete language suggested:

"*Although sedimentation has been identified as a major stressor for corals, information on its influence on juvenile corals in the Caribbean is limited.*"

L31-32 – "…across multiple Caribbean species…" quantifying this number would be more informative, suggesting replacing "multiple" with "six".

We have replaced "multiple" with "six" here.

Introduction
Overall solid intro, well written and sets the scene effectively. Clearly articulates the management relevance of the study.

L46 – "coral-killing" might be a bit strong. Declines are also caused by disturbances that limit growth and disrupt reproduction, without necessarily causing direct mortatily.

We disagree Reviewer #3 on this point. Death is a requirement for a coral population **decline** - i.e., loss of individuals from the population. In contrast, limiting growth or reproduction, as suggested by the reviewer, do not cause declines in a coral population but rather can serve to reduce or inhibit population increases, but these are not synonymous processes.

Despite the semantic nuance noted above, we have reworded this sentence to clarify that disturbances can be lethal and sublethal (now L44–45):

"*Coral populations are in decline worldwide as a result of global and local stressors that continue to increase the frequency and severity of lethal and sublethal disturbances for corals*[1–5]*.*"

L48-52 – Great summarising sentence.
Thank you.

L52 – "Recruitment, survival and growth into reproductive adults…" Suggest replace "adults" with "maturity". However, this sentence is a little clunky. Arguably, recruitment IS growth to reproductive maturity, so this may need to be recast depending on how the authors define recruit/recruitment (more comments on that later).

Reviewer #3 makes a good point here and below regarding what comprises recruitment and the importance of defining this process. We felt this was an appropriate place to define recruitment and have edited the sentence to read as follows (L51–52): *"Larval settlement, survival, and growth into reproductive maturity (i.e., recruitment) are fundamental to the recovery of coral populations after mass-mortality events*[19–22]" , which also includes replacing "adults" with "maturity".

L56 – "…multitude of factors…" there's a figure in Randall et al 2020 that summarises these neatly, might be a good citation here (Sexual production of corals for reef restoration in the Anthropocene, in MEPS).

Thank you for this suggestion, we have included this reference in the sentence.

L100 – "Surprisingly" again, suggests delete, for same reasons as L29.

We have reworded this sentence as follows (L104–106):
"*Yet studies that focus on the early life-history stages of Caribbean corals, the life stages at which they are likely the most vulnerable to the impacts of sedimentation*[49,73–75]*, are sparse*[76,77]*.*"

L109 – Suggest replacing "futile" with "poor".

We edited this sentence to remove 'poor' and highlight that the recovery of coral populations in the Caribbean has been limited.

L131 – "Surprisingly" again! Not sure it is good fit here either. The study findings are definitely interesting, but are they really surprising? They sound generally consistent with the predictions in lines 125-127. Suggest delete.

Deleted.

Results

Overall, nicely written, as is the whole paper, but very long and would benefit from tightening up to give greater focus on the interesting results.

L172 – Typo at end of line “the the”.

Fixed.

Discussion

L257 - “… the effects of sediment burial…” Should this be “sediment burial of substrates”? Suggest clarifying. Parts of this sentence seem repetitive, plus it is very long and would be more readable if made into two.

We changed “effects of sediment burial” for “the burial of substrata by sediment” and reworked the long sentence to improve its clarity (L274–277):

“*Moreover, the strong adverse effects of sediment burial were consistent across all species and life histories tested. For coral larvae, the burial of substrata by sediment was the main driver of decreased settlement, evidenced by the strong response of larvae to the burial of settlement substrata across all species.*”

L260 – Not sure this is “surprising”, but it does contradict the phrase preceding. Please review.

We removed all instances of the ‘surprisingly’ in the manuscript. For this specific instance, we have edited to specify that the adverse effects of “sediment burial”, not necessarily “sedimentation” were consistent across all species (L274–275):

“*The strong adverse effects of sediment burial were consistent across all species and life histories tested*.”

L269 – At the end of this line it implies the findings are applicable to all species, which is an overreach. Suggest replacing “a coral’s” with “these coral species” or something similar. Given dominant species found in field (Siderastrea and Porites) were not among those investigated in this study, consequences of sediments may not be as pervsive for them.

That is a good point, we reworded the sentence to specify that the consequences are pervasive for the species tested (L284–286):

“*Together, our findings highlight that the consequences of sedimentation are pervasive for at least the first 18 months of age for the species tested and have the potential to severely diminish the capacity for coral populations to recover.*”

L281 – Instead of “… induce severe declines or inhibition of…”, suggest just say “Inhibit”.

We replaced “inhibition of…” for “inhibit”. We do think it relevant and important to keep the “induce severe declines” component of the sentence to avoid overextending/sensationalizing our findings and implying that low sediment loads completely inhibit settlement for all species tested.

L289 – Might be a local way of saying this, but consider making the word “reef” after Florida’s plural?

Updated to “Florida’s reefs”

L290 – why is it “surprising” that field surveys corroborated lab observations?

Updated text to remove “surprising”

L300 – “bottlenecks” might be used out of context here, suggest “inhibition” might be more appropriate.

Updated text to replace “bottlenecks” with “reductions”, which we feel is the most appropriate term for this instance.

L318-320 – Good point well made.

Thank you.

L325-329 - Repeating results here and throughout this para. Would be more concise (and therefore compelling) just to strongly summarise the key finding for each point you're making.

We summarized these points into the following sentence (L366–369):

“Here, we found that burial under 4 mm of sediment severely reduced the survival probability of all species and age groups tested except for 1-month-old C. natans recruits, which still experienced a severe reduction (68%) in probability of survival.”

L353-354 – Nice way to contrast different findings. It’s helpfully complementary to have two different sediment studies investigating alternate sediment deposition scenarios and finding different outcomes. Lots more to learn in this space.

Thank you.

L368-383 - Suggest toning down some of the modelled assertions, the reasoning is sound, but still speculative where they extend beyond what was explicitly measured in this study.

We edited the paragraph to tone down the assertions and moved the paragraph to the Results section based on other reviewers comments. It is now the last paragraph of the Sediment Burial Assays section in the Results.

L372 – mention of “coral recruits”, given this term is not used uniformly across coral literature and scientists, please clarify your definition in the Methods and then ensure you use that consistently throughout. Some more comments on this below.

We provided our definition for coral recruit in the second sentence of the Methods as follows (L447–450):

“In this study, we define coral recruits as any settled and metamorphosed coral (from the planula larval stage to the polyp stage) of 1–18 months of age; when the age of the coral colony is unknown, we define recruits as a coral colony with a maximum diameter of ≤4 cm.”

Conclusion
Really nice conclusion summarising this neat and interesting study.

Thank you.

L399 – Suggest not using “bottlenecks”, it doesn’t fit well here. “inhibitors” may be an alternative to consider.

We respectfully disagree with the reviewer here. We believe that “bottleneck” is more appropriate because it expresses a reduction in settlement rates. “Inhibitor”, on the other hand, is a strong statement that suggests that no settlement is occurring.

L403 – Great to link this work to management priorities, however it’s an overreach to imply that improved sediment management could “increase” survival and population recovery, as other external factors could override that. Suggest replacing with “improve” or something similar.

We replaced “increase” with “reduce factors impacting”

Methods
L429 – “Settlement assays were performed in individual glass chambers” Can a different word or alternate phrasing be found for this? Strictly speaking, "individual" could mean individual larva, but there were 15-20 larvae per assay. As a suggestion for authors to consider, perhaps "Each assay was performed in individual glass chambers..." ??

We reworded the sentence as follows (L462–464):

“Each replicate trial for the settlement assays were performed in an individual glass chamber (118 mL capacity, 6.5 x 8 cm, diam. x H) that were placed into water baths (35 L capacity, 62 x 43 x 17 cm; L x W x H) in a random block design.”

L445 – reference to Fig S5. Not clear on the direct relevance of this figure here? If authors are implying sediment loads were selected for correlation with sediments loads associated with LSAT across field sites, it would be helpful to articulate that plainly.

We added a few sentences here articulating this more clearly (L489–496):

"*These sediment depth treatments were selected to correlate with those measured on LSAT across our field sites (Fig. S5). Additionally, we wanted to test the impacts that minimal amounts of sediment could have on early life-history-stages in the context of past and planned dredging activities in South Florida (sediment layers 1–5 mm deep Ref.112). Since the sediments we were working with were coarse (Fig. S7), the shallowest sediment layer that we could achieve while covering the entire substratum was 2 mm, and therefore this depth was set as our lowest threshold.*"

L479 – "After establishing sediment treatments…" Please clarify how long the sediments had to settle before larvae were introduced. This is relevant to the interesting comparison with another sediment study at L353.

Since our sediment was mostly coarse sand (≥500 μm), it had all settled approximately 1 hour after placing it in the jar. We added a statement to the sentence clarifying this (L536–539):

"*After establishing sediment treatments and waiting at least 1 hour for the sediment to settle, coral larvae were placed into each settlement chamber (n = 20 larvae/chamber for Orbicella faveolata and Diploria labyrinthiformis, n = 15 larvae/chamber for Colpophyllia natans, Psuedodiploria clivosa, and P. strigosa)*."

L515 - This is relevant to the whole manuscript. It would be worth defining what you mean by "recruit" either here (or somewhere). There's not a clear definition of what stage "recruit" refers to in corals. Some use it to mean corals that have finished the process of recruiting to the reproductive adult population, others use to for corals over 2 months of age, others use it for juvenile corals <5cm diameter. Peter Edmunds gives a useful summary of this in his 2023 review in Biological Reviews (page 1864). https://onlinelibrary.wiley.com/doi/10.1111/brv.12987

Based on previous comments, we included a sentence in the Methods section where we define what we mean by "recruit":

"In this study, we define coral recruits as any settled and metamorphosed coral (from the planula larval stage to the polyp stage) of 1–18 months of age; when the age of the coral colony is unknown, we define recruits as a coral colony with a maximum length of ≤4 cm."

L525 – on species recruit availability: This is a helpful clarification. Please also provide a short explanation of where these recruits came from and their health status prior to the burial treatment (presumably they weren't the survivors from the settlement assays??).

The origin of coral recruits was included in line 540 of the original manuscript submission (now L602–604). We added an additional sentence to clarify that the source of the OFAV fragments and that the recruits used were independent of the larvae that were used in the settlement assays.

L548 – on temperature consistency at ~28oC, except the figures show drops in temperature on Day 2 for CNAT and OFAV. Perhaps provide brief reasoning if this was not deemed significant.

We added a statement explaining this caveat (L616–620):

"*In the trials for the 3-month-old C. natans and O. faveolata, there was a decrease in the average seawater temperature of ~1.5 °C within days 2 and 3 of the experiment. However, this temperature change is within typical observed temperature fluctuations that corals experience on Florida reefs and was not associated with an increase in mortality during our experiment.*"

L553 - Instead of "born", maybe "spawned"? Don't think "born" is appropriate term where broadcast spawning species are included.

We replaced "born" with "spawned".

L554 – Maybe a typo, should there be a comma between 2024 and coral?

We fixed the typo.

Tables and Figures
Well presented and east to interpret,

Thank you.
